# MIMIC: an optimization method to identify cell type-specific marker panel for cell sorting

**DOI:** 10.1093/bib/bbab235

**Published:** 2021-06-26

**Authors:** Meng Zou, Zhana Duren, Qiuyue Yuan, Henry Li, Andrew Paul Hutchins, Wing Hung Wong, Yong Wang

**Affiliations:** Department of Mathematics, Huazhong University of Science and Technology, Beijing 100190, China; Department of Genetics and Biochemistry, Clemson University, Beijing 100190, China; Academy of Mathematics and Systems Science, CAS, Beijing 100190, China; Department of Health Research & Policy, Bio-X Program Stanford University, Beijing 100190, China; Southern University of Science and Technology, Beijing 100190, China; Department of Statistics, Department of Biomedical Data Science, Bio-X Program Stanford University, Beijing 100190, China; CEMS, NCMIS, MDIS, Academy of Mathematics and Systems Science, Center for Excellence in Animal Evolution and Genetics, University of Chinese Academy of Sciences, CAS, Beijing 100190, China

**Keywords:** cell type-specific marker, TFs, surface markers, dimension reduction, hierarchical topology

## Abstract

Multi-omics data allow us to select a small set of informative markers for the discrimination of specific cell types and study of cellular heterogeneity. However, it is often challenging to choose an optimal marker panel from the high-dimensional molecular profiles for a large amount of cell types. Here, we propose a method called Mixed Integer programming Model to Identify Cell type-specific marker panel (MIMIC). MIMIC maintains the hierarchical topology among different cell types and simultaneously maximizes the specificity of a fixed number of selected markers. MIMIC was benchmarked on the mouse ENCODE RNA-seq dataset, with 29 diverse tissues, for 43 surface markers (SMs) and 1345 transcription factors (TFs). MIMIC could select biologically meaningful markers and is robust for different accuracy criteria. It shows advantages over the standard single gene-based approaches and widely used dimensional reduction methods, such as multidimensional scaling and t-SNE, both in accuracy and in biological interpretation. Furthermore, the combination of SMs and TFs achieves better specificity than SMs or TFs alone. Applying MIMIC to a large collection of 641 RNA-seq samples covering 231 cell types identifies a panel of TFs and SMs that reveal the modularity of cell type association networks. Finally, the scalability of MIMIC is demonstrated by selecting enhancer markers from mouse ENCODE data. MIMIC is freely available at https://github.com/MengZou1/MIMIC.

## Introduction

Cell sorting aims to separate a heterogeneous mixture of cells according to an intracellular process (e.g. DNA, RNA and protein interaction). Accurate sorting of cells is crucial for many aspects of biological and clinical practice and is a driving force in the move from population-based studies to single-cell studies [1, 2]. The success of cell sorting is highly dependent on the marker panel used to distinguish different cell types. In the hematopoietic system, specific cell surface markers (SMs), and particularly the ‘cluster of differentiation’ CD antibody series, have been highly successful in enabling the elucidation of specific developmental steps in hematopoietic development [3]. Transcription factors (TFs) specifically mark cellular lineages and can also be used to purify distinct cellular populations that emerge during *in vitro* endoderm differentiation of human embryonic stem cells by multi-channel fluorescence-activated cell sorting (FACS) [4]. The ability to classify other developmental lineages in a rigorous manner would be a significant advance for developmental biology and for regenerative medicine, which greatly depends upon understanding and selecting pure populations of precise cellular types.

Thanks to the large consortia such as ENCODE, Roadmap Epigenomic projects and FANTOM5, extensive data on transcript abundance are available across many tissues and cell types. These rich data offer an exciting opportunity to dig out cell type-specific marker panels for cell sorting. However, one key challenge is complex hierarchical topology that the cell types are organized into. In addition, the candidate marker genes are buried in very high-dimensional data, for example ENCODE provides tens of thousands of genes’ expression profiles by RNA-seq, and the millions of functional elements from DNA accessibility profiles [5]. This may lead to the well-known ‘curse of dimensionality’. How to overcome these difficulties and identify cell type-specific markers remains a problem that needs further study.

Many approaches have been proposed to identify cell type-specific marker genes that can discriminate each cell type [6–9]. They mainly identify the cell type-specific genes based on their high and specific expression in a small number of cell types by comparing with other cell types. For example, Yu *et al*. [7] developed a computational approach based on expression enrichment and statistical significance for each gene in each tissue. Liu *et al*. [6] developed a database named TiGER (Tissue-specific Gene Expression and Regulation) based on this method. However, almost all these methods focused on each gene in each cell type and thus do not fully consider the genetic interactions among molecules and the hierarchical topology of cell types. Pierson *et al*. developed a tissue-specific gene regulation network based on the prior information of relationship between different tissues, but the parameter space was large, which might lead to incorrect or difficult to interpret results [10]. In addition, the combinations of makers required to distinguish different cell types were not fully considered. Recently, Newman *et al*. [11] proposed a computational approach to identify the composition of complex tissues using gene signatures, but the biological meaning of these signatures remains unknown.

In this paper, we provide a novel method, Mixed Integer programming Model to Identify Cell type-specific marker panel (MIMIC), to identify cell type-specific marker panels for cell identification and sorting. MIMIC selects an optimal set of cell type-specific markers while maintaining the hierarchical topology of the cell types. The cell type-specific markers could distinguish different cell types and enhance the biological interpretation. Additionally, the hierarchical relationship could demarcate similar cell types. Specifically, we implemented a mixed integer programming method to identify an optimal panel with a fixed number of selected markers *k*. By varying the parameter *k*, we could get different panels to distinguish different cell types then selected a relatively small number of markers as a panel. MIMIC is distinct from existing approaches in that it (i) utilizes the topology among cell types to select marker panels, (ii) takes a rigorous optimization framework to find an exact solution, (iii) combines SM and TFs together and (IV) improves the computation scalability by using a linear programming relax.

We systematically applied MIMIC to the RNA-seq dataset for 29 diverse tissues in mouse ENCODE [12] and then extended to a larger RNA-seq collection including 231 cell types [13]. MIMIC outperforms the existing methods in accuracy and in biological interpretation. In addition, the selected marker panel with SMs and TFs provides a rich resource for cell soring. Finally, we extended MIMIC to a linear programming method, which showed good performance in selecting cell type-specific enhancers as potential markers.

## Methods

### Overview of the optimization model MIMIC

**Figure 1:**
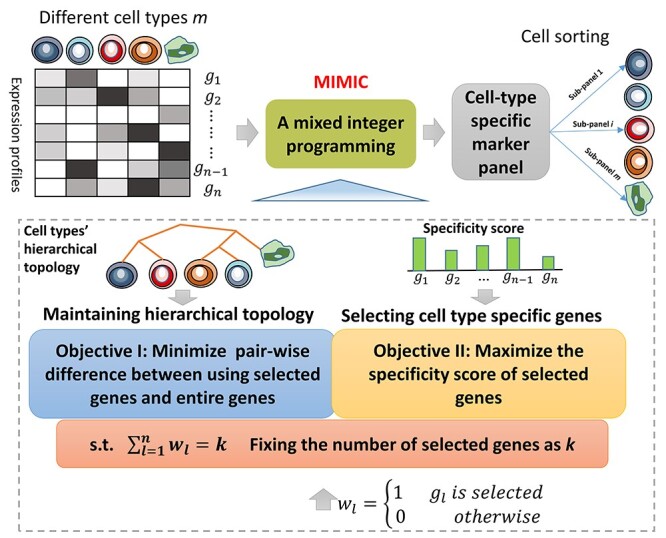
Flowchart of the operations of MIMIC. The gene expression profiles of different cell types are obtained from RNA-seq. A mixed integer programming MIMIC is proposed to identify the cell type-specific marker panel. Specifically, MIMIC optimizes two objectives: maintaining the hierarchical cell type topology and selecting cell type-specific genes. Simultaneously, MIMIC prefers to select the smallest marker panel via minimizing the number of selected genes. Finally, the cell type-specific genes combination selected could help in cell sorting.

We propose a MIMIC to distinguish different cell types (Figure 1). The model aims to maintain the hierarchical topology among different cell types and simultaneously select the minimal markers with the best cell type specificity. To maintain the hierarchical topology, we minimize the total and pairwise cell type difference calculated with the selected markers and the entire set of markers. Simultaneously, we maximize the sum of specificity score for the selected genes. Finally, two parameters are introduced to combine the three objective terms into a mixed integer programming model. Solving the model, we could obtain a cell type-specific marker panel for the potential follow-up cell sorting.

### Quantification of difference between cell types

Given the gene expression profiles ${S}_{mn}$ with $m$ cell types and $n$ genes, where ${s}_{il}$ is the gene expression level for a gene $l$ in cell type $i$, $i=1,2,\cdots m;l=1,2,\cdots n$. Specifically, ${S}_i$ denotes the expression profile for cell type $i$ and the difference between two cell types is given by the following fold change for the gene $l$,

(1) \begin{equation*} {y}_{ijl}=\left\{\begin{array}{@{}l}\frac{S_{il}+1}{S_{jl}+1}\kern3em if\ \frac{S_{il}}{S_{jl}}>1\ \\{}\frac{S_{jl}+1}{S_{il}+1}\kern3em \mathrm{otherwise}\end{array}\right. \end{equation*}

where $j=1,2,\cdots m$. When ${y}_{ijl}$ is a bit larger than 1, then it is hard to distinguish the two cell types $i$ and $j$ by gene $l$. It is reasonable to assume that ${y}_{ijl}$ larger than 4 is good enough to distinguish two cell types by gene $l$. Therefore, a smoothed sigmoid function is utilized to transform the fold change scores ${y}_{ijl}$ within a specified range. When the fold change score tends to be 1, the function value tends to be zero. When the saturation begins, the growth slows down. At maturity, growth stops. Here, we take the following sigmoid function to meet the requirement. Concretely, we define the difference between two cell types for gene$l$ as follows.

(2) \begin{equation*} {z}_{ijl}=\frac{1}{1+\exp \left(-\theta \left({y}_{ijl}-{y}_0\right)\right)} \end{equation*}

Where $\theta$ is a parameter to adjust the tendency for ${z}_{ijl}$ tends to be 0 when ${y}_{ijl}$ tends to be 1. Larger$\theta$ means that ${z}_{ijl}$ tends to be 0 in a faster way when ${y}_{ijl}$ tends to be 1. ${y}_0$ is the sigmoid’ s midpoint for ${y}_{ijl}$ value. Here, we set the default $\theta =10$, ${y}_0=3$ then ${z}_{ijl}$ tends to be $2.1\times{10}^{-9}$ (nearly to zero) when ${y}_{ijl}$ tends to be 1.

### Cell type specificity score for each gene

To evaluate the cell type specificity for gene $l$ in cell type $j$, we separate cell type $j$ from other cell types and treat other cell types as background. Then, we construct two vectors, observed vector ${u}_{jl}$ and idealized pattern ${v}_{jl}$, and compare the difference of ${u}_{jl}$ and ${v}_{jl}$ by calculating score ${ds}_{jl}$. The observed vector ${u}_{jl}$ consists of the expression level of cell $j$ and the background. The expression of background cell type is obtained by the third quartile of the cross-context expression levels of the gene $l$. The elements in this vector are divided by the sum so that the normalized vector sums up to 1. For the idealized pattern ${v}_{jl}$, the vector was formed by setting 1 in cell $j$ and zeroes in the background cell types. Then, the difference score to evaluate the two vectors could be calculated by the entropy-based measure of Jensen–Shannon divergence (JSD):

(3) \begin{equation*} {ds}_{jl}=\mathrm{JSD}\left({u}_{jl},{v}_{jl}\right) \end{equation*}

Then, we define the specificity score ${ss}_{jl}$ as follows:

(4) \begin{equation*} {ss}_{jl}=-{\mathit{\log}}_{10}\left({ds}_{jl}\right) \end{equation*}

Finally, the specificity score for gene $l$ is obtained by the maximal score across all cell types,

(5) \begin{equation*} {ss}_l=\underset{j}{\max }s{s}_{jl} \end{equation*}

### Optimization model for cell type-specific marker identification

Our goal is to identify the smallest possible cell type-specific marker panel that can still accurately discriminate between the target cell types and background cell types. To achieve this, we propose a novel optimization model to maximize the cell type specificity score of selected genes, to minimize pairwise differences between the selected genes and all genes (i.e. to simultaneously maintain the hierarchical topology of the cell types) and to fix the number of selected genes to ensure the panel with desired small size. Balancing the two objectives with one constraint, we could formulate this problem as a mixed integer programming problem. By solving this problem, an optimal panel can be obtained and used to distinguish differentiate cell types.

Formally, we introduce an integer variable${w}_l$, which takes 1 for selecting gene $l$ and takes 0 for not selecting gene $l$. Then, the topology maintenance objective is constructed as follows. We first evaluate the difference between two cell types using the selected genes as follows:

(6) \begin{equation*} {d}_{ij}=\sum_{l=1}^n\ {w}_l{z}_{ij l} \end{equation*}

The sum of pairwise difference using the entire gene set could be

(7) \begin{equation*} D=\sum_{1\le i<j\le m}\sum_{l=1}^n{z}_{ijl} \end{equation*}

If the selected genes are good enough to maintain the topology of cell types, two terms should be simultaneously minimized, i.e. the total discrepancy $D-\sum_{1\le i<j\le m}{d}_{ij}$ and the pairwise discrepancy $\frac{1}{k}{d}_{ij}-\frac{1}{n}\sum_{l=1}^n{z}_{ij l}$. For the second-term pairwise discrepancy, a tolerant error ${\xi}_{ij}$ is introduced to minimize the difference between normalized distance of using selected genes $\frac{1}{k}{d}_{ij}$and using all genes $\frac{1}{n}\sum_{l=1}^n{z}_{ijl}$for the pair samples.

The specificity maximization objective is straightforward. The term $\sum_{l=1}^n{w}_l{ss}_l$ should be maximized to select the cell type specificity genes.

Therefore, an optimization model could be formulated,

(8) \begin{equation*} {\mathit{\min}}_{w_l,{\xi}_{ij}}\kern0.75em D-\sum_{1\le i<j\le m}{d}_{ij}+\lambda \sum_{1\le i<j\le m}{\xi}_{ij}-\mu \sum_{l=1}^n{w}_l{ss}_l \end{equation*}

Subject to

$$ \left\{\begin{array}{c}\begin{array}{@{}l}{d}_{ij}=\sum_{l=1}^n\ {w}_l{z}_{ij l}\\{}\sum_{l=1}^n{w}_l=k\\{}\frac{1}{k}{d}_{ij}+{\xi}_{ij}\ge \frac{1}{n}\sum_{l=1}^n{z}_{ij l}\end{array}\\{}{w}_l\in \left\{0,1\right\};{\xi}_{ij}\ge 0\end{array}\right. $$

The first term in the objective function $D-\sum_{1\le i<j\le m}{d}_{ij}$ is the total discrepancy between using selected genes and using the entire gene set. The second term in the objective function $\sum_{1\le i<j\le m}{\xi}_{ij}$ is the pairwise discrepancy between the selected genes and all genes. We minimize these two terms to maintain the topology (pairwise distance for samples) in a low dimension. The third term in the objective function $-\sum_{l=1}^n{w}_l{ss}_l$ is the negative sum of the specificity score of the selected genes, and we minimize it to select cell type-specific genes. Two parameters $\lambda$ and $\mu$ are introduced to balance the three objectives. This converts a multiple objective optimization problem to a single objective optimization. We notice that if $\mu =0$ the optimization model will be reduced to a dimensional reduction model to select features by dropping the prior information on the cell type specificity of the genes. Moreover, if $\mu =\lambda =0$, then the model will be converted to the multidimensional scaling (MDS) with feature selection.

The first constraint in model (8) is the distance between two cell types as defined in Equation (6). The second constraint is to fix the number of selected genes for biological interpretability and removing redundancy. The third constraint is to make the normalized cells’ pairwise distance by using selected genes as close as possible to the one using entire genes.

Taken together, we formulate an optimization model to identify the smallest, most cell type-specific marker panel while maintaining the hierarchical topology of the cell types. To solve the mixed integer linear programming problem, CPLEX software (IBM) was used. In practice, we can vary the parameter $k$ and solve our model for each $k$. After obtaining results for all $k$s, we can compare the model accuracies and find the best $k$ to determine our best panel. In addition, we test $\lambda =1,10,100,1000,10\ 000$ and $\mu =0,1,10,20,50,100,1000,10\ 000$ to select the parameters balancing the three terms in the objective function.

### Model evaluation for different criteria

To assess how well the cell types are separated by the selected gene set (marker panel), we propose three criteria to evaluate the model accuracy by counting the percentage of pairwise cell relationships can be correctly maintained with the selected panel.

Criterion 1 uses ${d}_{ij}$ to obtain the model accuracy,

(9) \begin{equation*} {C}_1=\frac{2}{m\left(m-1\right)}\sum_{1\le i<j\le m}I\left({d}_{ij}>T\right) \end{equation*}

where $I(x)$ is the indicator function and $I(x)=1$ when $x>0$ otherwise$I(x)=0$. *T* is a threshold to determine the cell type separation and set to 0.5 in our study.

Criterion 2 defines that pairwise cell is different if at least one gene’s fold change between two cells is larger than ${y}_0\big(0.5$ in our study). That is to say, ${z}_{ijl}$ is larger than 0.5 for gene $l$,

(10) \begin{equation*} {C}_2=\frac{2}{m\left(m-1\right)}\sum_{1\le i<j\le m}I\left(\#\left({z}_{ijl}>0.5;{w}_l=1\right)\right) \end{equation*}

where $\#(x)$ denotes the number satisfying the condition.

Criterion 3 uses pairwise cell is different if at least two genes’ fold change between two cells is larger than ${y}_0\ \big(0.5\ \mathrm{in}\ \mathrm{our}\ \mathrm{study}\big)$,

(11) \begin{equation*} {C}_3=\frac{2}{m\left(m-1\right)}\sum_{1\le i<j\le m}I\left(\#\left({z}_{ijl}>0.5;{w}_l=1\right)-1\right) \end{equation*}

### Network construction and module detection

To decipher the biological function for cell type-specific marker panel identified by MIMIC, we construct the cell type association network by using these selected markers. Specifically, we apply mutual information to measure the dependence between a pair of cell types [14]. The mutual information of cell type $i$ (${S}_i$) and cell type $j$ (${S}_j$) is calculated as follows:

(12) \begin{equation*} \mathrm{MI}\left({S}_i,{S}_j\right)=\sum_{x\in{S}_i}\sum_{y\in{S}_j}p\left(x,y\right)\log \left(\frac{p\left(x,y\right)}{p_1(x){p}_2(y)}\right) \end{equation*}

where $p\big(x,y\big)$is the joint probability of ${S}_i$ and ${S}_j$, and ${p}_1(x)$ and ${p}_2(y)$are the marginal probability of ${S}_i$and ${S}_j,$respectively. Here, ${S}_i$ only contains the markers identified by MIMIC. To compute the mutual information, we implement the fast calculation of pairwise mutual information method by using a Gaussian kernel estimator to estimate the distribution [15]. Then, we set a cutoff as 0.25 to get the cell type association network.

To reveal the structural information in the cell type association network, we apply Newman’s fast algorithm [16] to partition the network into several modules, which have dense connections between the nodes within modules, but sparse connections between nodes in different modules. In addition, the modularity measure $Q$ could be calculated to assess the module structure in the network. $Q=1$ indicates that the network has a perfect module structure and $Q=0$ means that there is no clear modular structure in the network. A higher value means better modularity [16].

### Extending MIMIC to a linear programming

To make MIMIC scale up to a high-dimensional dataset, we relaxed the integer variable in model (8) to allow an efficient algorithm. We have three objectives in MIMIC: (i) maintaining the cell hierarchical topology, (ii) selecting a minimal number of genes and (iii) maximizing the cell type specificity score for selected genes.

We first relax the integer decision variable ${w}_l\in \big\{0,1\big\}$ to a continuous variable ${w}_l\ge 0.$ But the decision variable ${w}_l\ge 0$ could not ensure ${\xi}_{ij}\ge 0$. Therefore, we let ${\xi}_{ij}={\xi}_{ij}^{+}-{\xi}_{ij}^{-}$, where ${\xi}_{ij}^{+},{\xi}_{ij}^{-}\ge 0$. Then, the first objective could be $\sum_{i,j}\big({\xi}_{ij}^{+}+{\xi}_{ij}^{-}\big)$. The second objective could be achieved by minimize $\sum_{l=1}^n{w}_l$. The third objective could be kept the same as MIMIC. Again, we can use two parameters, $\lambda$ and$\mu$, to convert the multiple objective optimization problem to be a single objective optimization problem. In this way, MIMIC could be extended to a linear programming problem as follows:

(13) \begin{equation*} {\min}_{w_l,{\xi}_{ij}^{+},{\xi}_{ij}^{-}}\sum_{i,j}{\xi}_{ij}^{+}+\sum_{i,j}{\xi}_{ij}^{-}+\lambda \sum_{l=1}^n{w}_l-\mu \sum_{l=1}^n{w}_l{ss}_l \end{equation*}

Subject to

$$ \left\{\begin{array}{@{}l}\sum_{l=1}^n{z}_{ij l}-\sum_{l=1}^n\ {w}_l{z}_{ij l}={\xi}_{ij}^{+}-{\xi}_{ij}^{-}\\{}{w}_l,{\xi}_{ij}^{+},{\xi}_{ij}^{-}\ge 0\end{array}\right. $$

If we let $\mu =0$, then it is also a dimensional reduction problem, which is the same as MIMIC. The parameter selection is the same as MIMIC.

### Datasets

We validated MIMIC using both expression profiles of SMs and TFs. Those expression profiles are measured by RNA-seq and are derived from the mouse ENCODE project [12]. Starting from the experimental matrix (https://genome.ucsc.edu/ENCODE/dataMatrix/encodeDataMatrixMouse.html), we selected 29 tissues with RNA-seq data generated from the UW lab, to avoid any bias introduced by data produced in other labs (Supplementary Table 1). Those tissues are from nine biological systems (muscular, circulatory, nervous, respiratory, digestive, excretory, endocrine, lymphatic and stem systems). The tree structure of the 29 tissues is shown in Supplementary Figure 1 available online at https://academic.oup.com/bib.

Then, we extracted the following subdatasets to validate MIMIC: (i) TFs’ expression profiles in 29 tissues, (ii) SMs’ expression profiles in 29 tissues and (iii) the combination of TFs and SMs’ expression profiles of 29 tissues. The TF list is from the AnimalTFDB database (http://www.bioguo.org/AnimalTFDB/) [17]. The SM list is from manual literature curation. The SMs and TFs used in this study are listed in Supplementary Tables 2 and 3 available online at https://academic.oup.com/bib. Additionally, we used a collection of RNA-seq data that contain 231 cell types or tissues [13]. Briefly, public RNA-seq data were uniformly reanalyzed using a RNA-seq pipeline adapted from Hutchins *et al*. [18]. The full list of samples is in Supplementary Table 4 available online at https://academic.oup.com/bib.

Finally, we used the DNase-seq data from mouse ENCODE project to validate MIMIC in high-dimensional data. We compiled a comprehensive enhancer annotation and quantified the openness/accessibility from DNase-seq data for 54 mouse cell types or tissues [19].

## Results

### MIMIC outperforms MDS

To demonstrate the performance gain of the mixed integer programming model in dimensional reduction, we compared the MDS technique with MIMIC without maximizing specificity (i.e. with parameter$\mu =0$). MDS is a method to visualize data points in a lower-dimensional manifold [20]. MDS used the dissimilarity matrix and maintained the pairwise distance in the lower-dimensional space. Here MDS was implemented by MATLAB R2018a.

**Figure 2:**
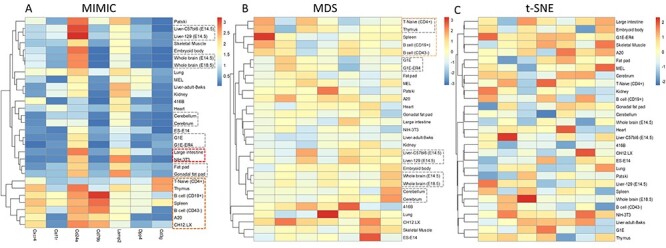
Clustered heatmaps of SMs identified by MIMIC (**A**), MDS (**B**) and t-SNE (**C**). MIMIC and MDS could identify similar cell types (gray rectangle). Moreover, MIMIC could find two endocrine cell types: gonadal fat pad and fat pad (bold red rectangle) but MDS failed. MIMIC further revealed a larger group of similar cell types (bold green rectangle). t-SNE failed to find a similar cell types.

We first applied MIMIC and MDS to the expression profiles containing 29 cell types with 43 SMs. MIMIC identified an SM-based panel consisting of Cd24a, Lamp2, Csf1r, Dpp4, Cxcr4, Cd3g and Cd79b ($\lambda =100$). To make the comparison fair with MIMIC, we checked MDS’s clustering results by selecting the same number of signatures (Figure 2A and B). Both methods found closely related cell type/tissue pairs: T-Naive (CD4+) and Thymus, Whole brain (E18.5) and Whole brain (E14.5), Liver-C57bl6 (E14.5) and Liver-129 (E14.5), G1E and G1E-ER4 (Gata1-erythriod cells), Cerebellum and Cerebrum. Overall, MIMIC could find more detailed subtypes than MDS. For example, MIMIC found endocrine cell types: Fat pat and Gonadal fat pad but MDS failed. Furthermore, MIMIC found a group of cells consists of A20 (B-lymphoma cell), CH12.LX (CH12 B cell lymphoma), B cell (CD43-), B cell (CD19+), Spleen, T-Naive (CD4+), Thymus. Importantly, all these cells belong to the lymphatic cell type, but MDS could only found a small cluster. This demonstrates that MIMIC could find a larger group of cell types having similar biological function than MDS. Regarding the biological interpretation, MIMIC could select specific sets of gene panels, rather than the broad signatures from MDS. This feature meant that MIMIC could identify the specific expression genes in a subgroup or tissue. For example, Cxcr4 was specifically expressed in lymphatic cells, similarly, Cd7b for B cells and Cd3g for T cells. In summary, MIMIC shows advantage over MDS in the identifying gene panels of SMs.

Then, we further demonstrated the performance gain of MIMIC in dimensional reduction using an extended expression profile dataset containing 29 tissues with 1345 TFs. MIMIC found a minimal TF panel consisting of the eight TFs: Basp1, Hopx, Nfib, Lbh, Myc, Zfp36, Uhrf1 and Satb1, with $\lambda =100$. Similarly, we compared the cluster results by MIMIC and MDS (see Supplementary Figure 2A and B available online at https://academic.oup.com/bib). MIMIC could identify more detailed subtypes than MDS. MDS found B cell (CD43−) and B cell (CD19+), Whole brain (E18.5) and Whole brain (E14.5), Liver-C57bl6 (E14.5) and Liver-129 (E14.5), G1E and G1E-ER4 (Gata1-erythriod cells), Fat pad and Gonadal fat pad. In addition to the above five subtypes, MIMIC could find the embryonic cell types: ES-E14 (embryonic stem cell line E14) and Embryoid body. Furthermore, MIMIC found similar cell types while MDS failed. For instance, large intestine and Liver-adult-8wks both belong to the digestive system and they were adjacent in clustering results from MIMIC. Similarly, two lymphatic cell types, CH12.LX and MEL, were clustered close together by MIMIC (see Supplementary Figure 2A available online at https://academic.oup.com/bib). In addition, MIMIC found a larger group of lymphatic cells: B cell (CD43−), B cell (CD19+), Spleen, T-Naive (CD4+), Thymus, MEL and CH12.LX, while MDS found only a partial one (see Supplementary Figure 2A and B available online at https://academic.oup.com/bib). In summary, MIMIC showed consistent advantages over MDS in dimensional reduction.

### MIMIC outperforms t-SNE in dimensionality reduction

t-SNE is a widely used technique in dimensionality reduction to visualize high-dimensional data in a two- or three-dimensional map [21]. t-SNE produces significantly better visualizations by reducing the tendency to crowd points together in the center of the map and shows good performance in creating a single map to reveal structure. We then compared the performance of MIMIC to t-SNE in dimensionality reduction.

In the 29 tissues, using 43 SMs dataset, MIMIC could cluster closely related cell types, while t-SNE failed (Figure 2C). For example, MIMIC could find T-Naive (CD4+) and Thymus, Whole brain (E18.5) and Whole brain (E14.5), Liver-C57bl6 (E14.5) and Liver-129 (E14.5), G1E and G1E-ER4 (Gata1-erythriod cells), Cerebellum and Cerebrum, Fat pat and Gonadal fat pad were all identified by MIMIC as closely clustered cell types, while t-SNE failed to cluster cell types close together. Moreover, MIMIC could find a large group of similar cell types, such as the lymphatic cells: B cells (CD43−), B cells (CD19+), Spleen, T-Naive (CD4+), Thymus, MEL and CH12.LX, while t-SNE failed. Similar results could also be obtained when using the TF dataset (see Supplementary Figure 2C available online at https://academic.oup.com/bib). In summary, MIMIC showed consistently better performance in dimensionality reduction.

### MIMIC outperforms a standard single gene approach to identify core genes

To further demonstrate the performance gain of MIMIC in identifying cell type-specific genes, we compared MIMIC with a standard single gene-based approach to identify candidate core TFs for cell sorting [22]. This approach evaluated the specificity score of each gene in a query cell type by calculating the difference between the observed and ideal patterns, and the difference was assessed by the entropy-based measure of JSD. Here the specificity score of gene$l$in tissue$j$was calculated as in Equation (3) by using the third quantile of the expression level of all the tissues as the background.

We applied MIMIC and the standard approach to the expression profiles of 29 cell types with 43 SMs. MIMIC identified a cell type-specific gene panel consisting of eight SMs: Cd24a, Lamp2, Cxcr4, Cd79b, Cd19, Rhag, Cd3g and Csf1r ($\lambda =100, \mu =10$) (Supplementary Table 5). To make a fair comparison with MIMIC, we selected the same number of SMs using a standard (simple) approach (see Supplementary Figure 3 available online at https://academic.oup.com/bib). Specifically, we collected the best SMs in all cell types then selected the top eight in each cell type as the final markers. Finally, the standard approach could identify Cd19, Cd79b, Mme, Lamp3, Il18rap, Cd177, Cd3g and Rhag. Both MIMIC and the standard approach identified four cell type-specific genes: Cd79b, Cd19, Rhag and Cd3g, which support MIMICs’ ability to select cell type-specific genes. Moreover, MIMIC obtained a model accuracy as 0.99 (Criterion 1) to distinguish pairwise cell types, which was much better than using the top eight genes by the standard approach. Furthermore, unlike the standard approach identified the cell type-specific SMs from single cell type, MIMIC tended to select combinations of SMs that distinguish different cell types. For example, Il18rap was a cell type-specific marker for 416B cells, as identified by the standard approach, MIMIC however demonstrated that the combination of high expression of Cxcr4, and the low expression of Cd79b, Cd24a and Cd3g were specific for 416B cells (Figure 2A). In addition, MIMIC showed advantages in distinguishing closely related cell types. Both MIMIC and the standard approach found that Cd3g is highly expressed in T-Naïve (CD4+) and Thymus cell types, but only MIMIC could identify that Cd24a that is specifically expressed in the Thymus. Like the standard approach, MIMIC showed performance gains in maintaining the hierarchical topology of different tissues. MIMIC could identify the endocrine cell Fat pad and Gonadal fat pad, lymphatic cell B cell (CD19+), Spleen, B cell (CD43+), A20, CH12.LX, T-Naïve (CD4+) and Thymus, Liver-C57bl6 (E14.5) and Liver-129 (E14.5), Whole brain (E14.5) and Whole brain (E18.5), etc. (Supplementary Figure 3). In summary, MIMIC identified a cell type-specific SM panel that balances the cell type specificity and hierarchical topology and can reveal SM combinations that better distinguish cell types.

**Figure 3:**
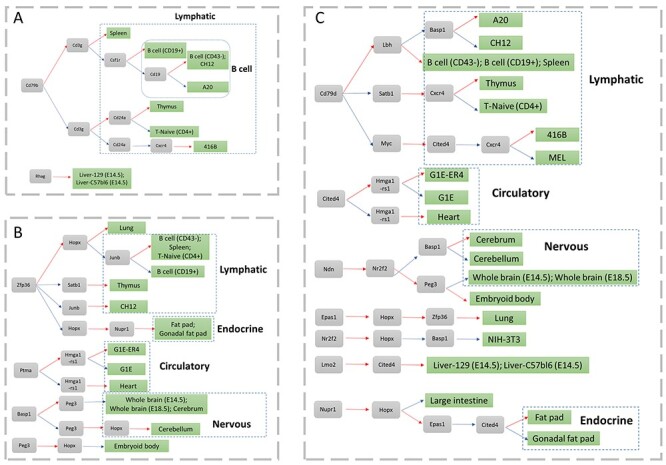
Cell type-specific markers for different cell types. The gray and green boxes denote gene and cell, respectively. The red and blue arc line denoted high and low expression. The blue dash line box denoted a cell type group. (**A**) Cell type-specific SMs, (**B**) cell type-specific TFs and (**C**) cell type-specific markers by combining TFs and SMs.

We next demonstrated the advantages of MIMIC in cell type specificity in the TF expression dataset. MIMIC selected a cell type-specific TF panel consisting of Ptma, Nupr1, Hopx, Basp1, Hmga1-rs1, Zfp36, Satb1, Peg3 and Junb with ($\lambda =100,\mu =100$) (Supplementary Table 6). The standard approach using gene expression quartiles could find Fhl2, Gata2, Nkx6-2, Nupr1, Pou5f1, Prrx1, Sox11, Tcf7 and Zic1. Then, we compared the two methods using a cluster analysis result (see Supplementary Figure 4 available online at https://academic.oup.com/bib). Overall, MIMIC showed advantages in identifying TF combinations for distinguishing different cell types. For example, the standard approach found that Fhl2 was expressed specifically in heart. While MIMIC found that the combination of highly expressed Hopx and Hmga1-rs1 was specific for heart (Figure 3B). Conversely, highly expressed Hmga1-rs1 and lowly expressed Hopx were specific for G1E-ER4 cell type. Considering the fact that Ptma was highly expressed in G1E-ER4 and G1E, then the combination of Ptma1 and Hmga1-rs1 was sufficient to distinguish the similar cell types G1E-ER4 and G1E. Furthermore, MIMIC showed advantages in maintaining the hierarchical topology compared to the standard approach. For example, our model could maintain the close relationship between Fat pad and Gonadal fat pad, B cells (CD43-), B cells (CD19+), and Spleen, while the standard approach failed to preserve cell type topology (Figure 3B). In summary, MIMIC showed improved performance in identifying TF combinations that distinguish different cell types, compared to the standard approach, and MIMIC was more capable at balancing the hierarchical topology among different cell types.

### MIMIC identifies biologically meaningful cell type-specific genes

Next, we demonstrated that mixed integer programming could identify biologically meaningful genes. We validated the specific gene panel consisting of Cd24a, Lamp2, Cxcr4, Cd79b, Cd19, Rhag, Cd3g and Csf1r in the SMs’ dataset. The alpha-chemokine receptor Cxcr4 was expressed throughout B cell ontogeny, and the B cells generated in the bone marrow migrate into the spleen [23]. This was consistent with our study that Cxcr4 was highly expressed in two types of B cells (CD43−) and B cell (CD19+), two types of B cell lymphoma A20 and CH12.LX, and Spleen. However, Cxcr4 was upregulated in other cells such as Lung, Thymus, 416B and T-Naïve (CD4+) cells. Cd79b might help to distinguish B cells from these cells. Cd79b, a gene encodes a cell surface immunoglobulin beta, was necessary to distinguish B cells. A recent study showed that Cd79b was specific for B cells rather than T cells [24, 25]. In our data, Cd79b was downregulated in these cells. Therefore, Cd79b might be a specific marker for B cells, and Cd79b and Cxcr4 might function as markers for Lung, Thymus, 416B cells and T-naïve (CD4+) cells. Moreover, Cd3g is closely related to the surface protein CD3 in T cells and a deficiency of Cd3g leads to immunodeficiency [26, 27]. Indeed, Cd3g is a specific biomarker for Spleen, T-Naive (CD4+) and Thymus. Considering Cd79b was a specific biomarker for B cells and spleen, then Cd3g and Cd79b were potentially specific biomarker combinations for T cells. Simultaneously, Cd24 could distinguish T-Naïve (CD4+) and the Thymus well. In summary, the multi-biomarker panel has a plausible biological interpretation.

MIMIC obtained similarly biologically meaningful results in TF dataset. It revealed a multi-marker panel consisting of Ptma, Nupr1, Hopx, Basp1, Hmga1-rs1, Zfp36, Satb1, Peg3 and Junb ($\lambda =100,\mu =100$). Satb1 is a matrix attachment regions (MAR) binding protein, and it orchestrates temporal and spatial expression of multiple genes during T-cell development [28, 29]. This was consistent with our finding that Satb1 is highly expressed in T-Naive (CD4+) and Thymus cell. Similarly, Zfp36 was specifically expressed in T-Naive (CD4+) cells [30] and MIMIC correctly identified Zfp36 as a discriminant for T-Naive (CD4+) cells and the Thymus. Moreover, Zfp36 is a component of a negative feedback loop that interferes with inflammatory factors like TNF-alpha and IL-10, and mice deficient for Zfp36 develop chronic inflammatory diseases [31]. Considering that inflammation is closely related to immune cell function, Zfp36 was also highly expressed in B cell subtypes and the Spleen. This was supported in our study, as Zfp36 and Satb1 are a specific biomarker combination for B cells and Spleen. A20 and CH12.LX are tumor cell lines of presumed B cell origin and the two cell lines appear to have low expression of Zfp36. A study showed that A20 inhibited TNF-mediated apoptosis by inhibiting NF-kB, but it could not decrease the expression of the pro-oncogene Junb. In our study, Junb was highly expressed in A20 cells and could be used as a biomarker to distinguish A20 and CH12.LX cells. Hopx is also a tumor suppressor gene, and studies showed that the decreased Hopx may lead to the progression of tumor [32], which was consistent with the fact that Hopx is lowly expressed in CH12.LX and A20 cells. Furthermore, Hopx may interact with serum response factor (SRF) and modulate SRF-dependent cardiac-specific gene expression and cardiac development; hence it was a good marker for heart. These results suggest that the cell type-specific TF panel could be potentially combined to assist cell sorting.

**Figure 4:**
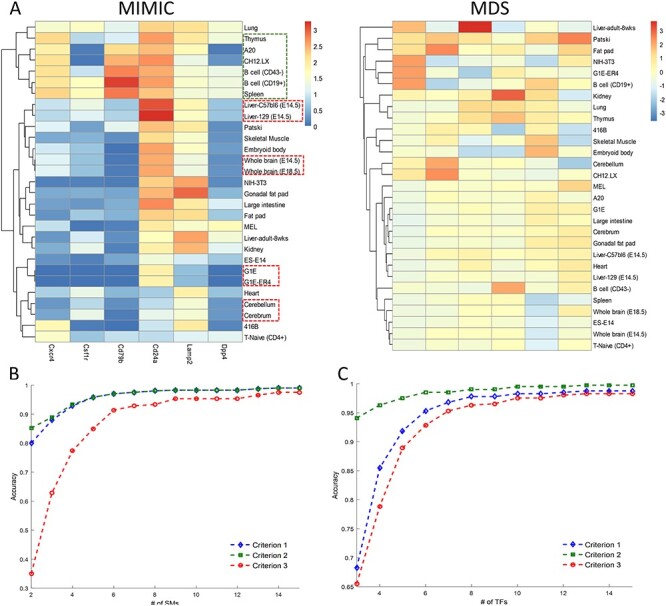
(**A**) Comparison between MIMIC and MDS on the robustness against possible batch effects in the data. Before batch effect removal processing, MIMIC found similar cell types (bold red/green rectangle) but MDS failed. Comparisons of different accuracy evaluation criteria of MIMIC for SMs (**B**) and TFs (**C**). They show that different accuracies gave close results when selecting more than nine markers.

**Figure 5:**
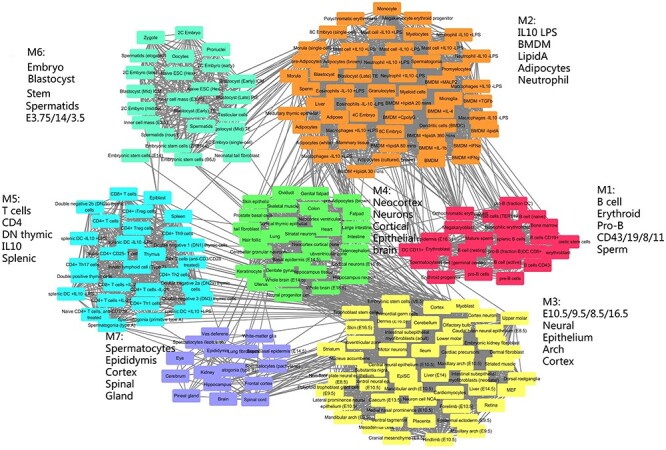
The cell type association network constructed by the TF marker panel selected by MIMIC. Nodes were the cell types and edges are the similarity between cell types. Every module was labeled by the top five most frequent annotations. In total, there were seven modules M1 through M7. Module M5 was abundant with T cell types, module M6 was most abundant with embryonic cells and M1 was most abundant with B cell types.

### The performance gain of MIMIC is robust

To demonstrate that MIMIC was robust to possible batch effects in expression data (see Supplementary Figure 5 available online at https://academic.oup.com/bib), we compared the clustering analysis by MIMIC and MDS in pre-processing data (without batch effect removal) and post-processing data (with batch effect removed by quantile normalization) for SMs. MIMIC identified a panel of SMs consisting of Cd24a, Lamp2, Csf1r, Dpp4, Cxcr4 and Cd79b (Figure 4A). We also selected the same number of signatures by MDS. All the SMs identified by MIMIC were the same as found in the post-processed data (Figure 2A), except Cd3g. Therefore, MIMIC showed little difference between pre-processing and post-processing data. For example, our model correctly predicted the topological relationship between G1E and G1E-ER4, Cerebellum and Cerebrum, Liver-C57bl6 (E14.5) and Liver-129 (E14.5), Whole brain (E18.5) and Whole brain (E14.5), along with a further group of cell types: Thymus, A20, Ch12.LX, B cell (CD43-), B cell (CD19+) and Spleen. In contrast to MIMIC, MDS performance was unstable when considering after processing data. After processing, MDS could preserve the topology of closely related cell types: B cell (CD43-) and B cell (CD19+), Whole brain (E18.5) and Whole brain (E14.5), Liver-C57bl6 (E14.5) and Liver-129 (E14.5), G1E and G1E-ER4, Cerebellum and Cerebrum (Figure 2B). However, these topological relationships disappear in pre-processed data (Figure 4A).

Next, we demonstrated the robustness of MIMIC in different criteria in Equations (9–11) and thoroughly assessed how closely the selected maker panel can maintain cell type topology. We used all the criteria to evaluate MIMIC for SMs’ and TFs’ dataset. In SMs’ dataset, criterion 1 was similar to criterion 2, and with the number of selected SMs up to 9, the three criteria became almost identical results (Figure 4B). Similar results were obtained when using the extended TF expression profile (Figure 4C). Thus, MIMIC was robust in selecting the number of markers, with different evaluation criteria.

**Figure 6:**
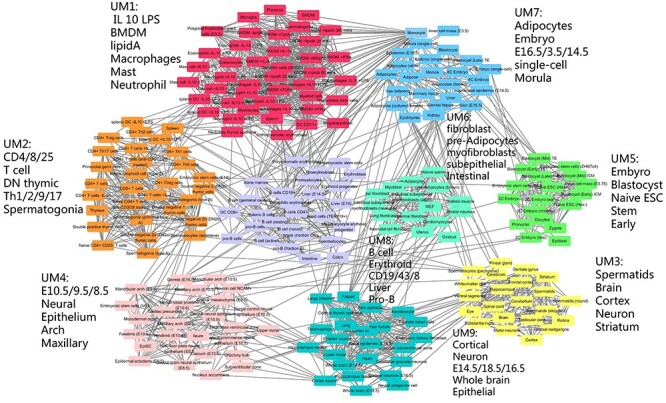
The cell type similarity network constructed by the TF and SM combination panel revealed by MIMIC. In total, there were nine modules: UM1 through UM9. UM5 was abundant with embryonic cell types, UM6 was abundant with fibroblastic cell types and UM8 was abundant with B cells.

**Figure 7:**
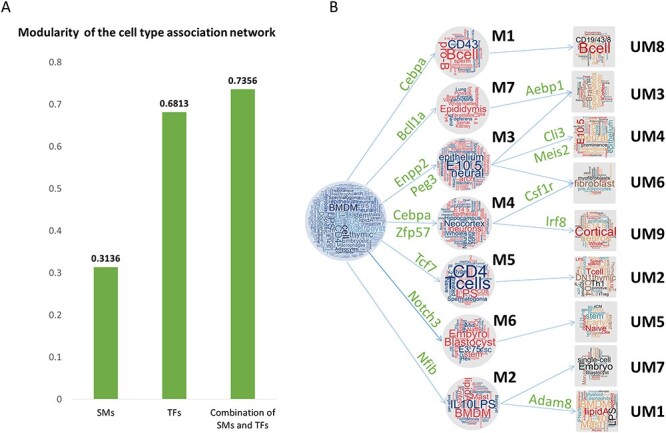
Relationship between SM, TF and SM&TF modules. (**A**) Modularity of the cell type association network using SMs only, TFs only and combining SMs and TFs. The combination of SMs and TFs showed the best ability to reveal cell type organization. (**B**) The relationship between seven TF-derived modules (M1 through M7) and nine SM&TF-derived modules (UM1 through UM9). The two SMs, Csf1r and Adan8, help to divide M2 into UM7 and UM1 and to divide M4 into UM6 and UM9, respectively.

### Combining TFs and SMs further decipher cell type specificity

Since TFs and SMs have performed well in cell sorting individually, we combined the TFs and SMs and expected to obtain better performance. MIMIC identified a cell type-specific biomarker panel, consisting of two SMs (Cxcr4 and Cd79b) and 16 TFs (Lbh, Hopx, Basp1, Peg3, Est1, Epas1, Myc, Zfp36, Hmga1-rs1, Lmo2, Nfib, Nr2f2, Ndn, Nupr1, Satb1 and Cited4) with ($\lambda =100,\mu =100$) (see Supplementary Figure 6 available online at https://academic.oup.com/bib). Furthermore, we selected a cutoff of 1.3 for the specificity score for each TF or SM, then we assigned the markers to each cell type (Supplementary Table 7). According to this, we found that the SM and TF optimal panel could distinguish more cell types than simply pooling the cell type-specific TFs and SMs together (Figure 3C). Firstly, it performed better in distinguishing lymphatic cell from others and further obtained good performance in inter-lymphatic cell types (Figure 3). Similar results were also found in nervous system cells. In addition, MIMIC not only found cell type-specific biomarkers for endocrine cells but also found the marker Cited4 that can distinguish between Fat pad from Gonadal fat pad. In conclusion, we could generate a panel of cell type-specific biomarkers by combining SMs and TFs and so achieve better performance for cell sorting.

### MIMIC reveals cell type modules associated with makers in mouse

To further demonstrate the usage of MIMIC to reveal the associations among cell types, we applied it to a larger dataset containing 231 cell types [13]. MIMIC identified an optimal panel of SMs consisting of Cxcr4, Adam8, Dpp4, Csf1r, Cd22, Cd24a, Cd180, Cd247 and Cd79b in SMs’ expression profiles with $\lambda =100,\mu =10$ (Supplementary Table 8). Cd79b is a cell type-specific marker for B cells, which is consistent with our findings in the ENCODE dataset. We then constructed a cell type association network using only this selected SM panel and detected the resulting modules. We identified five modules with the modularity function $Q$ = 0.3136, which indicated the network has a certain modularity property. We could find a module enriched with B cell types and their cell type-specific markers: Cd79b and Dpp4. Furthermore, we found that Cd22 and Cd79b can distinguish many B cell types from other cell types. There was also another module enriched with T cell types, and Cd247 was a specific biomarker for this module.

Furthermore, we used the expression data for 1345 TFs and found a cell type-specific TF panel consisting of Cebpa, Nfib, Bcl11a, Zfp57, Enpp2, Peg3, Tcf7 and Notch3 with$\lambda =100,\mu =100$ (Supplementary Table 9). Network construction and module detection for this panel gave seven modules with modularity function *Q* = 0.6813 (Figure 5), which was larger than the SM-derived network. This indicates that the TFs were more tissue specific than SMs, and so TFs could better distinguish cell types. The 231 cell types were organized into seven modules (M1, M2, …, M7 as labeled in Figure 5) and every module was annotated with the top five cell types with the highest frequency (Figure 5). For example, module M5 was enriched with T cells with their specific marker Tcf7, which was consistent with our study in ENCODE 29 tissues. Moreover, more than half of the cell types in M6 highly expressed Zfp57, and most of these cell types were embryonic cell types. In addition, Enpp2 is highly expressed in nearly all cell types in module M7. In summary, MIMIC showed good performance in identifying biologically meaningful modules and their cell type-specific biomarkers in a larger dataset.

Finally, we combined TFs and SMs together to sort all the cell types and then study the cell type organizations using our selected panels of TFs and SMs. We expected to find more functional modules and their module-specific markers. As a result, MIMIC integrated TF and SM expression profiles and found 14 cell type-specific markers consisting of 2 SMs, Adam8 and Csf1r, and 12 TFs, Aebp1, Ank2, Bcl11a, Cebpa, Enpp2, Gli3, Irf8, Meis2, Nfib, Notch3, Tcf7 and Zfp57 ($\lambda =100,\mu =100$) (Supplementary Table 10). Module analysis identified nine modules with modularity function *Q* = 0.7356 (UM1, through UM9 in Figures 6 and 7A). This increased modularity score demonstrated that the combination of TFs and SMs can further improve the cell type discrimination ability (Figure 7A). The modules obtained by combining TFs and SMs (Figure 6) were largely consistent with using TFs alone (Figure 5). In order to check the detailed similarities and differences, we mapped the relationships between the two modules (Figure 7B). For example, we found that T cells are enriched in module UM2 and by the cell type-specific marker Tcf7. This corresponded to module M5. The brain-associated module UM3 with their markers Ank2 and Enpp2 was partially merged as parts of module M7 and M3 (Figure 7B). The differences were also distinct. Expression of Gli3, Meis2 and Zfp57 discriminated cell types enriched in the epithelium cell module UM4, and Aebp1 was highly expressed in cell types in the fibroblast module UM6. Moreover, Zfp57 was specifically highly expressed in the embryonic module UM7, and Nf1b was differentially expressed in cell types enriched in the neuron module UM9. Specifically, the two SMs, Adam8 and Csf1r, helped TFs to further reveal more detailed cell type organization. Adam1 helped to divide M2 into two submodules. One was module UM1 (marked by Adam8), which was correlated with immune cell types. The other was UM7, which was enriched in embryonic cell types. Csf1r helped to divide module M4 into two submodules: UM6 and UM9 (Figure 7B). UM4 is discriminated by Csf1r and is enriched in fibroblast. While UM9 was labeled by another TF Irf8 with cortical cell types enriched. In summary, MIMIC could decipher more specific modules than SMs and TFs individually and also showed module specific markers.

### MIMIC shows scalability to select cell type-specific enhancer markers

To demonstrate MIMIC’s scalability in higher dimensions, we extended MIMIC to a linear model and applied it to an enhancer accessibility dataset (see Dataset section for details). Potentially, MIMIC could select the minimal set of discriminatory enhancers required for a cell type, and so help prioritize critical enhancers for further study. MIMIC selected 10 enhancers: chrX:84482600-84484600, chr4:947138000-94715800, chr10:119857900-119859900, chr3:127632400-127634400, chr1:71144700-71146700, chr9:40879000-40881000, chr12:53706750-53708750, chr3:101969500-101971500, chr7:80704850-80706850, chr3:93354000-93356000 with $\mu =1,\lambda =887$. GREAT enrichment analysis demonstrated that these enhancers showed significance in the MSigDB immunologic signature ‘GSE22886_IL2_VS_IL15_STIM_NKCELL_UP’ with adjusted *P*-value $4.56\times{10}^{-2},$ and the signature was enriched by immune cell-specific expression gene pattern [33]. More interestingly, the enhancer chr4:947138000-94715800, locating at the nearest gene Tek in immunologic signature, was highly expressed in the lymphatic cell type and blood cell type, which could be a cell type-specific marker for the two cell types. Tek encodes a cell-surface receptor that belongs to the protein tyrosine kinase Tie2 family and regulates angiogenesis and maintenance of vascular quiescence. It has anti-inflammatory effects by preventing the leakage of proinflammatory plasma proteins and leukocytes from blood vessels. This was consistent with our study. Furthermore, cluster analysis for these genes also found an immune cell group containing A20, CH12.LX, Spleen, B-cell_(CD19+), TReg-Activated B-cell_(CD43−), T-Naive, THelper-Activated, TReg and blood association cell group Erythroblast_2, MEL, MEL−GATA−1−ER, Erythroblast_1. Additionally, clustering identified an embryonic cell group containing: ZhBTc4_1, ZhBTc4_2, ES − CJ7, ES − E14, ES−WW6_1, ES−WW6_2; embryo 11.5 days tissue group: HindlimbBud, Mesoderm, ForelimbBud, HeadlessEmbryo; along with a smaller embryonic cell group containing: Liver_(14.5-day)_1, G1E−ER4, G1E, EPC_(CD117−_CD71+_TER119+), Liver_(14.5-day)_2, EPC_(CD117+_CD71+_TER119−), EPC_(CD117+_CD71+_TER119+).

We next compared MIMIC with t-SNE. MIMIC could find a large lymphatic cell group A20, CH12.LX, Spleen, B − cell_(CD19+), TReg-Activated B − cell_(CD43−), T − Naive, THelper-Activated, TReg while t-SNE found these cell types in two cell groups. In addition, both MIMIC and t-SNE found four cell groups, which demonstrated that MIMIC had good performance and maintained hierarchical topology among different cell types. Although t-SNE could find the nervous cell group Cerebellum, Cerebrum, WholeBrain_(8-week), WholeBrain_(14.5-day), WholeBrain_(18.5-day), Retina_(1-day), Retina_(7-day), Retina_(8-week), MIMIC found these cell types located near to each other in cluster analysis. Nevertheless, t-SNE could find an endocrine cell group containing: FatPad, GenitalFatPad, while MIMIC failed to cluster this group. This situation may be due to the need for MIMIC to tune the balance between maintaining hierarchical topology among different cell types and selecting cell type-specific markers. Most importantly, MIMIC directly selected enhancers, while t-SNE selected enhancer signatures, so that we could obtain the biological meaning of selected enhancers by MIMIC.

Overall, comparing MIMICs analysis of both TFs and SMs, our results show that enhancer accessibility is cell type-specific and could further assist in identifying cell type-specific critical enhancers and accurately discriminating cell types for cell sorting. For example, it was hard to distinguish WholeBrain_(14.5-day) from WholeBrain_(18.5-day) samples by combining TFs and SMs, but the enhancer chr3:93354000-93356000 was more open in WholeBrain_(18.5-day) compared to WholeBrain_(14.5-day). Moreover, it was closed in the WholeBrain_(8-week), aiding cell type discrimination. We studied the closest genes, Rptn and Hrnr, to this enhancer, and Gene Ontology suggested that both genes are related to calcium ion binding. Especially for Rptn, it was related to a ‘Developmental Biology pathway’. Potentially this was the reason that the enhancer is open only in the median stage (WholeBrain (18.5-day)) rather than in the early (14.5-day) or late (8-week) stages. Besides that, enhancer data could provide other evidence to enhance the power to select cell type-specific genes/markers for different cell types. For example, Cd79b was a specific marker for B-cell type group, and we found that the enhancers chr1:71144700-71146700 and chr4:94713800-94715800 were more open in the cell group. In addition, the Epas1 was highly expressed in the lung and was lowly expressed in the NIH3T3 cells; here we found that the enhancers chr9:40879000-40881000 and chr3:127632400-127634400 were more open in the lung.

## Discussion

Identifying cell type-specific markers is a challenging task because of the large amount of cell types and their complicated hierarchically organized structure. Many approaches have identified cell type-specific markers by focusing on single marker analysis in each tissue, and no systematic methods were developed to identify non-redundant markers for all cell types in an integrative way. In addition, the relationships among different tissues were not considered. We thus developed an optimization method (MIMIC) to identify minimal sets of cell type-specific markers that simultaneously maintain the hierarchical topology among different tissues. Specifically, the optimization method optimizes the pairwise difference between selected markers and all markers to maintain hierarchical topology and maximize the sum of the specificity score of selected markers. Moreover, the model allows us to vary the number of selected markers and obtain an optimal marker panel with better model accuracy by balancing the number of selected markers.

We demonstrated the performance gain of MIMIC not only on expression profiles of SMs but also on an extended dataset of expression profiles of TFs. The cell type-specific panel composed of both TFs and SMs identified by MIMIC showed plausible biological meaning, by discriminating different cell types. Furthermore, MIMIC could find close relationships among different tissues, especially for subtypes of a cell type, such as B cell (CD19+) and B cell (CD43-), Whole brain (E14.5) and Whole brain (E18.5), G1E and G1E-ER4, Liver-C57bl6 (E14.5) and Liver-129 (E14.5). Moreover, MIMIC could find a close relationship between very similar cell types, such as gonadal fat pad and fat pad cells, whole brain and cerebrum. In addition, MIMIC showed robust performance under varying parameters, and the data pre-processing steps. Hence, MIMIC showed advantages in identifying cell type-specific marker panels for assisted cell sorting and cell type discrimination.

Our improved model is, however, limited in several ways. MIMIC is developed to select a number of proteins to assist in cell sorting, but it uses only the mRNA level, which may not reflect protein concentrations. Indeed, Jiang *et al*. reported that the Spearman correlation between RNA and protein abundance is only ~0.46 across 32 normal human tissues [34]. Using the same dataset, MIMIC select the totally different gene set in RNA dataset comparing with selected genes in protein dataset. However, the selected RNAs have better correlation (median correlation = 0.75) with proteins (Supplementary Figure 7). In method development, firstly, the evaluation of difference between different tissues was calculated by the L_1_ norm may be further extended. Maybe high or low expression level is more suitable for distinguishing different tissues. Secondly, MIMIC is limited in model construction. We only considered a marker’s specificity for the maximal score in one tissue, which may thus allow redundant marker selection. Thirdly, MIMIC only considers the pairwise distance, which leads to computational inefficiency when the sample size increases, for example in the thousands or tens of thousands of cells now seen in typical single cell RNA-seq (scRNA-seq) datasets). We tested MIMIC on a small scRNA-seq dataset (124 single cells) [35] and promising performance was shown (see Supplementary Figure 8 available online at https://academic.oup.com/bib). However, the current implementation of MIMIC makes it impractical to apply to larger scRNA-seq datasets, such as an expression profile for >1000 single cells. Finally, MIMIC integrates TFs and SMs by simply combining the two data matrices into a single matrix without considering the relationship between them. The same situation would happen if we integrate TFs, SMs and enhancers. Maybe we could add gene regulatory network information to integrate the three datasets [36]. Similarly, our minimal sets of marker that discriminate cell types contain nine markers, which remains challenging to experimentally perform on a FACS machine.

In conclusion, we constructed a mixed integer programming model, MIMIC, to identify minimal optimal cell type-specific marker panels that simultaneously maintain the hierarchical topology among different cell types to assist cell sorting. Our contribution is to broaden the two terms together by optimizing three objectives: the sum of the pairwise differences, scaled pairwise differences and the sum of markers’ specificity score. Simultaneously, we fixed the number of selected markers and obtained an optimal panel with good accuracy and a relatively small number of selected features. We validated the MIMIC panel in two datasets using expression profiles of SMs and TFs. Our results demonstrated that MIMIC showed advantages in dimensionality reduction when compared to MDS and in cell type-specific marker identification than a standard approach. We also demonstrated the application of MIMIC in an extended dataset and note that our new method can be widely extended to other datasets for cell sorting.

**Key Points**

- We develop MIMIC as a useful tool to select an optimal gene panel to maintain the hierarchical topology among different cell types and simultaneously maximize the specificity.
- MIMIC outperforms MDS and t-SNE in dimensional reduction and cell type clustering and reveals biological meaningful marker combinations.
- MIMIC identifies a panel of TFs and SMs on a large collection of 641 RNA-seq samples covering 231 cell types and reveals the modularity of the cell type association network
- MIMIC could be extended to select enhancer markers on mouse ENCODE data.

## Supplementary Material

Supplementary_Table_1-4_bbab235

Supplementary_Table_5-10_bbab235

## Notes

**Meng Zou** is an instructor at the Department of Mathematics, Huazhong University of Science and Technology.

**Zhana Duren** is an assistant professor at the Department of Genetics and Biochemistry, Clemson University.

**Qiuyue Yuan** is a PhD candidate at the Academy of Mathematics and Systems Science, CAS.

**Henry Li** is a postdoc at the Department of Health Research & Policy, Bio-X Program Stanford University.

**Andrew Paul Hutchins** is an associate professor at the Southern University of Science and Technology.

**Wing Hung Wong** is a professor at the Department of Statistics, Department of Biomedical Data Science, Bio-X Program Stanford University.

**Yong Wang** is a professor at the CEMS, NCMIS, MDIS, Academy of Mathematics and Systems Science, Center for Excellence in Animal Evolution and Genetics, University of Chinese Academy of Sciences, CAS.
