# Supplementary material for: MIMIC: an optimization method to identify cell type-specific marker panel for cell sorting
Source: Brief Bioinform. 2021 Jun 26;22(6):bbab235. doi: 10.1093/bib/bbab235 (PMC8575015; doi:10.1093/bib/bbab235)
Supplement: Supplementary_Table_5-10_bbab235 [file supplementary_table_5-10_bbab235.docx]

| **Supplementary Table 5.** Specificity score for each surface marker selected by MIMIC in 29 cell types | | | | | | | | |
| --- | --- | --- | --- | --- | --- | --- | --- | --- |
| Cell type | Cxcr4 | Cd24a | Cd79b | Csf1r | Cd19 | Rhag | Lamp2 | Cd3g |
| Skeletal Muscle | 0.1309 | 0.2397 | 0.1887 | 0.2633 | 0.3102 | 0.1264 | 0.2493 | 0.4077 |
| G1E | 0.0590 | 0.1804 | 0.1717 | 0.1167 | 0.2111 | 0.7744 | 0.0955 | 0.4646 |
| G1E-ER4 | 0.0436 | 0.1672 | 0.1023 | 0.1381 | 0.2114 | 0.7834 | 0.0947 | 0.5596 |
| Heart | 0.1803 | 0.0222 | 0.2450 | 0.6829 | 0.2986 | 0.1084 | 0.4369 | 0.3852 |
| Cerebellum | 0.0595 | 0.0225 | 0.1262 | 0.2846 | 0.2407 | 0.1160 | 0.2166 | 0.3101 |
| Cerebrum | 0.0652 | 0.0538 | 0.1068 | 0.6154 | 0.2106 | 0.1199 | 0.2216 | 0.3199 |
| Whole brain (E14.5) | 0.2598 | 0.5023 | 0.1058 | 0.1723 | 0.2088 | 0.1224 | 0.1715 | 0.2983 |
| Whole brain (E18.5) | 0.2733 | 0.5159 | 0.1076 | 0.1694 | 0.2100 | 0.1130 | 0.1720 | 0.2930 |
| Lung | 0.4477 | 0.2816 | 0.6259 | 0.8162 | 0.5841 | 0.1110 | 0.5627 | 0.4957 |
| NIH-3T3 | 0.0569 | 0.2811 | 0.1227 | 0.1129 | 0.2298 | 0.1901 | 0.9918 | 0.3336 |
| Large intestine | 0.0703 | 0.5608 | 0.1842 | 0.1233 | 0.2794 | 0.1217 | 0.3983 | 0.4408 |
| Liver-adult-8wks | 0.0751 | 0.1067 | 0.1492 | 0.5156 | 0.2377 | 0.5884 | 0.7551 | 0.3623 |
| Liver-C57bl6 (E14.5) | 0.1809 | 0.7555 | 0.2568 | 0.5415 | 0.2415 | 1.5771 | 0.2708 | 0.3008 |
| Liver-129 (E14.5) | 0.1795 | 0.9100 | 0.3343 | 0.4634 | 0.3577 | 1.5513 | 0.2195 | 0.4121 |
| Kidney | 0.0798 | 0.1387 | 0.1175 | 0.5576 | 0.2596 | 0.1201 | 0.7137 | 0.3360 |
| Patski | 0.3680 | 0.5999 | 0.8599 | 0.1712 | 0.6847 | 0.2006 | 0.7164 | 0.3205 |
| Fat pad | 0.0663 | 0.2641 | 0.2034 | 0.2416 | 0.2088 | 0.9843 | 0.5472 | 0.5904 |
| Gonadal fat pad | 0.1184 | 0.3159 | 0.2461 | 0.2311 | 0.3156 | 0.1420 | 0.9428 | 0.4654 |
| 416B | 0.6033 | 0.0245 | 0.1079 | 0.1044 | 0.2094 | 0.1468 | 0.3687 | 0.3346 |
| A20 | 0.7376 | 0.3234 | 1.4781 | 0.1148 | 0.9992 | 0.1852 | 0.2500 | 0.3636 |
| B cell (CD19+) | 0.9556 | 0.4420 | 2.4885 | 0.8611 | 2.2271 | 0.2732 | 0.2216 | 0.8847 |
| B cell (CD43-) | 1.0614 | 0.3905 | 2.0758 | 0.3706 | 2.3770 | 0.1750 | 0.2236 | 0.3767 |
| CH12.LX | 1.0004 | 0.5023 | 1.8407 | 0.1272 | 1.5063 | 0.1089 | 0.1559 | 0.8355 |
| MEL | 0.1832 | 0.1435 | 0.1515 | 0.1014 | 0.2099 | 0.8192 | 0.1815 | 0.4414 |
| Spleen | 1.2144 | 0.3564 | 2.2090 | 0.9885 | 2.1800 | 0.2029 | 0.2128 | 1.7496 |
| T-Naïve (CD4+) | 0.4700 | 0.0227 | 0.3788 | 0.2454 | 0.4585 | 0.1497 | 0.1015 | 2.4958 |
| Thymus | 1.1967 | 0.5807 | 0.5155 | 0.3005 | 0.4781 | 0.1178 | 0.5363 | 2.4521 |
| ES-E14 | 0.0702 | 0.0911 | 0.2020 | 0.1032 | 0.2185 | 0.1095 | 0.1284 | 0.2931 |
| Embryoid body | 0.2055 | 0.6075 | 0.1039 | 0.1823 | 0.2228 | 0.6530 | 0.2508 | 0.2993 |

| **Supplementary Table 6.** Specificity score for each TF selected by MIMIC in 29 cell types | | | | | | | | | |
| --- | --- | --- | --- | --- | --- | --- | --- | --- | --- |
| Cell type | Hopx | Hmga1-rs1 | Basp1 | Ptma | Nupr1 | Zfp36 | Satb1 | Peg3 | Junb |
| Skeletal Muscle | 0.169 | 0.064 | 0.089 | 0.051 | 0.427 | 0.211 | 0.718 | 0.153 | 0.143 |
| G1E | 0.046 | 0.242 | 0.059 | 2.355 | 0.370 | 0.564 | 0.080 | 0.215 | 0.862 |
| G1E-ER4 | 0.046 | 2.665 | 0.036 | 2.355 | 0.332 | 0.480 | 0.115 | 0.352 | 0.885 |
| Heart | 1.212 | 1.917 | 0.081 | 0.127 | 0.400 | 0.300 | 0.279 | 0.170 | 0.187 |
| Cerebellum | 1.039 | 0.145 | 0.201 | 0.112 | 0.183 | 0.077 | 0.298 | 0.907 | 0.088 |
| Cerebrum | 0.473 | 0.171 | 1.136 | 0.127 | 0.224 | 0.052 | 0.574 | 0.666 | 0.146 |
| Whole brain (E14.5) | 0.159 | 0.127 | 1.329 | 0.457 | 0.083 | 0.071 | 0.457 | 0.624 | 0.061 |
| Whole brain (E18.5) | 0.259 | 0.193 | 1.386 | 0.347 | 0.107 | 0.061 | 0.489 | 0.579 | 0.058 |
| Lung | 1.730 | 0.099 | 0.086 | 0.240 | 0.991 | 0.824 | 0.130 | 0.098 | 0.431 |
| NIH-3T3 | 0.072 | 0.161 | 0.449 | 0.345 | 0.532 | 0.272 | 0.087 | 0.384 | 0.149 |
| Large intestine | 0.640 | 0.341 | 0.064 | 0.250 | 1.661 | 0.529 | 0.125 | 0.089 | 0.341 |
| Liver-adult-8wks | 0.645 | 0.322 | 0.291 | 0.106 | 0.106 | 0.484 | 0.103 | 0.165 | 0.175 |
| Liver-C57bl6 (E14.5) | 0.073 | 0.103 | 0.158 | 0.307 | 0.103 | 0.220 | 0.123 | 0.572 | 0.172 |
| Liver-129 (E14.5) | 0.072 | 0.550 | 0.137 | 0.329 | 0.176 | 0.240 | 0.136 | 0.554 | 0.212 |
| Kidney | 0.208 | 0.570 | 0.109 | 0.142 | 0.562 | 0.302 | 0.104 | 0.138 | 0.197 |
| Patski | 0.052 | 0.176 | 0.556 | 0.413 | 0.642 | 0.361 | 0.093 | 0.394 | 0.399 |
| Fat pad | 0.870 | 0.236 | 0.404 | 0.246 | 2.458 | 0.275 | 0.108 | 0.331 | 0.344 |
| Gonadal fat pad | 1.240 | 0.354 | 0.515 | 0.172 | 1.514 | 0.508 | 0.114 | 0.275 | 0.270 |
| 416B | 0.070 | 0.146 | 0.273 | 0.689 | 0.100 | 0.202 | 0.310 | 0.084 | 0.330 |
| A20 | 0.087 | 0.600 | 0.616 | 0.652 | 0.138 | 0.251 | 0.107 | 0.117 | 0.264 |
| B cell (CD19+) | 0.327 | 0.618 | 0.102 | 0.315 | 0.131 | 0.951 | 0.728 | 0.051 | 0.446 |
| B cell (CD43-) | 0.409 | 0.399 | 0.182 | 0.250 | 0.592 | 1.419 | 0.635 | 0.060 | 0.985 |
| CH12.LX | 0.354 | 0.301 | 0.055 | 0.124 | 0.266 | 0.148 | 0.084 | 0.045 | 1.337 |
| MEL | 0.319 | 0.026 | 0.123 | 0.705 | 0.220 | 0.094 | 0.094 | 0.078 | 0.231 |
| Spleen | 0.350 | 0.694 | 0.106 | 0.303 | 0.197 | 1.508 | 0.894 | 0.050 | 1.118 |
| T-Naive (CD4+) | 0.305 | 0.263 | 0.083 | 0.291 | 0.139 | 0.983 | 1.270 | 0.052 | 0.914 |
| Thymus | 0.137 | 0.294 | 0.192 | 0.720 | 0.258 | 0.483 | 1.886 | 0.056 | 0.181 |
| ES-E14 | 0.116 | 0.669 | 0.725 | 0.105 | 0.351 | 0.143 | 0.131 | 0.590 | 0.645 |
| Embryoid body | 0.149 | 0.051 | 0.827 | 0.782 | 0.113 | 0.104 | 0.289 | 1.534 | 0.061 |

| **Supplementary Table 7.** Specificity score for each surface marker and TF selected by MIMIC in each cell type | | | | | | | | | | | | | | | | | | |
| --- | --- | --- | --- | --- | --- | --- | --- | --- | --- | --- | --- | --- | --- | --- | --- | --- | --- | --- |
| Cell type | Lbh | Hopx | Basp1 | Peg3 | Cxcr4 | Ets1 | Epas1 | Myc | Zfp36 | Hmga1-rs1 | Lmo2 | Nfib | Nr2f2 | Ndn | Nupr1 | Satb1 | Cited4 | Cd79b |
| Skeletal Muscle | 0.0523 | 0.0980 | 0.0431 | 0.0907 | 0.0510 | 0.1526 | 0.6936 | 0.0628 | 0.1461 | 0.0259 | 0.0518 | 0.4730 | 0.3654 | 0.2949 | 0.3636 | 0.6375 | 0.8484 | 0.0000 |
| G1E | 0.0000 | 0.0000 | 0.0000 | 0.0839 | 0.0000 | 0.0000 | 0.0000 | 1.2159 | 0.4914 | 0.1443 | 0.4496 | 0.0000 | 0.0000 | 0.0000 | 0.2165 | 0.0000 | 2.3551 | 0.0000 |
| G1E-ER4 | 0.0000 | 0.0000 | 0.0000 | 0.2483 | 0.0000 | 0.0000 | 0.0000 | 1.2065 | 0.4220 | 2.0477 | 0.6630 | 0.0000 | 0.0000 | 0.0000 | 0.1689 | 0.0000 | 2.4018 | 0.0000 |
| Heart | 0.9223 | 1.2658 | 0.0615 | 0.1446 | 0.1104 | 0.4127 | 0.9909 | 0.0477 | 0.2529 | 1.9891 | 0.1579 | 0.5069 | 0.3571 | 0.3338 | 0.3378 | 0.2227 | 0.5011 | 0.1681 |
| Cerebellum | 0.1674 | 0.9645 | 0.2246 | 0.8601 | 0.0000 | 0.1090 | 0.6001 | 0.0356 | 0.0825 | 0.1429 | 0.0943 | 0.5406 | 0.3052 | 1.1624 | 0.2085 | 0.3212 | 0.3619 | 0.0000 |
| Cerebrum | 0.1625 | 0.4502 | 1.0653 | 0.6480 | 0.0439 | 0.1157 | 0.6694 | 0.0349 | 0.0000 | 0.1546 | 0.1176 | 0.4300 | 0.4525 | 1.0928 | 0.2332 | 0.5299 | 0.2880 | 0.0000 |
| Whole brain (E14.5) | 0.3744 | 0.1864 | 1.2203 | 0.6302 | 0.2234 | 0.2323 | 0.2003 | 0.1430 | 0.0795 | 0.1330 | 0.1464 | 0.9133 | 1.0355 | 1.5036 | 0.0744 | 0.4878 | 0.0000 | 0.0000 |
| Whole brain (E18.5) | 0.2903 | 0.2774 | 1.3124 | 0.5873 | 0.2286 | 0.1840 | 0.1893 | 0.1491 | 0.0613 | 0.1875 | 0.1191 | 0.7898 | 0.8658 | 1.5869 | 0.1258 | 0.5029 | 0.0000 | 0.0000 |
| Lung | 0.5860 | 1.7514 | 0.1245 | 0.1447 | 0.4005 | 0.8290 | 1.9896 | 0.0891 | 0.8634 | 0.1162 | 0.2737 | 0.5966 | 0.7410 | 0.4684 | 1.1026 | 0.1639 | 0.0000 | 0.6599 |
| NIH-3T3 | 0.5350 | 0.0000 | 0.5498 | 0.4583 | 0.0000 | 0.2921 | 0.0000 | 0.1262 | 0.3169 | 0.1674 | 0.0666 | 0.7158 | 1.3115 | 0.7914 | 0.6686 | 0.0000 | 0.0000 | 0.0000 |
| Large intestine | 0.3255 | 0.7417 | 0.0576 | 0.0889 | 0.0414 | 0.1054 | 0.7248 | 0.1847 | 0.6249 | 0.3699 | 0.0272 | 0.3141 | 0.3965 | 0.2451 | 1.8149 | 0.0969 | 0.4169 | 0.1482 |
| Liver-adult-8wks | 0.0592 | 0.6660 | 0.2620 | 0.1330 | 0.0000 | 0.2133 | 0.7445 | 0.0849 | 0.4903 | 0.2831 | 0.1299 | 0.4064 | 0.5718 | 0.1521 | 0.0000 | 0.0000 | 0.5107 | 0.0000 |
| Liver-C57bl6 (E14.5) | 0.1629 | 0.0527 | 0.1933 | 0.7018 | 0.1633 | 0.3248 | 0.2241 | 0.3996 | 0.2729 | 0.1078 | 0.8443 | 0.1911 | 0.4963 | 0.3379 | 0.0850 | 0.0862 | 1.4627 | 0.2391 |
| Liver-129 (E14.5) | 0.0946 | 0.0000 | 0.1381 | 0.6621 | 0.1372 | 0.2320 | 0.1096 | 0.4141 | 0.2599 | 0.6788 | 0.8186 | 0.1148 | 0.3121 | 0.1735 | 0.1644 | 0.0897 | 1.3716 | 0.2894 |
| Kidney | 0.1565 | 0.2129 | 0.1157 | 0.1502 | 0.0526 | 0.1978 | 0.7574 | 0.0578 | 0.3268 | 0.6451 | 0.1071 | 0.3244 | 0.7940 | 0.1555 | 0.6687 | 0.0000 | 0.3785 | 0.0000 |
| Patski | 0.1903 | 0.0000 | 0.3978 | 0.3139 | 0.2155 | 0.3332 | 0.0000 | 0.1745 | 0.2761 | 0.1161 | 0.0334 | 0.1991 | 0.3035 | 0.4000 | 0.5035 | 0.0000 | 0.0000 | 0.6564 |
| Fat pad | 0.0818 | 0.9655 | 0.4545 | 0.3819 | 0.0000 | 0.0713 | 0.0949 | 0.4401 | 0.3047 | 0.2376 | 0.3107 | 0.5777 | 0.2584 | 0.0000 | 2.5672 | 0.0000 | 0.8584 | 0.1523 |
| Gonadal fat pad | 0.1853 | 1.3955 | 0.5162 | 0.2213 | 0.0487 | 0.0751 | 0.1479 | 0.3099 | 0.4819 | 0.3023 | 0.0000 | 0.6290 | 0.3894 | 0.0000 | 1.8722 | 0.0000 | 0.0000 | 0.1219 |
| 416B | 0.2395 | 0.0000 | 0.3667 | 0.0000 | 0.6548 | 0.0000 | 0.0000 | 0.9159 | 0.2583 | 0.1712 | 0.4904 | 0.0989 | 0.0000 | 0.2120 | 0.0000 | 0.3559 | 0.0000 | 0.0000 |
| A20 | 0.1793 | 0.0000 | 0.7343 | 0.0911 | 0.7655 | 0.6808 | 0.0000 | 0.5557 | 0.3086 | 0.6990 | 0.1023 | 0.0000 | 0.0000 | 0.0000 | 0.0906 | 0.0000 | 0.0000 | 1.5384 |
| B cell (CD19+) | 0.9885 | 0.3564 | 0.0823 | 0.0000 | 0.9052 | 1.5032 | 0.0000 | 0.4278 | 1.0082 | 0.6563 | 0.5806 | 0.0000 | 0.0000 | 0.0000 | 0.0780 | 0.7408 | 0.0000 | 2.4882 |
| B cell (CD43-) | 0.8159 | 0.4058 | 0.1854 | 0.0000 | 0.9617 | 1.1954 | 0.1176 | 0.5988 | 1.5279 | 0.3592 | 0.6513 | 0.0598 | 0.0000 | 0.0000 | 0.6534 | 0.5992 | 0.0000 | 2.1257 |
| CH12.LX | 0.1769 | 0.3667 | 0.0000 | 0.0000 | 0.9550 | 0.1853 | 0.0000 | 0.4447 | 0.1212 | 0.2958 | 0.5903 | 0.0000 | 0.0000 | 0.0000 | 0.2505 | 0.0000 | 0.0000 | 1.8395 |
| MEL | 0.0000 | 0.3959 | 0.1156 | 0.0000 | 0.1497 | 0.0000 | 0.0000 | 0.8213 | 0.0487 | 0.0000 | 0.6394 | 0.0000 | 0.0000 | 0.0000 | 0.2233 | 0.0000 | 0.5088 | 0.0000 |
| Spleen | 0.7973 | 0.3456 | 0.0936 | 0.0000 | 1.0755 | 1.3849 | 0.0000 | 0.5469 | 1.5401 | 0.6707 | 0.3496 | 0.0000 | 0.0000 | 0.0000 | 0.1837 | 0.8325 | 0.0000 | 2.1779 |
| T-Naive (CD4+) | 0.9790 | 0.3647 | 0.0480 | 0.0000 | 0.4663 | 1.8051 | 0.2120 | 0.5289 | 1.1555 | 0.2950 | 0.0312 | 0.0000 | 0.0000 | 0.0000 | 0.0886 | 1.3761 | 0.0000 | 0.3659 |
| Thymus | 0.7617 | 0.1346 | 0.2173 | 0.0000 | 1.0392 | 1.4552 | 0.1383 | 0.3237 | 0.5389 | 0.2921 | 0.0496 | 0.1085 | 0.0000 | 0.0000 | 0.2798 | 1.6845 | 0.0000 | 0.4984 |
| ES-E14 | 0.0379 | 0.0794 | 0.6259 | 0.4751 | 0.0000 | 0.1579 | 0.1068 | 0.1283 | 0.1083 | 0.5054 | 0.0000 | 0.0652 | 0.0000 | 0.6531 | 0.2941 | 0.0000 | 0.2481 | 0.1181 |
| Embryoid body | 0.3991 | 0.1781 | 0.8207 | 1.3947 | 0.1893 | 0.4291 | 0.2422 | 0.2297 | 0.1281 | 0.0509 | 0.1655 | 0.2500 | 1.0082 | 1.0271 | 0.1285 | 0.3342 | 0.4610 | 0.0000 |

| **Supplementary Table 8.** Difference score for each surface marker selected by MIMIC in an extended dataset | | | | | | | | | |
| --- | --- | --- | --- | --- | --- | --- | --- | --- | --- |
| Cell types | Adam8 | Dpp4 | Csf1r | Cxcr4 | Cd22 | Cd24a | Cd180 | Cd247 | Cd79b |
| Adipocytes | 0.9591 | 0.9181 | 0.997 | 0.9639 | 0.9915 | 0.996 | 0.9878 | 0.7674 | 0.9967 |
| Adipocytes (cultured, brown) | 0.9967 | 0.967 | 0.9181 | 0.9986 | 0.946 | 0.9954 | 0.9965 | 0.9871 | 0.6458 |
| Colon | 0.9914 | 0.0543 | 0.9162 | 0.9295 | 0.1713 | 0.2565 | 0.97 | 0.9449 | 0.2433 |
| DC CD11+ | 0.3837 | 0.0308 | 0.5549 | 0.5107 | 0.0128 | 0.5671 | 0.323 | 0.9966 | 0.2064 |
| DC CD8+ | 0.1795 | 0.0315 | 0.802 | 0.5825 | 0.1676 | 0.1456 | 0.2608 | 0.9794 | 0.3532 |
| Double negative 2a (DN2a) thymic cells | 0.9064 | 0.0878 | 0.9921 | 0.2371 | 0.799 | 0.2613 | 0.8967 | 0.6026 | 0.4818 |
| Double negative 2b (DN2b) thymic cells | 0.9574 | 0.0667 | 0.9951 | 0.1884 | 0.8273 | 0.2694 | 0.9803 | 0.1079 | 0.5168 |
| Blastocyst (Early) ICM | 0.9488 | 0.5043 | 0.7394 | 0.9929 | 0.1055 | 0.9615 | 0.9776 | 0.9574 | 0.3959 |
| 2C Embyro (early) | 0.9943 | 0.9713 | 0.8859 | 0.7451 | 0.4225 | 0.9898 | 0.8339 | 0.8785 | 0.9691 |
| Epiblast | 0.6556 | 0.4358 | 0.8592 | 0.9929 | 0.6844 | 0.9851 | 0.9946 | 0.9141 | 0.9652 |
| Embryonic stem cells (B6J) | 0.9344 | 0.4253 | 0.9946 | 0.9348 | 0.968 | 0.9045 | 0.9156 | 0.8351 | 0.5918 |
| Granulocytes | 0.0107 | 0.8454 | 0.1382 | 0.032 | 0.3844 | 0.1969 | 0.3777 | 0.8657 | 0.3537 |
| Inner cell mass (E3.5) | 0.8769 | 0.3801 | 0.692 | 0.9913 | 0.3842 | 0.9316 | 0.9582 | 0.9682 | 0.4278 |
| Innate lymphoid cell (Type 2) | 0.0786 | 0.4884 | 0.9776 | 0.2017 | 0.4674 | 0.9727 | 0.476 | 0.2718 | 0.0521 |
| Intestine | 0.959 | 0.009 | 0.3882 | 0.8666 | 0.272 | 0.5137 | 0.6073 | 0.8967 | 0.1862 |
| Kidney | 0.976 | 0.1393 | 0.2359 | 0.939 | 0.9085 | 0.6283 | 0.8193 | 0.9006 | 0.8818 |
| Blastocyst (Late) PrE | 0.6565 | 0.3433 | 0.6449 | 0.9902 | 0.3445 | 0.9537 | 0.9547 | 0.9821 | 0.2202 |
| Liver | 0.9691 | 0.0766 | 0.2801 | 0.9539 | 0.6586 | 0.933 | 0.6511 | 0.8863 | 0.7024 |
| Lung fibroblasts | 0.7766 | 0.9897 | 0.9528 | 0.9374 | 0.8285 | 0.9992 | 0.8796 | 0.7879 | 0.9553 |
| Blastocyst (Mid) ICM | 0.7235 | 0.3273 | 0.8537 | 0.9861 | 0.1504 | 0.9693 | 0.7292 | 0.6898 | 0.7436 |
| Mast cell -IL10 -LPS | 0.0446 | 0.9256 | 0.6095 | 0.6281 | 0.7329 | 0.9264 | 0.0317 | 0.6749 | 0.8347 |
| Mast cell -IL10 +LPS | 0.1429 | 0.9129 | 0.6289 | 0.6529 | 0.794 | 0.9466 | 0.0365 | 0.6512 | 0.6687 |
| Mast cell +IL10 -LPS | 0.0353 | 0.9158 | 0.6243 | 0.6655 | 0.7732 | 0.921 | 0.0536 | 0.6757 | 0.8197 |
| Mast cell +IL10 +LPS | 0.1128 | 0.9187 | 0.6312 | 0.6893 | 0.846 | 0.9435 | 0.1074 | 0.5717 | 0.7138 |
| Neonatal tail fibroblast | 0.3019 | 0.9498 | 0.9396 | 0.9236 | 0.9638 | 0.919 | 0.9397 | 0.9245 | 0.9497 |
| Neutrophil +IL10 +LPS | 0.0294 | 0.4866 | 0.2403 | 0.1182 | 0.2073 | 0.201 | 0.4095 | 0.4476 | 0.0465 |
| Oocytes | 0.2708 | 0.9807 | 0.8499 | 0.7698 | 0.5957 | 0.997 | 0.188 | 0.1918 | 0.9518 |
| Spermatocytes (pachytene) | 0.9559 | 0.9003 | 0.9881 | 0.8906 | 0.9645 | 0.9961 | 0.9677 | 0.956 | 0.9515 |
| Pronuclei | 0.4958 | 0.9857 | 0.8378 | 0.9488 | 0.9674 | 0.9992 | 0.6132 | 0.4172 | 0.9592 |
| splenic DC -IL10 -LPS | 0.0116 | 0.0159 | 0.1138 | 0.0465 | 0.1838 | 0.7063 | 0.0831 | 0.2358 | 0.0659 |
| splenic DC -IL10 +LPS | 0.0197 | 0.0563 | 0.546 | 0.1426 | 0.4565 | 0.6444 | 0.0457 | 0.2286 | 0.1257 |
| splenic DC +IL10 -LPS | 0.0121 | 0.0165 | 0.1088 | 0.0522 | 0.236 | 0.6635 | 0.1331 | 0.239 | 0.0751 |
| splenic DC +IL10 +LPS | 0.0206 | 0.0643 | 0.3065 | 0.1174 | 0.4661 | 0.6635 | 0.1666 | 0.215 | 0.1493 |
| Sperm | 0.0424 | 0.095 | 0.1226 | 0.3624 | 0.1304 | 0.4935 | 0.0193 | 0.896 | 0.2951 |
| Adipose | 0.9988 | 0.5991 | 0.9649 | 0.9611 | 0.9754 | 0.9536 | 0.9842 | 0.583 | 0.9823 |
| Monocyte | 0.9914 | 0.1188 | 0.7754 | 0.0816 | 0.788 | 0.341 | 0.2569 | 0.0605 | 0.6555 |
| BMDM | 0.5308 | 0.8956 | 0.2018 | 0.2848 | 0.7626 | 0.9369 | 0.278 | 0.727 | 0.8506 |
| BMDM +CpolyG | 0.2168 | 0.8956 | 0.2018 | 0.8681 | 0.8546 | 0.9019 | 0.6108 | 0.3296 | 0.9266 |
| BMDM +IL-1b | 0.1906 | 0.8956 | 0.2018 | 0.6808 | 0.8364 | 0.8894 | 0.4125 | 0.5945 | 0.9035 |
| BMDM -lipidA | 0.2864 | 0.8686 | 0.2018 | 0.3404 | 0.7505 | 0.8844 | 0.3456 | 0.9046 | 0.9035 |
| BMDM +lipidA 20 mins | 0.3376 | 0.8956 | 0.5037 | 0.9377 | 0.7835 | 0.916 | 0.3841 | 0.3248 | 0.9324 |
| BMDM +lipidA 30 mins | 0.3245 | 0.985 | 0.2018 | 0.3636 | 0.7648 | 0.8938 | 0.3637 | 0.8143 | 0.9035 |
| BMDM +lipidA 360 mins | 0.5439 | 0.9181 | 0.589 | 0.9601 | 0.8127 | 0.9604 | 0.3904 | 0.2407 | 0.989 |
| BMDM +lipidA 60 mins | 0.3903 | 0.985 | 0.4592 | 0.5394 | 0.7717 | 0.9042 | 0.3927 | 0.4758 | 0.9266 |
| BMDM +MALP2 | 0.1906 | 0.9104 | 0.2018 | 0.9252 | 0.8577 | 0.9297 | 0.5599 | 0.3364 | 0.9087 |
| BMDM +IFNg | 0.3245 | 0.8751 | 0.3855 | 0.8916 | 0.7984 | 0.921 | 0.1865 | 0.7898 | 0.9035 |
| BMDM +IL-4 | 0.1235 | 0.9029 | 0.4592 | 0.8328 | 0.8087 | 0.923 | 0.5777 | 0.8411 | 0.885 |
| BMDM +IFNa | 0.1906 | 0.8886 | 0.2018 | 0.7088 | 0.8423 | 0.8634 | 0.5241 | 0.3889 | 0.9154 |
| BMDM +TGFb | 0.2168 | 0.8956 | 0.3855 | 0.2311 | 0.8035 | 0.8363 | 0.2848 | 0.6603 | 0.9035 |
| Brain | 0.9973 | 0.8773 | 0.956 | 0.9927 | 0.9928 | 0.9715 | 0.9941 | 0.8708 | 0.9967 |
| Cerebellum | 0.9987 | 0.9487 | 0.9799 | 0.9937 | 0.9226 | 0.9602 | 0.9941 | 0.9076 | 0.9891 |
| Cerebrum | 0.9955 | 0.9303 | 0.8549 | 0.986 | 0.9464 | 0.8314 | 0.9859 | 0.974 | 0.9877 |
| Cortex | 0.9971 | 0.9535 | 0.8606 | 0.9934 | 0.9283 | 0.8574 | 0.9844 | 0.9966 | 0.989 |
| Dendritic cells (BMDC) | 0.2397 | 0.7567 | 0.2397 | 0.7275 | 0.2773 | 0.2681 | 0.5989 | 0.9606 | 0.8797 |
| Dentate gyrus | 0.9739 | 0.9818 | 0.8212 | 0.9335 | 0.9738 | 0.9209 | 0.991 | 0.9835 | 0.9877 |
| Dermal fibroblast | 0.7009 | 0.9582 | 0.8585 | 0.8624 | 0.8367 | 0.9739 | 0.9176 | 0.9657 | 0.9868 |
| Spermatids (elogated) | 0.9943 | 0.9936 | 0.8107 | 0.9929 | 0.9687 | 0.9738 | 0.9927 | 0.9009 | 0.97 |
| 8C Embryo (single-cell) | 0.9906 | 0.9259 | 0.665 | 0.9552 | 0.2852 | 0.9075 | 0.9778 | 0.959 | 0.969 |
| Heart | 0.9658 | 0.5678 | 0.1736 | 0.5894 | 0.8324 | 0.9574 | 0.7219 | 0.8192 | 0.6925 |
| Hippocampus | 0.9481 | 0.883 | 0.1903 | 0.9596 | 0.9373 | 0.9312 | 0.8019 | 0.9847 | 0.9569 |
| Spermatocytes (leptotene) | 0.9587 | 0.9649 | 0.7586 | 0.8621 | 0.9625 | 0.9809 | 0.9175 | 0.9818 | 0.9585 |
| Macrophages -IL10 -LPS | 0.0067 | 0.9118 | 0.0224 | 0.0759 | 0.0302 | 0.7074 | 0.0062 | 0.765 | 0.3388 |
| Macrophages -IL10 +LPS | 0.028 | 0.8759 | 0.0419 | 0.6523 | 0.0783 | 0.7638 | 0.0119 | 0.4651 | 0.4274 |
| Macrophages +IL10 -LPS | 0.0093 | 0.8723 | 0.0244 | 0.0962 | 0.0351 | 0.7866 | 0.0053 | 0.836 | 0.3939 |
| Macrophages +IL10 +LPS | 0.0287 | 0.8537 | 0.047 | 0.5616 | 0.0779 | 0.7428 | 0.0203 | 0.5319 | 0.3337 |
| Megakaryoblast | 0.2724 | 0.9891 | 0.3476 | 0.8088 | 0.6936 | 0.9751 | 0.1055 | 0.4562 | 0.9006 |
| Microglia | 0.4379 | 0.9741 | 0.0056 | 0.9392 | 0.213 | 0.9946 | 0.0023 | 0.1085 | 0.0685 |
| Blastocyst (Mid) TE | 0.984 | 0.9457 | 0.7181 | 0.9898 | 0.4024 | 0.976 | 0.7988 | 0.9714 | 0.2867 |
| Morula (single-cell) | 0.6579 | 0.7315 | 0.6435 | 0.8466 | 0.1948 | 0.9231 | 0.9093 | 0.6171 | 0.8523 |
| Myeloid cells | 0.0128 | 0.5532 | 0.0337 | 0.2022 | 0.0716 | 0.6717 | 0.0116 | 0.5174 | 0.7463 |
| Myoblast | 0.153 | 0.9676 | 0.2109 | 0.8078 | 0.8225 | 0.6762 | 0.2747 | 0.6575 | 0.9503 |
| Nucleus accumbens | 0.9791 | 0.9589 | 0.3418 | 0.9727 | 0.9334 | 0.9762 | 0.8244 | 0.9318 | 0.9541 |
| Olfactory bulb | 0.966 | 0.8815 | 0.4547 | 0.7626 | 0.923 | 0.9461 | 0.8807 | 0.7691 | 0.953 |
| Pineal gland | 0.9372 | 0.7007 | 0.3513 | 0.8217 | 0.2279 | 0.8568 | 0.7284 | 0.6748 | 0.1326 |
| Placenta | 0.3216 | 0.7251 | 0.0511 | 0.846 | 0.8911 | 0.9345 | 0.9242 | 0.4552 | 0.953 |
| pre-Adipocytes | 0.795 | 0.7833 | 0.861 | 0.9746 | 0.8762 | 0.9746 | 0.8715 | 0.9549 | 0.9552 |
| pre-Adipocytes (brown) | 0.8685 | 0.6914 | 0.8557 | 0.9547 | 0.9647 | 0.9914 | 0.8313 | 0.8667 | 0.9541 |
| Adipocytes (white) | 0.7428 | 0.3626 | 0.5212 | 0.6018 | 0.4106 | 0.9566 | 0.609 | 0.1864 | 0.5286 |
| Promyelocytes | 0.845 | 0.3207 | 0.1128 | 0.0845 | 0.732 | 0.4436 | 0.1859 | 0.6979 | 0.0815 |
| Spermatids (round) | 0.9902 | 0.9858 | 0.7725 | 0.9828 | 0.9658 | 0.9801 | 0.9658 | 0.5416 | 0.9585 |
| Spermatogonia | 0.2783 | 0.9874 | 0.0989 | 0.5298 | 0.782 | 0.9678 | 0.2403 | 0.8703 | 0.9613 |
| Spinal cord | 0.9779 | 0.9302 | 0.3728 | 0.9296 | 0.9596 | 0.7389 | 0.8623 | 0.876 | 0.9611 |
| Striatum | 0.9467 | 0.9689 | 0.2572 | 0.9915 | 0.9219 | 0.9813 | 0.885 | 0.8468 | 0.962 |
| Subventricular zone | 0.9438 | 0.9682 | 0.3297 | 0.8198 | 0.942 | 0.693 | 0.923 | 0.664 | 0.9613 |
| tail fibroblast | 0.4932 | 0.8843 | 0.7051 | 0.9066 | 0.8475 | 0.9607 | 0.8082 | 0.8922 | 0.9611 |
| Testicular cells | 0.891 | 0.9808 | 0.9131 | 0.9483 | 0.9539 | 0.9921 | 0.9645 | 0.6444 | 0.9613 |
| Polyploid trophoblast giant cells (E9.5) | 0.1698 | 0.8821 | 0.057 | 0.8999 | 0.7641 | 0.9729 | 0.9057 | 0.9228 | 0.6679 |
| Trophoblast stem cells | 0.9827 | 0.9865 | 0.4327 | 0.9919 | 0.8769 | 0.9743 | 0.9678 | 0.9553 | 0.9079 |
| Spermatogonia (type A) | 0.9755 | 0.9567 | 0.971 | 0.7875 | 0.9651 | 0.9758 | 0.9678 | 0.868 | 0.9613 |
| Spermatogonia (type B) | 0.9753 | 0.9655 | 0.7989 | 0.7942 | 0.9649 | 0.9721 | 0.9028 | 0.9544 | 0.9611 |
| White-matter glia | 0.9778 | 0.958 | 0.1431 | 0.2179 | 0.9644 | 0.7358 | 0.1401 | 0.2667 | 0.9163 |
| Zygote | 0.9843 | 0.986 | 0.6582 | 0.5343 | 0.6292 | 0.9868 | 0.417 | 0.6502 | 0.9592 |
| Blastocyst | 0.9996 | 0.8113 | 0.9991 | 0.9981 | 0.583 | 0.9867 | 0.9959 | 0.0347 | 0.9967 |
| CD4+ iTreg cells | 0.9799 | 0.1482 | 0.9824 | 0.9182 | 0.8939 | 0.9562 | 0.9988 | 0.0099 | 0.7116 |
| CD4+ T cells -IL-21 | 0.8974 | 0.1079 | 0.9999 | 0.7015 | 0.8173 | 0.9526 | 0.9781 | 0.0095 | 0.4702 |
| Naive CD4+ T cells, anti-CD3/28 treated | 0.8534 | 0.9869 | 0.5362 | 0.9997 | 0.926 | 0.8968 | 0.938 | 0.9987 | 0.009 |
| CD4+ T cells +IL-21 | 0.9001 | 0.1322 | 0.9999 | 0.8488 | 0.7156 | 0.9549 | 0.9792 | 0.01 | 0.4096 |
| CD4+ Th17 cells | 0.952 | 0.183 | 0.9902 | 0.7194 | 0.8351 | 0.8264 | 0.9968 | 0.0159 | 0.7504 |
| CD4+ Th1 cells | 0.7495 | 0.1493 | 0.9905 | 0.7877 | 0.6552 | 0.9104 | 0.9978 | 0.0127 | 0.9681 |
| CD4+ Th2 cells | 0.7397 | 0.2251 | 0.9943 | 0.6445 | 0.6992 | 0.8209 | 0.9942 | 0.0244 | 0.3716 |
| CD4+ Th9 cells | 0.998 | 0.6627 | 0.9998 | 0.8589 | 0.9261 | 0.9508 | 0.9986 | 0.0782 | 0.2668 |
| CD4+ Treg cells | 0.9918 | 0.2747 | 0.9982 | 0.7305 | 0.8581 | 0.9377 | 0.9974 | 0.1115 | 0.3433 |
| CD4+ T cells (anti-CD3/CD28 treated) | 0.9413 | 0.6084 | 0.9895 | 0.3987 | 0.3497 | 0.6531 | 0.9419 | 0.121 | 0.0982 |
| CD4+ T cells | 0.9514 | 0.1777 | 0.9906 | 0.2422 | 0.4465 | 0.8235 | 0.9491 | 0.0715 | 0.103 |
| CD8+ T cells | 0.9205 | 0.2383 | 0.8621 | 0.9704 | 0.1441 | 0.4845 | 0.9549 | 0.0979 | 0.0692 |
| CD8+ T cells +IL-2 | 0.8682 | 0.341 | 0.9913 | 0.9682 | 0.2336 | 0.6819 | 0.9927 | 0.141 | 0.2064 |
| Double positive thymic cells | 0.9586 | 0.2481 | 0.9987 | 0.1568 | 0.8414 | 0.3455 | 0.9875 | 0.0652 | 0.2893 |
| Inner cell mass (E3.75) | 0.2627 | 0.6695 | 0.961 | 0.9911 | 0.1709 | 0.7272 | 0.6641 | 0.108 | 0.9569 |
| Intestinal subepithelial myofibroblasts (neonatal) | 0.9487 | 0.9798 | 0.9804 | 0.9911 | 0.6757 | 0.9417 | 0.4841 | 0.4754 | 0.9569 |
| Naive CD4+ CD25- T cells | 0.7892 | 0.1567 | 0.6772 | 0.2231 | 0.2808 | 0.9571 | 0.4284 | 0.0063 | 0.2498 |
| Adipocytes (brown) | 0.9906 | 0.9734 | 0.9424 | 0.8161 | 0.8586 | 0.9832 | 0.8715 | 0.1354 | 0.9223 |
| Spermatids | 0.9906 | 0.9856 | 0.9398 | 0.9921 | 0.9387 | 0.9706 | 0.9668 | 0.3308 | 0.962 |
| Spermatocytes | 0.9295 | 0.9855 | 0.9952 | 0.9727 | 0.9174 | 0.992 | 0.9712 | 0.7212 | 0.9614 |
| Basophilic erythroblast | 0.9728 | 0.7414 | 0.9916 | 0.3004 | 0.8551 | 0.0981 | 0.9774 | 0.2817 | 0.8882 |
| Bone marrow | 0.8406 | 0.3877 | 0.9652 | 0.3241 | 0.0512 | 0.1493 | 0.956 | 0.583 | 0.0319 |
| Caecum (E13.5) | 0.998 | 0.0921 | 0.9939 | 0.8916 | 0.9954 | 0.2648 | 0.995 | 0.9046 | 0.9967 |
| Cardiac precursors | 0.9987 | 0.8718 | 0.9999 | 0.9606 | 0.9954 | 0.37 | 0.9978 | 0.727 | 0.9953 |
| Cardiomyocytes | 0.9994 | 0.9547 | 0.9995 | 0.9768 | 0.9931 | 0.6203 | 0.9959 | 0.6179 | 0.9948 |
| Cerebellar granular neurons | 0.9412 | 0.9865 | 0.975 | 0.8806 | 0.9104 | 0.1376 | 0.9735 | 0.9963 | 0.9901 |
| Cortex neuron | 0.9754 | 0.9658 | 0.9805 | 0.8704 | 0.8119 | 0.0823 | 0.9801 | 0.9383 | 0.9881 |
| Cortical thymic epithelial | 0.9625 | 0.9659 | 0.9994 | 0.9924 | 0.9078 | 0.9122 | 0.9802 | 0.9601 | 0.9768 |
| Double negative 1 (DN1) thymic cells | 0.9347 | 0.294 | 0.8778 | 0.4179 | 0.8469 | 0.4063 | 0.7492 | 0.7632 | 0.3922 |
| Double negative 3 (DN3) thymic cells | 0.965 | 0.1022 | 0.9991 | 0.2488 | 0.821 | 0.237 | 0.9894 | 0.1085 | 0.2173 |
| Dorsal rootganglia | 0.6793 | 0.9742 | 0.9964 | 0.9885 | 0.9626 | 0.2536 | 0.9964 | 0.992 | 0.9866 |
| Central neural epithelium (E10.5) | 0.8971 | 0.9954 | 0.9851 | 0.6275 | 0.4133 | 0.2324 | 0.9903 | 0.3846 | 0.987 |
| Dorsal control neural epithelium (E10.5) | 0.8988 | 0.9844 | 0.999 | 0.8208 | 0.5246 | 0.2448 | 0.9902 | 0.5247 | 0.9868 |
| Forelimb (E10.5) | 0.9731 | 0.9874 | 0.9761 | 0.8926 | 0.9348 | 0.1903 | 0.9932 | 0.9749 | 0.9663 |
| Hindlimb (E10.5) | 0.9713 | 0.9928 | 0.9762 | 0.9368 | 0.9688 | 0.2478 | 0.9952 | 0.9618 | 0.9567 |
| Lateral prominence neural epithelium (E10.5) | 0.9512 | 0.9904 | 0.944 | 0.9761 | 0.4065 | 0.2878 | 0.9724 | 0.44 | 0.9861 |
| Mandibular arch (E10.5) | 0.9739 | 0.9746 | 0.8952 | 0.9967 | 0.4358 | 0.1299 | 0.9827 | 0.7643 | 0.9859 |
| Maxillary arch (E10.5) | 0.9969 | 0.978 | 0.9069 | 0.9762 | 0.254 | 0.1924 | 0.919 | 0.4075 | 0.9731 |
| Medial nasal prominence (E10.5) | 0.9484 | 0.9944 | 0.5977 | 0.9961 | 0.088 | 0.3464 | 0.9364 | 0.1984 | 0.984 |
| Cortical neurons (E16.5) | 0.9637 | 0.9897 | 0.9922 | 0.7836 | 0.9695 | 0.1384 | 0.9891 | 0.9748 | 0.9828 |
| Dermis (E16.5) | 0.934 | 0.5564 | 0.7178 | 0.8091 | 0.969 | 0.6609 | 0.9882 | 0.9671 | 0.9814 |
| Epidermis (E16.5) | 0.8545 | 0.2989 | 0.484 | 0.843 | 0.9454 | 0.4744 | 0.9781 | 0.8864 | 0.9799 |
| Skin (E16.5) | 0.9813 | 0.3483 | 0.4608 | 0.7471 | 0.9688 | 0.4215 | 0.9874 | 0.9732 | 0.9612 |
| Caudal brain neural epithelium (E8.5) | 0.83 | 0.9944 | 0.9804 | 0.4102 | 0.6556 | 0.1608 | 0.987 | 0.3108 | 0.976 |
| Non-floor plate neural epithelium (E8.5) | 0.8793 | 0.9943 | 0.9967 | 0.7315 | 0.6853 | 0.4087 | 0.9799 | 0.3249 | 0.9734 |
| Cranial mesenchyme (E9.5) | 0.9715 | 0.9439 | 0.8801 | 0.8747 | 0.5828 | 0.438 | 0.9693 | 0.5914 | 0.9731 |
| Epidermal ectoderm (E9.5) | 0.9196 | 0.9769 | 0.9513 | 0.361 | 0.7249 | 0.0724 | 0.986 | 0.331 | 0.9728 |
| Mandibular arch (E9.5) | 0.9186 | 0.9941 | 0.9964 | 0.8599 | 0.634 | 0.3985 | 0.9408 | 0.5261 | 0.9725 |
| Maxillary arch (E9.5) | 0.8331 | 0.9616 | 0.9139 | 0.9362 | 0.7731 | 0.2262 | 0.9718 | 0.4712 | 0.9722 |
| 2C Embyro (late) | 0.8168 | 0.9902 | 0.8271 | 0.9612 | 0.3017 | 0.8422 | 0.9027 | 0.9934 | 0.8942 |
| 2C Embyro (middle) | 0.9929 | 0.9934 | 0.8552 | 0.9738 | 0.2601 | 0.7682 | 0.9294 | 0.9934 | 0.9691 |
| 4C Embryo (single-cell) | 0.9685 | 0.9583 | 0.7847 | 0.8765 | 0.2587 | 0.8987 | 0.9803 | 0.9712 | 0.5538 |
| 2C Embryo | 0.9557 | 0.9483 | 0.9313 | 0.9605 | 0.4426 | 0.9252 | 0.9394 | 0.7848 | 0.9681 |
| Eosinophils -IL-10 -LPS | 0.0163 | 0.9603 | 0.1769 | 0.0684 | 0.7395 | 0.1141 | 0.738 | 0.9774 | 0.4285 |
| Eosinophils -IL-10 +LPS | 0.0184 | 0.9206 | 0.1375 | 0.0715 | 0.7872 | 0.116 | 0.7268 | 0.9435 | 0.4176 |
| Basal epidermis (E14.5) | 0.9771 | 0.9405 | 0.9961 | 0.8856 | 0.9574 | 0.3596 | 0.9888 | 0.8549 | 0.9651 |
| Suprabasal epidermis (E14.5) | 0.9929 | 0.576 | 0.9961 | 0.987 | 0.8252 | 0.478 | 0.9521 | 0.9927 | 0.9649 |
| Epididymis | 0.9854 | 0.0643 | 0.6191 | 0.987 | 0.9253 | 0.3402 | 0.9778 | 0.9811 | 0.9155 |
| EpiSC | 0.8868 | 0.9806 | 0.9891 | 0.6979 | 0.9607 | 0.4698 | 0.9874 | 0.9586 | 0.9647 |
| Erythroblast | 0.8037 | 0.9898 | 0.6557 | 0.8682 | 0.7787 | 0.1289 | 0.8213 | 0.7781 | 0.8067 |
| Erythroid progenitor | 0.9076 | 0.8231 | 0.467 | 0.5887 | 0.7493 | 0.149 | 0.5682 | 0.9555 | 0.2975 |
| Embryonic stem cells (E14) | 0.8694 | 0.7531 | 0.9605 | 0.9644 | 0.9147 | 0.8174 | 0.9648 | 0.9679 | 0.7836 |
| Naive ESC (Hex-) | 0.9827 | 0.7198 | 0.9221 | 0.9761 | 0.9563 | 0.8694 | 0.8721 | 0.7236 | 0.4919 |
| Naive ESC (Hex+) | 0.9689 | 0.7085 | 0.9473 | 0.9706 | 0.9138 | 0.8965 | 0.9241 | 0.7541 | 0.6439 |
| Embryonic stem cells (V6.5) | 0.8085 | 0.783 | 0.9944 | 0.9395 | 0.9677 | 0.7698 | 0.9612 | 0.8935 | 0.9619 |
| Embryonic stem cells (ZHBTc4) | 0.8405 | 0.8246 | 0.904 | 0.9738 | 0.6317 | 0.9413 | 0.9509 | 0.951 | 0.7644 |
| Eye | 0.9778 | 0.6913 | 0.5248 | 0.9752 | 0.9669 | 0.2251 | 0.9362 | 0.9827 | 0.9604 |
| Fatpad | 0.8752 | 0.5883 | 0.5769 | 0.9621 | 0.9663 | 0.3359 | 0.7299 | 0.8709 | 0.9047 |
| Frontal cortex | 0.9658 | 0.787 | 0.3323 | 0.8608 | 0.9658 | 0.299 | 0.844 | 0.9866 | 0.9569 |
| Genital fatpad | 0.9843 | 0.5051 | 0.6502 | 0.8437 | 0.799 | 0.3004 | 0.787 | 0.765 | 0.8199 |
| Hair follicle | 0.9783 | 0.6732 | 0.9544 | 0.7383 | 0.9383 | 0.4764 | 0.9641 | 0.9581 | 0.9587 |
| Hippocampus neuron | 0.4975 | 0.9596 | 0.4751 | 0.8688 | 0.8302 | 0.3892 | 0.7763 | 0.9454 | 0.9555 |
| Hippocampus tissue | 0.9778 | 0.9453 | 0.3104 | 0.7509 | 0.964 | 0.2977 | 0.8858 | 0.9713 | 0.9225 |
| Hematopoietic stem cells | 0.5724 | 0.2835 | 0.7854 | 0.0587 | 0.7036 | 0.2407 | 0.4036 | 0.6228 | 0.0741 |
| Ileum | 0.9396 | 0.1163 | 0.814 | 0.6773 | 0.9638 | 0.3532 | 0.8531 | 0.9642 | 0.9143 |
| Intestinal subepithelial myofibroblasts (adult) | 0.3188 | 0.9889 | 0.6463 | 0.8765 | 0.7079 | 0.3442 | 0.366 | 0.4173 | 0.9583 |
| Keratinocyte | 0.1544 | 0.9891 | 0.8106 | 0.9905 | 0.9638 | 0.3974 | 0.6772 | 0.6963 | 0.9585 |
| Large intestine | 0.9702 | 0.2503 | 0.9139 | 0.9496 | 0.867 | 0.2128 | 0.8577 | 0.7903 | 0.8355 |
| Liver (E14) | 0.832 | 0.5578 | 0.2599 | 0.6964 | 0.7963 | 0.1165 | 0.5545 | 0.8013 | 0.3728 |
| Liver (E14.5) | 0.8409 | 0.579 | 0.2107 | 0.6216 | 0.9072 | 0.1355 | 0.3381 | 0.6608 | 0.4154 |
| Lower molar | 0.7923 | 0.9663 | 0.8071 | 0.7914 | 0.8441 | 0.4099 | 0.7309 | 0.4884 | 0.8866 |
| Lung | 0.4173 | 0.0744 | 0.1611 | 0.225 | 0.1376 | 0.3958 | 0.4531 | 0.6382 | 0.0727 |
| Mammary tissue | 0.9128 | 0.1747 | 0.2201 | 0.7375 | 0.894 | 0.5577 | 0.7286 | 0.8057 | 0.6922 |
| Mature sperm | 0.9838 | 0.4742 | 0.5384 | 0.9821 | 0.9643 | 0.8048 | 0.9559 | 0.9727 | 0.9161 |
| Medullary thymic epithelial | 0.0178 | 0.5321 | 0.7918 | 0.9123 | 0.8929 | 0.1826 | 0.3831 | 0.8459 | 0.1455 |
| MEF | 0.6251 | 0.9584 | 0.9222 | 0.9818 | 0.937 | 0.7681 | 0.83 | 0.9607 | 0.9516 |
| Megakaryocyte erythroid progenitor | 0.7275 | 0.5289 | 0.472 | 0.5648 | 0.4886 | 0.5722 | 0.2595 | 0.4607 | 0.486 |
| Mesodermal cells | 0.9182 | 0.8347 | 0.9952 | 0.1972 | 0.9633 | 0.4029 | 0.957 | 0.8369 | 0.9509 |
| Morula | 0.9382 | 0.7946 | 0.926 | 0.9895 | 0.5586 | 0.9537 | 0.9566 | 0.9699 | 0.9516 |
| Motor neurons | 0.8977 | 0.9331 | 0.7994 | 0.759 | 0.9574 | 0.2786 | 0.8235 | 0.7924 | 0.9515 |
| Myelocytes | 0.0415 | 0.6114 | 0.1863 | 0.0312 | 0.5527 | 0.2087 | 0.1555 | 0.9572 | 0.2013 |
| Neocortex cortical plate | 0.7756 | 0.8091 | 0.976 | 0.6887 | 0.9643 | 0.0895 | 0.9481 | 0.7145 | 0.9514 |
| Neocortex subventricular zone | 0.7865 | 0.94 | 0.8604 | 0.5845 | 0.8488 | 0.0932 | 0.803 | 0.584 | 0.9509 |
| Neocortex ventricular zone | 0.8121 | 0.9676 | 0.9143 | 0.6137 | 0.9582 | 0.4727 | 0.8915 | 0.431 | 0.9503 |
| Neutrophil -IL10 -LPS | 0.0398 | 0.4463 | 0.1472 | 0.0421 | 0.1085 | 0.1125 | 0.219 | 0.5922 | 0.0204 |
| Neutrophil -IL10 +LPS | 0.0434 | 0.4606 | 0.2167 | 0.0987 | 0.2442 | 0.1969 | 0.3074 | 0.3464 | 0.0418 |
| Neutrophil +IL10 -LPS | 0.0345 | 0.4444 | 0.1872 | 0.0558 | 0.0863 | 0.1343 | 0.2303 | 0.5784 | 0.0223 |
| Neuron cell NCAM+ | 0.956 | 0.2789 | 0.9464 | 0.5129 | 0.9674 | 0.4906 | 0.9678 | 0.9559 | 0.9562 |
| Neural progenitor cell | 0.8504 | 0.9863 | 0.996 | 0.6506 | 0.7589 | 0.6836 | 0.8386 | 0.6493 | 0.9552 |
| Orthochromatic erythroblast | 0.5937 | 0.793 | 0.8143 | 0.0736 | 0.3818 | 0.0982 | 0.6101 | 0.1671 | 0.3152 |
| Oviduct | 0.0269 | 0.303 | 0.7752 | 0.5347 | 0.9647 | 0.1629 | 0.8851 | 0.9238 | 0.8862 |
| Embryonic kidney fibroblast | 0.9858 | 0.7477 | 0.6464 | 0.6487 | 0.2768 | 0.1508 | 0.6703 | 0.4045 | 0.1461 |
| Primordial germ cells | 0.9919 | 0.7945 | 0.9927 | 0.8269 | 0.7332 | 0.6177 | 0.967 | 0.9436 | 0.906 |
| Polychromatic erythroblast | 0.5454 | 0.6961 | 0.7086 | 0.0538 | 0.3878 | 0.0991 | 0.5174 | 0.3005 | 0.1258 |
| Spermatogonia (primitive type A) | 0.9831 | 0.9533 | 0.9634 | 0.7676 | 0.9652 | 0.9661 | 0.9152 | 0.8403 | 0.9562 |
| pro-B cells | 0.5468 | 0.042 | 0.3258 | 0.2167 | 0.1367 | 0.1515 | 0.105 | 0.196 | 0.0049 |
| pro-B (fraction B) | 0.6559 | 0.0263 | 0.7721 | 0.0403 | 0.0271 | 0.1168 | 0.1077 | 0.4518 | 0.0052 |
| pro-B (fraction CC') | 0.7021 | 0.0287 | 0.7819 | 0.046 | 0.0152 | 0.0759 | 0.1264 | 0.4848 | 0.0073 |
| Proerythroblast | 0.6272 | 0.7913 | 0.8487 | 0.1075 | 0.6213 | 0.1227 | 0.6593 | 0.5729 | 0.4253 |
| Prostate basal cells | 0.97 | 0.0333 | 0.723 | 0.9244 | 0.784 | 0.1229 | 0.7829 | 0.804 | 0.7882 |
| Retina | 0.91 | 0.93 | 0.9069 | 0.9908 | 0.9189 | 0.8412 | 0.9665 | 0.8548 | 0.9587 |
| Skeletal muscle | 0.9745 | 0.6156 | 0.5971 | 0.8553 | 0.8715 | 0.3634 | 0.8056 | 0.5637 | 0.9021 |
| Skin epithelial | 0.6476 | 0.4878 | 0.971 | 0.4396 | 0.9202 | 0.4329 | 0.8785 | 0.7014 | 0.9613 |
| Striatal neurons | 0.9636 | 0.9877 | 0.9842 | 0.9071 | 0.8542 | 0.7894 | 0.9327 | 0.6211 | 0.9631 |
| Striated muscle | 0.9762 | 0.5333 | 0.4775 | 0.7303 | 0.7284 | 0.5081 | 0.8583 | 0.7476 | 0.5796 |
| Substantia nigra | 0.8378 | 0.9527 | 0.614 | 0.9913 | 0.8621 | 0.8716 | 0.9576 | 0.7875 | 0.7495 |
| Telencephalon | 0.7002 | 0.973 | 0.9863 | 0.2202 | 0.9567 | 0.1542 | 0.9189 | 0.8736 | 0.961 |
| Erythroid cells (TER119+) | 0.6298 | 0.7858 | 0.5923 | 0.0869 | 0.2854 | 0.0734 | 0.5622 | 0.2254 | 0.2462 |
| Thymus | 0.6431 | 0.093 | 0.4583 | 0.0473 | 0.3229 | 0.1773 | 0.5723 | 0.0097 | 0.1398 |
| Upper molar | 0.9141 | 0.9652 | 0.8358 | 0.8523 | 0.826 | 0.4446 | 0.8381 | 0.3189 | 0.961 |
| Uterus | 0.9183 | 0.0844 | 0.6413 | 0.7856 | 0.9382 | 0.2066 | 0.8526 | 0.7621 | 0.8839 |
| Vas deferens | 0.981 | 0.0655 | 0.4554 | 0.9545 | 0.7033 | 0.2536 | 0.8213 | 0.8634 | 0.4458 |
| Ventral tagmental | 0.475 | 0.7469 | 0.6773 | 0.9884 | 0.5381 | 0.8976 | 0.9263 | 0.4684 | 0.96 |
| Whole brain (E14.5) | 0.7737 | 0.9183 | 0.6867 | 0.5211 | 0.9375 | 0.101 | 0.9025 | 0.2812 | 0.9592 |
| Whole brain (E18.5) | 0.9386 | 0.8974 | 0.6959 | 0.4786 | 0.9641 | 0.1024 | 0.9205 | 0.3321 | 0.959 |
| 4C Embryo | 0.6303 | 0.6777 | 0.9949 | 0.9909 | 0.3896 | 0.7439 | 0.9665 | 0.9544 | 0.2232 |
| 8C Embryo | 0.9849 | 0.8176 | 0.9949 | 0.9907 | 0.3043 | 0.8537 | 0.9658 | 0.385 | 0.9596 |
| B cell (germinal center) | 0.9909 | 0.7193 | 0.9954 | 0.2831 | 0.0097 | 0.1796 | 0.3011 | 0.2795 | 0.0071 |
| B cell (resting) | 0.9783 | 0.0277 | 0.9934 | 0.1114 | 0.0065 | 0.2119 | 0.336 | 0.583 | 0.0092 |
| B cell (active) | 0.9849 | 0.1256 | 0.9999 | 0.8414 | 0.0122 | 0.7038 | 0.6093 | 0.6529 | 0.0092 |
| B cells CD43- | 0.9859 | 0.0311 | 0.971 | 0.2237 | 0.0042 | 0.2047 | 0.2647 | 0.1009 | 0.008 |
| B cells CD19+ | 0.9946 | 0.0311 | 0.8587 | 0.3098 | 0.0042 | 0.2334 | 0.2172 | 0.155 | 0.005 |
| B cell (naive) | 0.9917 | 0.0314 | 0.997 | 0.5363 | 0.0025 | 0.3339 | 0.2982 | 0.6254 | 0.0042 |
| Blastocyst (Early) TE | 0.9943 | 0.8678 | 0.9022 | 0.993 | 0.2653 | 0.9857 | 0.9744 | 0.9031 | 0.4477 |
| Blastocyst (Late) TE | 0.7006 | 0.7958 | 0.9093 | 0.9901 | 0.5548 | 0.9869 | 0.8229 | 0.929 | 0.3853 |
| pre-B cells | 0.9365 | 0.0438 | 0.8222 | 0.4636 | 0.022 | 0.0996 | 0.2829 | 0.4347 | 0.001 |
| Spleen | 0.4444 | 0.0943 | 0.0773 | 0.0759 | 0.0036 | 0.2376 | 0.0207 | 0.0415 | 0.0048 |
| splenic B cells | 0.7753 | 0.306 | 0.9895 | 0.5446 | 0.0215 | 0.5213 | 0.019 | 0.9559 | 0.0052 |

| **Supplementary Table 9.**  Difference score for each TF selected by MIMIC in an extended dataset | | | | | | | | |
| --- | --- | --- | --- | --- | --- | --- | --- | --- |
| Cell type | Cebpa | Nfib | Bcl11a | Zfp57 | Enpp2 | Peg3 | Tcf7 | Notch3 |
| B cell (germinal center) | 0.9796 | 0.9056 | 0.2153 | 0.8603 | 0.5559 | 0.9470 | 0.0456 | 0.9480 |
| B cell (resting) | 0.9836 | 0.8967 | 0.1517 | 0.8140 | 0.6778 | 0.9811 | 0.0764 | 0.8940 |
| B cell (active) | 0.9972 | 0.9344 | 0.2058 | 0.2570 | 0.8636 | 0.9532 | 0.1972 | 0.7210 |
| Basophilic erythroblast | 0.8737 | 0.9450 | 0.6802 | 0.8603 | 0.8990 | 0.9811 | 0.7039 | 0.9480 |
| B cells CD43- | 0.9714 | 0.2902 | 0.0723 | 0.8603 | 0.4029 | 0.9297 | 0.3273 | 0.8014 |
| B cells CD19+ | 0.9668 | 0.7694 | 0.1009 | 0.8603 | 0.3392 | 0.9811 | 0.0090 | 0.9480 |
| Bone marrow | 0.7881 | 0.1180 | 0.3549 | 0.1244 | 0.7342 | 0.5233 | 0.3273 | 0.0200 |
| B cell (naive) | 0.9972 | 0.8967 | 0.1053 | 0.8603 | 0.8636 | 0.9772 | 0.4427 | 0.9480 |
| DC CD11+ | 0.3325 | 0.9622 | 0.1288 | 0.3920 | 0.6591 | 0.9917 | 0.3797 | 0.9452 |
| DC CD8+ | 0.4829 | 0.9884 | 0.1647 | 0.2160 | 0.8695 | 0.9837 | 0.5726 | 0.1810 |
| Epidermis (E16.5) | 0.0628 | 0.2962 | 0.5962 | 0.7966 | 0.5265 | 0.7541 | 0.9292 | 0.0709 |
| Erythroblast | 0.7484 | 0.8882 | 0.2809 | 0.9128 | 0.9484 | 0.8207 | 0.9808 | 0.7937 |
| Erythroid progenitor | 0.4581 | 0.9133 | 0.2039 | 0.9271 | 0.9667 | 0.9112 | 0.9210 | 0.9052 |
| Hematopoietic stem cells | 0.6009 | 0.9627 | 0.2315 | 0.8041 | 0.8411 | 0.9836 | 0.8211 | 0.9200 |
| Mature sperm | 0.8391 | 0.7173 | 0.5195 | 0.6307 | 0.8687 | 0.9960 | 0.9930 | 0.8539 |
| Megakaryoblast | 0.3403 | 0.8819 | 0.5973 | 0.6482 | 0.9842 | 0.9444 | 0.9876 | 0.3689 |
| Orthochromatic erythroblast | 0.7486 | 0.9368 | 0.7465 | 0.9828 | 0.9301 | 0.9988 | 0.9848 | 0.9936 |
| pre-B cells | 0.8923 | 0.9965 | 0.4717 | 0.9902 | 0.9835 | 0.9987 | 0.6877 | 0.9657 |
| pro-B cells | 0.6308 | 0.9859 | 0.1559 | 0.9896 | 0.9780 | 0.9965 | 0.4192 | 0.8585 |
| pro-B (fraction B) | 0.8692 | 0.9811 | 0.1302 | 0.9850 | 0.9843 | 0.9987 | 0.6092 | 0.9028 |
| pro-B (fraction CC') | 0.8759 | 0.9908 | 0.1838 | 0.9894 | 0.9842 | 0.9987 | 0.6451 | 0.9439 |
| Proerythroblast | 0.8438 | 0.9965 | 0.3248 | 0.9893 | 0.9842 | 0.9987 | 0.9784 | 0.9935 |
| Spermatocytes | 0.9839 | 0.8977 | 0.6803 | 0.7398 | 0.8045 | 0.9271 | 0.7748 | 0.9829 |
| splenic B cells | 0.9938 | 0.9948 | 0.1689 | 0.9725 | 0.9843 | 0.9977 | 0.8809 | 0.9900 |
| Erythroid cells (TER119+) | 0.9217 | 0.9935 | 0.3571 | 0.9880 | 0.9156 | 0.9955 | 0.9743 | 0.9223 |
| Adipocytes | 0.0925 | 0.0073 | 0.9963 | 0.1975 | 0.0049 | 0.0037 | 0.7039 | 0.0055 |
| Adipocytes (cultured, brown) | 0.1257 | 0.0054 | 0.9963 | 0.1636 | 0.0022 | 0.0090 | 0.7039 | 0.0035 |
| Adipose | 0.0998 | 0.0056 | 0.9904 | 0.8603 | 0.0031 | 0.0473 | 0.7039 | 0.0084 |
| Blastocyst | 0.3565 | 0.9450 | 0.9963 | 0.0098 | 0.8990 | 0.0312 | 0.4133 | 0.8785 |
| Monocyte | 0.2122 | 0.8619 | 0.2546 | 0.8603 | 0.7078 | 0.9490 | 0.4133 | 0.9222 |
| BMDM | 0.3692 | 0.6281 | 0.9779 | 0.8140 | 0.5453 | 0.9410 | 0.4770 | 0.9105 |
| BMDM +CpolyG | 0.5220 | 0.5645 | 0.9712 | 0.6100 | 0.3178 | 0.9811 | 0.4133 | 0.8247 |
| BMDM +IL-1b | 0.4837 | 0.6092 | 0.9877 | 0.6549 | 0.3178 | 0.9663 | 0.2815 | 0.8247 |
| BMDM -lipidA | 0.2352 | 0.9450 | 0.9827 | 0.8603 | 0.8990 | 0.9811 | 0.7039 | 0.9480 |
| BMDM +lipidA 20 mins | 0.6092 | 0.7487 | 0.9877 | 0.8603 | 0.8990 | 0.9811 | 0.7039 | 0.9480 |
| BMDM +lipidA 30 mins | 0.2909 | 0.9450 | 0.9628 | 0.8603 | 0.8990 | 0.9811 | 0.7039 | 0.9105 |
| BMDM +lipidA 360 mins | 0.7670 | 0.9450 | 0.9779 | 0.8603 | 0.4983 | 0.9663 | 0.7039 | 0.9480 |
| BMDM +lipidA 60 mins | 0.4333 | 0.8721 | 0.9779 | 0.8603 | 0.8990 | 0.9811 | 0.7039 | 0.9480 |
| BMDM +MALP2 | 0.6099 | 0.5645 | 0.9851 | 0.8603 | 0.3178 | 0.9811 | 0.4770 | 0.7597 |
| BMDM +IFNg | 0.5757 | 0.6092 | 0.9851 | 0.7084 | 0.2327 | 0.9532 | 0.4133 | 0.7798 |
| BMDM +IL-4 | 0.2909 | 0.5859 | 0.9779 | 0.6100 | 0.3552 | 0.9811 | 0.3651 | 0.8014 |
| BMDM +IFNa | 0.4846 | 0.5859 | 0.9827 | 0.7743 | 0.2986 | 0.9663 | 0.4133 | 0.8503 |
| BMDM +TGFb | 0.2092 | 0.5750 | 0.9802 | 0.6549 | 0.4142 | 0.9811 | 0.3273 | 0.8503 |
| Dendritic cells (BMDC) | 0.1715 | 0.8720 | 0.9447 | 0.8413 | 0.5328 | 0.9902 | 0.9537 | 0.9463 |
| 8C Embryo (single-cell) | 0.2548 | 0.7588 | 0.9921 | 0.3169 | 0.5585 | 0.9309 | 0.8613 | 0.9743 |
| Eosinophils -IL-10 -LPS | 0.6385 | 0.9926 | 0.9390 | 0.8284 | 0.9667 | 0.9991 | 0.9092 | 0.9167 |
| Eosinophils -IL-10 +LPS | 0.6237 | 0.9926 | 0.9362 | 0.8331 | 0.9080 | 0.9991 | 0.9201 | 0.9046 |
| Granulocytes | 0.2141 | 0.9936 | 0.7771 | 0.9923 | 0.3024 | 0.9988 | 0.9843 | 0.9957 |
| Blastocyst (Late) TE | 0.1198 | 0.8045 | 0.9966 | 0.2635 | 0.9748 | 0.8630 | 0.9938 | 0.9946 |
| Liver | 0.0387 | 0.1626 | 0.9675 | 0.9689 | 0.0379 | 0.8135 | 0.8662 | 0.8711 |
| Macrophages -IL10 -LPS | 0.0665 | 0.8434 | 0.9237 | 0.9020 | 0.3224 | 0.9961 | 0.9416 | 0.9949 |
| Macrophages -IL10 +LPS | 0.3841 | 0.7382 | 0.9133 | 0.8830 | 0.2001 | 0.9965 | 0.8833 | 0.9567 |
| Macrophages +IL10 -LPS | 0.0795 | 0.8506 | 0.9286 | 0.9284 | 0.3077 | 0.9939 | 0.9207 | 0.9842 |
| Macrophages +IL10 +LPS | 0.3857 | 0.7724 | 0.9181 | 0.9007 | 0.2325 | 0.9912 | 0.9057 | 0.9821 |
| Mammary tissue | 0.0389 | 0.1492 | 0.9575 | 0.9757 | 0.0190 | 0.7839 | 0.9564 | 0.3530 |
| Medullary thymic epithelial | 0.2202 | 0.6623 | 0.8407 | 0.9690 | 0.1817 | 0.5945 | 0.2876 | 0.7122 |
| Megakaryocyte erythroid progenitor | 0.1013 | 0.9754 | 0.1873 | 0.9156 | 0.9842 | 0.9965 | 0.7014 | 0.9230 |
| Microglia | 0.0643 | 0.9609 | 0.8610 | 0.7628 | 0.2456 | 0.9879 | 0.9781 | 0.9946 |
| Morula (single-cell) | 0.4463 | 0.9424 | 0.9833 | 0.3960 | 0.9834 | 0.9215 | 0.6800 | 0.9502 |
| Morula | 0.5698 | 0.9328 | 0.9957 | 0.6831 | 0.9348 | 0.9100 | 0.7909 | 0.9942 |
| Mast cell -IL10 -LPS | 0.5098 | 0.9963 | 0.8267 | 0.8371 | 0.9842 | 0.9983 | 0.9383 | 0.9264 |
| Mast cell -IL10 +LPS | 0.5452 | 0.9962 | 0.6910 | 0.7837 | 0.9643 | 0.9988 | 0.9276 | 0.8787 |
| Mast cell +IL10 -LPS | 0.4892 | 0.9961 | 0.8523 | 0.8589 | 0.9689 | 0.9988 | 0.9239 | 0.9110 |
| Mast cell +IL10 +LPS | 0.4886 | 0.9927 | 0.7463 | 0.7800 | 0.9545 | 0.9988 | 0.9324 | 0.8361 |
| Myelocytes | 0.3867 | 0.9959 | 0.6951 | 0.9827 | 0.5722 | 0.9988 | 0.9818 | 0.9938 |
| Myeloid cells | 0.1480 | 0.8692 | 0.8916 | 0.9803 | 0.1570 | 0.9988 | 0.9339 | 0.8927 |
| Neutrophil -IL10 -LPS | 0.2096 | 0.9963 | 0.7012 | 0.9462 | 0.9058 | 0.9987 | 0.7708 | 0.9834 |
| Neutrophil -IL10 +LPS | 0.1929 | 0.9962 | 0.5216 | 0.9306 | 0.9317 | 0.9987 | 0.7618 | 0.9703 |
| Neutrophil +IL10 -LPS | 0.2421 | 0.9961 | 0.6359 | 0.9432 | 0.9184 | 0.9981 | 0.7386 | 0.9834 |
| Neutrophil +IL10 +LPS | 0.2566 | 0.9960 | 0.5282 | 0.9253 | 0.9350 | 0.9987 | 0.7535 | 0.9811 |
| Polychromatic erythroblast | 0.6393 | 0.9963 | 0.6705 | 0.9904 | 0.9842 | 0.9984 | 0.9702 | 0.9934 |
| pre-Adipocytes | 0.0774 | 0.2411 | 0.9790 | 0.6581 | 0.5033 | 0.5636 | 0.9772 | 0.1918 |
| Adipocytes (brown) | 0.0303 | 0.1666 | 0.9852 | 0.8808 | 0.0447 | 0.7368 | 0.9772 | 0.1334 |
| Adipocytes (white) | 0.0384 | 0.1365 | 0.9313 | 0.9108 | 0.0293 | 0.6226 | 0.9638 | 0.0292 |
| Promyelocytes | 0.1045 | 0.9935 | 0.4533 | 0.9173 | 0.9605 | 0.9986 | 0.9122 | 0.9698 |
| Sperm | 0.1745 | 0.5147 | 0.9710 | 0.9726 | 0.4537 | 0.9510 | 0.8860 | 0.7075 |
| Spermatogonia | 0.3080 | 0.9763 | 0.6484 | 0.8103 | 0.8472 | 0.8867 | 0.6934 | 0.9929 |
| 4C Embryo | 0.1395 | 0.8507 | 0.9945 | 0.4658 | 0.9868 | 0.9564 | 0.7396 | 0.9421 |
| 8C Embryo | 0.1512 | 0.8222 | 0.9944 | 0.5988 | 0.9866 | 0.9879 | 0.5873 | 0.9150 |
| Caecum (E13.5) | 0.9386 | 0.0054 | 0.4320 | 0.0078 | 0.1080 | 0.0004 | 0.0260 | 0.0012 |
| Cardiac precursors | 0.9972 | 0.1862 | 0.9933 | 0.0049 | 0.8990 | 0.0004 | 0.0098 | 0.0010 |
| Cardiomyocytes | 0.9960 | 0.0536 | 0.9022 | 0.0099 | 0.8305 | 0.0037 | 0.0250 | 0.0079 |
| Cerebellum | 0.9748 | 0.0019 | 0.2113 | 0.2257 | 0.0027 | 0.0004 | 0.9958 | 0.0880 |
| Cortex | 0.9636 | 0.0114 | 0.0717 | 0.0989 | 0.0063 | 0.0034 | 0.9947 | 0.3057 |
| Cortex neuron | 0.9679 | 0.0096 | 0.0695 | 0.0525 | 0.0383 | 0.0160 | 0.9775 | 0.4330 |
| Dermal fibroblast | 0.4276 | 0.1034 | 0.9473 | 0.0949 | 0.5536 | 0.0463 | 0.9709 | 0.0617 |
| Dorsal rootganglia | 0.7871 | 0.2170 | 0.8939 | 0.4526 | 0.4823 | 0.0487 | 0.9931 | 0.1169 |
| Central neural epithelium (E10.5) | 0.9936 | 0.2693 | 0.1808 | 0.0114 | 0.3263 | 0.0305 | 0.9130 | 0.0144 |
| Dorsal control neural epithelium (E10.5) | 0.9935 | 0.2345 | 0.1732 | 0.0077 | 0.4888 | 0.0126 | 0.8182 | 0.0158 |
| Forelimb (E10.5) | 0.9480 | 0.6255 | 0.4865 | 0.0343 | 0.1382 | 0.0170 | 0.7030 | 0.0181 |
| Hindlimb (E10.5) | 0.9527 | 0.7560 | 0.6453 | 0.0549 | 0.2406 | 0.0298 | 0.5560 | 0.0341 |
| Lateral prominence neural epithelium (E10.5) | 0.9931 | 0.5365 | 0.1883 | 0.0342 | 0.5073 | 0.0845 | 0.9584 | 0.0686 |
| Mandibular arch (E10.5) | 0.9929 | 0.2444 | 0.1363 | 0.0180 | 0.0486 | 0.0635 | 0.8335 | 0.1542 |
| Maxillary arch (E10.5) | 0.9927 | 0.2677 | 0.1260 | 0.0246 | 0.0660 | 0.0889 | 0.7132 | 0.1257 |
| Medial nasal prominence (E10.5) | 0.9924 | 0.3824 | 0.3413 | 0.0405 | 0.0138 | 0.1484 | 0.9939 | 0.1179 |
| Dermis (E16.5) | 0.1416 | 0.1241 | 0.3013 | 0.3651 | 0.0430 | 0.2073 | 0.6233 | 0.0630 |
| Skin (E16.5) | 0.1472 | 0.0946 | 0.5491 | 0.1749 | 0.0390 | 0.1625 | 0.7698 | 0.1047 |
| Caudal brain neural epithelium (E8.5) | 0.9936 | 0.9827 | 0.9907 | 0.1995 | 0.1317 | 0.3592 | 0.4092 | 0.2041 |
| Non-floor plate neural epithelium (E8.5) | 0.9935 | 0.9827 | 0.9972 | 0.2928 | 0.9395 | 0.3446 | 0.6074 | 0.1885 |
| Cranial mesenchyme (E9.5) | 0.9879 | 0.7687 | 0.9789 | 0.0911 | 0.2839 | 0.2250 | 0.5812 | 0.4116 |
| Epidermal ectoderm (E9.5) | 0.9876 | 0.4920 | 0.6592 | 0.1985 | 0.3924 | 0.1282 | 0.3406 | 0.1351 |
| Mandibular arch (E9.5) | 0.9931 | 0.6948 | 0.8900 | 0.0750 | 0.5092 | 0.0884 | 0.4115 | 0.2398 |
| Maxillary arch (E9.5) | 0.9929 | 0.6275 | 0.5821 | 0.0966 | 0.2283 | 0.1012 | 0.7008 | 0.2309 |
| EpiSC | 0.9552 | 0.7978 | 0.8070 | 0.2848 | 0.0430 | 0.2538 | 0.2411 | 0.1326 |
| Embryonic stem cells (V6.5) | 0.9351 | 0.6656 | 0.9289 | 0.1446 | 0.0792 | 0.4668 | 0.2214 | 0.0592 |
| Ileum | 0.6663 | 0.1538 | 0.4854 | 0.3348 | 0.5581 | 0.1804 | 0.7259 | 0.1487 |
| Intestinal subepithelial myofibroblasts (adult) | 0.8131 | 0.2930 | 0.9795 | 0.3550 | 0.9095 | 0.4426 | 0.9308 | 0.9249 |
| Intestinal subepithelial myofibroblasts (neonatal) | 0.9728 | 0.2642 | 0.9966 | 0.3354 | 0.8073 | 0.3048 | 0.8803 | 0.4359 |
| Liver (E14) | 0.4323 | 0.6131 | 0.2393 | 0.8424 | 0.4342 | 0.2317 | 0.8904 | 0.9109 |
| Liver (E14.5) | 0.2266 | 0.4904 | 0.2426 | 0.8254 | 0.5401 | 0.2270 | 0.8340 | 0.9000 |
| Lower molar | 0.9737 | 0.0440 | 0.1122 | 0.3513 | 0.1381 | 0.0805 | 0.2474 | 0.1096 |
| MEF | 0.8816 | 0.2213 | 0.8614 | 0.3084 | 0.6950 | 0.2052 | 0.9461 | 0.2700 |
| Mesodermal cells | 0.9771 | 0.9561 | 0.9162 | 0.2932 | 0.7588 | 0.0558 | 0.2273 | 0.0821 |
| Motor neurons | 0.9576 | 0.1988 | 0.8394 | 0.6983 | 0.1414 | 0.2505 | 0.9420 | 0.6889 |
| Myoblast | 0.6901 | 0.4271 | 0.9748 | 0.4374 | 0.9199 | 0.5380 | 0.8752 | 0.5953 |
| Neuron cell NCAM+ | 0.9862 | 0.5100 | 0.7139 | 0.1426 | 0.1736 | 0.0792 | 0.8441 | 0.2462 |
| Nucleus accumbens | 0.8866 | 0.5606 | 0.2353 | 0.8997 | 0.0397 | 0.1942 | 0.8602 | 0.4252 |
| Olfactory bulb | 0.9210 | 0.4530 | 0.1159 | 0.6384 | 0.0581 | 0.2641 | 0.8731 | 0.5784 |
| Embryonic kidney fibroblast | 0.7140 | 0.1295 | 0.8664 | 0.6786 | 0.9310 | 0.2163 | 0.7226 | 0.9014 |
| Primordial germ cells | 0.9929 | 0.9515 | 0.9038 | 0.8197 | 0.7884 | 0.5936 | 0.2268 | 0.9715 |
| Placenta | 0.3838 | 0.6727 | 0.9907 | 0.8569 | 0.3680 | 0.0833 | 0.8215 | 0.6568 |
| Retina | 0.9903 | 0.5681 | 0.8419 | 0.8902 | 0.1792 | 0.1446 | 0.9811 | 0.8980 |
| Striated muscle | 0.6903 | 0.1089 | 0.9819 | 0.9125 | 0.6269 | 0.1502 | 0.8932 | 0.2660 |
| Striatum | 0.8363 | 0.6559 | 0.3524 | 0.9285 | 0.0407 | 0.1729 | 0.6275 | 0.5587 |
| Substantia nigra | 0.9935 | 0.8402 | 0.7013 | 0.3482 | 0.0355 | 0.1034 | 0.9575 | 0.8046 |
| Subventricular zone | 0.8225 | 0.3563 | 0.2995 | 0.5110 | 0.0534 | 0.1859 | 0.7645 | 0.6287 |
| Polyploid trophoblast giant cells (E9.5) | 0.9457 | 0.9260 | 0.9898 | 0.7157 | 0.9720 | 0.2169 | 0.2927 | 0.9867 |
| Trophoblast stem cells | 0.6865 | 0.9828 | 0.9855 | 0.3085 | 0.9607 | 0.7327 | 0.6752 | 0.7461 |
| Upper molar | 0.8862 | 0.0483 | 0.1115 | 0.2845 | 0.1317 | 0.0685 | 0.1616 | 0.0914 |
| Ventral tagmental | 0.9919 | 0.7558 | 0.3916 | 0.1922 | 0.0689 | 0.0735 | 0.8397 | 0.7902 |
| Cerebellar granular neurons | 0.8811 | 0.0001 | 0.4234 | 0.0034 | 0.0406 | 0.0005 | 0.9902 | 0.0903 |
| Colon | 0.5028 | 0.0158 | 0.6624 | 0.5298 | 0.0787 | 0.5014 | 0.9206 | 0.2198 |
| Cortical thymic epithelial | 0.3653 | 0.0092 | 0.2113 | 0.0298 | 0.0835 | 0.1756 | 0.8682 | 0.2502 |
| Dentate gyrus | 0.6805 | 0.0207 | 0.3653 | 0.1130 | 0.0394 | 0.0651 | 0.9894 | 0.3516 |
| Cortical neurons (E16.5) | 0.9581 | 0.0420 | 0.1583 | 0.0157 | 0.4387 | 0.5887 | 0.9883 | 0.6793 |
| Basal epidermis (E14.5) | 0.2444 | 0.0216 | 0.1227 | 0.4180 | 0.9134 | 0.3913 | 0.5677 | 0.5481 |
| Fatpad | 0.0461 | 0.0283 | 0.4410 | 0.9932 | 0.2956 | 0.3985 | 0.9818 | 0.3792 |
| Genital fatpad | 0.0503 | 0.0423 | 0.4070 | 0.9926 | 0.4345 | 0.4801 | 0.4551 | 0.5751 |
| Hair follicle | 0.1778 | 0.0101 | 0.2443 | 0.8472 | 0.1106 | 0.3308 | 0.7464 | 0.6690 |
| Heart | 0.8092 | 0.0711 | 0.9513 | 0.8478 | 0.1286 | 0.7400 | 0.9880 | 0.4050 |
| Hippocampus neuron | 0.8930 | 0.0695 | 0.1488 | 0.7730 | 0.2481 | 0.2466 | 0.9079 | 0.9633 |
| Hippocampus tissue | 0.9506 | 0.0253 | 0.1205 | 0.6926 | 0.1319 | 0.3191 | 0.9750 | 0.8658 |
| Intestine | 0.3451 | 0.3137 | 0.6765 | 0.9771 | 0.5000 | 0.9309 | 0.8272 | 0.9294 |
| Keratinocyte | 0.4404 | 0.2482 | 0.9966 | 0.9109 | 0.9745 | 0.6895 | 0.9595 | 0.8137 |
| Large intestine | 0.3661 | 0.2405 | 0.8550 | 0.9841 | 0.7011 | 0.9208 | 0.9079 | 0.9897 |
| Lung | 0.1399 | 0.0803 | 0.9605 | 0.5118 | 0.0769 | 0.8721 | 0.6190 | 0.1836 |
| Neocortex cortical plate | 0.9929 | 0.0072 | 0.0304 | 0.0562 | 0.6936 | 0.5835 | 0.9573 | 0.7289 |
| Neocortex subventricular zone | 0.9848 | 0.0103 | 0.0473 | 0.0686 | 0.6338 | 0.6324 | 0.9042 | 0.7903 |
| Neocortex ventricular zone | 0.9765 | 0.0398 | 0.1764 | 0.2670 | 0.8122 | 0.5504 | 0.8723 | 0.0997 |
| Neural progenitor cell | 0.7548 | 0.0352 | 0.8997 | 0.9515 | 0.8745 | 0.3117 | 0.7648 | 0.2060 |
| Oviduct | 0.4335 | 0.1533 | 0.9368 | 0.9026 | 0.0532 | 0.9552 | 0.9044 | 0.3474 |
| pre-Adipocytes (brown) | 0.1133 | 0.1756 | 0.9786 | 0.4684 | 0.3142 | 0.6356 | 0.9907 | 0.3516 |
| Prostate basal cells | 0.7693 | 0.1197 | 0.8869 | 0.9719 | 0.7291 | 0.9742 | 0.4351 | 0.9021 |
| Skeletal muscle | 0.3942 | 0.1253 | 0.9908 | 0.9488 | 0.1862 | 0.6918 | 0.9674 | 0.3221 |
| Skin epithelial | 0.0857 | 0.0479 | 0.1040 | 0.9519 | 0.9406 | 0.3276 | 0.5569 | 0.2154 |
| Striatal neurons | 0.8730 | 0.1496 | 0.9946 | 0.7415 | 0.8560 | 0.9957 | 0.1821 | 0.5014 |
| tail fibroblast | 0.4652 | 0.5622 | 0.9401 | 0.6411 | 0.6327 | 0.9483 | 0.9419 | 0.5926 |
| Telencephalon | 0.9931 | 0.0859 | 0.0510 | 0.0253 | 0.8365 | 0.2272 | 0.9506 | 0.4546 |
| Uterus | 0.4988 | 0.2441 | 0.9739 | 0.5914 | 0.2166 | 0.9732 | 0.5356 | 0.6994 |
| Whole brain (E14.5) | 0.9681 | 0.0249 | 0.0489 | 0.0457 | 0.4125 | 0.1972 | 0.8185 | 0.2522 |
| Whole brain (E18.5) | 0.9786 | 0.0366 | 0.0598 | 0.0378 | 0.2692 | 0.2166 | 0.9023 | 0.4959 |
| CD4+ iTreg cells | 0.9972 | 0.7734 | 0.9676 | 0.7548 | 0.8965 | 0.9879 | 0.0005 | 0.9687 |
| CD4+ T cells -IL-21 | 0.9924 | 0.9486 | 0.9671 | 0.8651 | 0.5279 | 0.9272 | 0.0004 | 0.7950 |
| Naive CD4+ T cells, anti-CD3/28 treated | 0.9970 | 0.9450 | 0.9921 | 0.7743 | 0.8965 | 0.9811 | 0.0010 | 0.8014 |
| CD4+ T cells +IL-21 | 0.9848 | 0.9409 | 0.9687 | 0.7666 | 0.4592 | 0.8624 | 0.0014 | 0.6612 |
| CD4+ Th17 cells | 0.9966 | 0.8909 | 0.9956 | 0.8493 | 0.2679 | 0.8955 | 0.0029 | 0.4448 |
| CD4+ Th1 cells | 0.9966 | 0.8248 | 0.9952 | 0.1143 | 0.3931 | 0.9405 | 0.0118 | 0.2350 |
| CD4+ Th2 cells | 0.9965 | 0.9144 | 0.9887 | 0.4706 | 0.3135 | 0.5564 | 0.0302 | 0.2466 |
| CD4+ Th9 cells | 0.9965 | 0.8958 | 0.9944 | 0.6448 | 0.6894 | 0.8971 | 0.0174 | 0.1525 |
| CD4+ Treg cells | 0.9935 | 0.8651 | 0.9939 | 0.4853 | 0.7743 | 0.8201 | 0.0200 | 0.4529 |
| CD4+ T cells (anti-CD3/CD28 treated) | 0.9268 | 0.8651 | 0.7574 | 0.8352 | 0.6894 | 0.8056 | 0.0317 | 0.8418 |
| CD4+ T cells | 0.9262 | 0.8651 | 0.7840 | 0.8179 | 0.8965 | 0.7881 | 0.0094 | 0.5645 |
| CD8+ T cells | 0.9189 | 0.8651 | 0.5647 | 0.7958 | 0.5965 | 0.7666 | 0.0816 | 0.3309 |
| CD8+ T cells +IL-2 | 0.9880 | 0.8651 | 0.5094 | 0.7666 | 0.8965 | 0.7395 | 0.3317 | 0.4151 |
| Double negative 1 (DN1) thymic cells | 0.7843 | 0.9596 | 0.1753 | 0.6831 | 0.8061 | 0.9034 | 0.0707 | 0.0142 |
| Double negative 2a (DN2a) thymic cells | 0.9766 | 0.9654 | 0.4092 | 0.8417 | 0.8647 | 0.9470 | 0.0528 | 0.0229 |
| Double negative 2b (DN2b) thymic cells | 0.9098 | 0.9240 | 0.7146 | 0.7205 | 0.7860 | 0.9562 | 0.0343 | 0.0030 |
| Double negative 3 (DN3) thymic cells | 0.9938 | 0.9787 | 0.7650 | 0.9152 | 0.8817 | 0.9882 | 0.0527 | 0.0022 |
| Double positive thymic cells | 0.9792 | 0.9916 | 0.9888 | 0.9048 | 0.9076 | 0.9934 | 0.0180 | 0.0635 |
| Epiblast | 0.9919 | 0.5638 | 0.8816 | 0.4497 | 0.9654 | 0.9683 | 0.4204 | 0.9314 |
| Innate lymphoid cell (Type 2) | 0.9919 | 0.9677 | 0.9888 | 0.8122 | 0.9758 | 0.9860 | 0.0878 | 0.9364 |
| Naive CD4+ CD25- T cells | 0.8092 | 0.9816 | 0.8245 | 0.9904 | 0.8026 | 0.9988 | 0.0033 | 0.9791 |
| Spermatogonia (primitive type A) | 0.6685 | 0.5277 | 0.9005 | 0.9042 | 0.1239 | 0.8251 | 0.0578 | 0.9111 |
| splenic DC -IL10 -LPS | 0.2278 | 0.9908 | 0.6674 | 0.9104 | 0.8172 | 0.9980 | 0.0231 | 0.9240 |
| splenic DC -IL10 +LPS | 0.6497 | 0.9832 | 0.7054 | 0.8906 | 0.7326 | 0.9980 | 0.0225 | 0.9155 |
| splenic DC +IL10 -LPS | 0.2669 | 0.9893 | 0.6400 | 0.9072 | 0.8194 | 0.9986 | 0.0266 | 0.9198 |
| splenic DC +IL10 +LPS | 0.5959 | 0.9854 | 0.6311 | 0.9173 | 0.6734 | 0.9980 | 0.0292 | 0.9277 |
| Spleen | 0.6606 | 0.6621 | 0.1254 | 0.9623 | 0.0801 | 0.9851 | 0.0592 | 0.5107 |
| Thymus | 0.6144 | 0.7750 | 0.7648 | 0.9785 | 0.3436 | 0.9872 | 0.0023 | 0.2832 |
| Spermatogonia (type A) | 0.7845 | 0.5413 | 0.9063 | 0.9145 | 0.1311 | 0.7995 | 0.1110 | 0.8922 |
| Blastocyst (Early) TE | 0.4640 | 0.8433 | 0.9878 | 0.0692 | 0.9673 | 0.9302 | 0.7987 | 0.9972 |
| Blastocyst (Early) ICM | 0.4560 | 0.9117 | 0.9967 | 0.0256 | 0.9667 | 0.9893 | 0.7630 | 0.9854 |
| Spermatids (elogated) | 0.8192 | 0.9727 | 0.7978 | 0.2411 | 0.8032 | 0.9865 | 0.7832 | 0.9968 |
| 2C Embyro (early) | 0.9919 | 0.8119 | 0.9895 | 0.0021 | 0.1569 | 0.8511 | 0.1563 | 0.9965 |
| 2C Embyro (late) | 0.6613 | 0.8244 | 0.9945 | 0.0224 | 0.5100 | 0.9049 | 0.3732 | 0.9522 |
| 2C Embyro (middle) | 0.6397 | 0.8688 | 0.9174 | 0.0396 | 0.2449 | 0.8038 | 0.2329 | 0.9793 |
| 4C Embryo (single-cell) | 0.2313 | 0.8910 | 0.9966 | 0.1978 | 0.9698 | 0.8859 | 0.6756 | 0.9223 |
| 2C Embryo | 0.7087 | 0.8364 | 0.9839 | 0.0598 | 0.8779 | 0.9059 | 0.3509 | 0.8855 |
| Embryonic stem cells (E14) | 0.9429 | 0.6503 | 0.9356 | 0.0568 | 0.5735 | 0.3888 | 0.2313 | 0.1546 |
| Naive ESC (Hex-) | 0.9822 | 0.4490 | 0.9966 | 0.0269 | 0.9654 | 0.7834 | 0.2223 | 0.4439 |
| Naive ESC (Hex+) | 0.9849 | 0.4763 | 0.9966 | 0.0234 | 0.9639 | 0.8548 | 0.2446 | 0.4023 |
| Embryonic stem cells (B6J) | 0.9666 | 0.6180 | 0.9839 | 0.0667 | 0.8488 | 0.4689 | 0.4154 | 0.4521 |
| Embryonic stem cells (ZHBTc4) | 0.8161 | 0.7265 | 0.9937 | 0.0344 | 0.6391 | 0.5539 | 0.4243 | 0.4817 |
| Inner cell mass (E3.5) | 0.6237 | 0.7854 | 0.9926 | 0.3857 | 0.9342 | 0.9847 | 0.5546 | 0.9881 |
| Inner cell mass (E3.75) | 0.3065 | 0.9828 | 0.9662 | 0.0548 | 0.9760 | 0.9044 | 0.6890 | 0.9952 |
| Blastocyst (Late) PrE | 0.2110 | 0.7987 | 0.9966 | 0.0583 | 0.9750 | 0.8782 | 0.8915 | 0.9645 |
| Blastocyst (Mid) TE | 0.4021 | 0.8470 | 0.9367 | 0.0994 | 0.9842 | 0.8623 | 0.9072 | 0.9233 |
| Blastocyst (Mid) ICM | 0.7680 | 0.9173 | 0.9958 | 0.0440 | 0.9842 | 0.9406 | 0.9169 | 0.9390 |
| Neonatal tail fibroblast | 0.8285 | 0.8623 | 0.9781 | 0.6488 | 0.8864 | 0.9575 | 0.8989 | 0.7591 |
| Oocytes | 0.9779 | 0.8955 | 0.9934 | 0.0079 | 0.2851 | 0.8307 | 0.1066 | 0.9936 |
| Pronuclei | 0.9935 | 0.9318 | 0.9948 | 0.0129 | 0.3055 | 0.8865 | 0.0720 | 0.9934 |
| Spermatids (round) | 0.9639 | 0.9744 | 0.7187 | 0.2608 | 0.9318 | 0.9526 | 0.4444 | 0.9826 |
| Spermatids | 0.9927 | 0.9748 | 0.6619 | 0.2431 | 0.9842 | 0.9336 | 0.7590 | 0.9628 |
| Testicular cells | 0.9817 | 0.9590 | 0.7141 | 0.3888 | 0.4591 | 0.9518 | 0.6884 | 0.9437 |
| Zygote | 0.9919 | 0.8907 | 0.9886 | 0.0043 | 0.3500 | 0.8082 | 0.1107 | 0.9663 |
| Brain | 0.9683 | 0.0071 | 0.3336 | 0.0575 | 0.0014 | 0.0018 | 0.5326 | 0.0319 |
| Cerebrum | 0.8964 | 0.0034 | 0.0623 | 0.0736 | 0.0003 | 0.0016 | 0.9847 | 0.0901 |
| Suprabasal epidermis (E14.5) | 0.0714 | 0.0869 | 0.1753 | 0.4408 | 0.9622 | 0.7802 | 0.8756 | 0.1501 |
| Epididymis | 0.6082 | 0.1781 | 0.9691 | 0.9698 | 0.0102 | 0.9498 | 0.7631 | 0.3099 |
| Eye | 0.9146 | 0.3878 | 0.9663 | 0.9772 | 0.0050 | 0.5522 | 0.9895 | 0.5755 |
| Frontal cortex | 0.8862 | 0.2293 | 0.2818 | 0.4118 | 0.0181 | 0.4133 | 0.9707 | 0.7676 |
| Hippocampus | 0.7783 | 0.1258 | 0.2789 | 0.7548 | 0.0093 | 0.2090 | 0.9568 | 0.8510 |
| Kidney | 0.4498 | 0.2204 | 0.9871 | 0.9356 | 0.0129 | 0.8326 | 0.9888 | 0.6376 |
| Spermatocytes (leptotene) | 0.8670 | 0.8023 | 0.9575 | 0.9361 | 0.1332 | 0.7314 | 0.3209 | 0.6566 |
| Lung fibroblasts | 0.8443 | 0.2074 | 0.9862 | 0.4066 | 0.0257 | 0.7152 | 0.7368 | 0.1187 |
| Spermatocytes (pachytene) | 0.9727 | 0.9001 | 0.8951 | 0.9513 | 0.1485 | 0.7863 | 0.4837 | 0.9662 |
| Pineal gland | 0.8979 | 0.1194 | 0.9040 | 0.5264 | 0.0014 | 0.3798 | 0.7612 | 0.4829 |
| Spinal cord | 0.8871 | 0.3466 | 0.5941 | 0.5216 | 0.0082 | 0.1353 | 0.9699 | 0.3379 |
| Spermatogonia (type B) | 0.7761 | 0.5542 | 0.9285 | 0.8761 | 0.1511 | 0.5827 | 0.1412 | 0.7923 |
| Vas deferens | 0.6732 | 0.3672 | 0.9563 | 0.9698 | 0.0373 | 0.9447 | 0.9518 | 0.3703 |
| White-matter glia | 0.9375 | 0.1588 | 0.5736 | 0.5948 | 0.0077 | 0.3082 | 0.7124 | 0.1391 |

| **Supplementary Table 10.**  Difference score for each surface marker and TF selected by MIMIC in an extended dataset | | | | | | | | | | | | | | |
| --- | --- | --- | --- | --- | --- | --- | --- | --- | --- | --- | --- | --- | --- | --- |
| Cell type | Adam8 | Csf1r | Aebp1 | Ank2 | Bcl11a | Cebpa | Enpp2 | Gli3 | Irf8 | Meis2 | Nfib | Notch3 | Tcf7 | Zfp57 |
| Monocyte | 0.9914 | 0.7754 | 0.9486 | 0.73 | 0.2546 | 0.2122 | 0.7078 | 0.8879 | 0.4815 | 0.2795 | 0.8619 | 0.9222 | 0.4133 | 0.8603 |
| BMDM | 0.5308 | 0.2018 | 0.4537 | 0.624 | 0.9779 | 0.3692 | 0.5453 | 0.4592 | 0.3536 | 0.617 | 0.6281 | 0.9105 | 0.477 | 0.814 |
| BMDM +CpolyG | 0.2168 | 0.2018 | 0.3019 | 0.5803 | 0.9712 | 0.522 | 0.3178 | 0.3438 | 0.3344 | 0.745 | 0.5645 | 0.8247 | 0.4133 | 0.61 |
| BMDM +IL-1b | 0.1906 | 0.2018 | 0.3089 | 0.5636 | 0.9877 | 0.4837 | 0.3178 | 0.3267 | 0.4327 | 0.617 | 0.6092 | 0.8247 | 0.2815 | 0.6549 |
| BMDM -lipidA | 0.2864 | 0.2018 | 0.989 | 0.5691 | 0.9827 | 0.2352 | 0.899 | 0.8879 | 0.4045 | 0.6536 | 0.945 | 0.948 | 0.7039 | 0.8603 |
| BMDM +lipidA 20 mins | 0.3376 | 0.5037 | 0.9646 | 0.538 | 0.9877 | 0.6092 | 0.899 | 0.8879 | 0.4198 | 0.6004 | 0.7487 | 0.948 | 0.7039 | 0.8603 |
| BMDM +lipidA 30 mins | 0.3245 | 0.2018 | 0.989 | 0.5636 | 0.9628 | 0.2909 | 0.899 | 0.8879 | 0.423 | 0.6738 | 0.945 | 0.9105 | 0.7039 | 0.8603 |
| BMDM +lipidA 360 mins | 0.5439 | 0.589 | 0.989 | 0.5861 | 0.9779 | 0.767 | 0.4983 | 0.8879 | 0.3925 | 0.745 | 0.945 | 0.948 | 0.7039 | 0.8603 |
| BMDM +lipidA 60 mins | 0.3903 | 0.4592 | 0.9801 | 0.5158 | 0.9779 | 0.4333 | 0.899 | 0.8879 | 0.4961 | 0.7193 | 0.8721 | 0.948 | 0.7039 | 0.8603 |
| BMDM +MALP2 | 0.1906 | 0.2018 | 0.3399 | 0.5803 | 0.9851 | 0.6099 | 0.3178 | 0.2826 | 0.3455 | 0.6738 | 0.5645 | 0.7597 | 0.477 | 0.8603 |
| BMDM +IFNg | 0.3245 | 0.3855 | 0.1774 | 0.0881 | 0.9851 | 0.5757 | 0.2327 | 0.2701 | 0.0782 | 0.617 | 0.6092 | 0.7798 | 0.4133 | 0.7084 |
| BMDM +IL-4 | 0.1235 | 0.4592 | 0.3378 | 0.5531 | 0.9779 | 0.2909 | 0.3552 | 0.3841 | 0.4646 | 0.6738 | 0.5859 | 0.8014 | 0.3651 | 0.61 |
| BMDM +IFNa | 0.1906 | 0.2018 | 0.3234 | 0.6526 | 0.9827 | 0.4846 | 0.2986 | 0.3267 | 0.3925 | 0.7193 | 0.5859 | 0.8503 | 0.4133 | 0.7743 |
| BMDM +TGFb | 0.2168 | 0.3855 | 0.299 | 0.5861 | 0.9802 | 0.2092 | 0.4142 | 0.335 | 0.261 | 0.6956 | 0.575 | 0.8503 | 0.3273 | 0.6549 |
| DC CD11+ | 0.3837 | 0.5549 | 0.9776 | 0.8957 | 0.1288 | 0.3325 | 0.6591 | 0.6954 | 0.3731 | 0.843 | 0.9622 | 0.9452 | 0.3797 | 0.392 |
| Dendritic cells (BMDC) | 0.2397 | 0.2397 | 0.8547 | 0.7726 | 0.9447 | 0.1715 | 0.5328 | 0.6241 | 0.5845 | 0.7733 | 0.872 | 0.9463 | 0.9537 | 0.8413 |
| Eosinophils -IL-10 -LPS | 0.0163 | 0.1769 | 0.9835 | 0.9797 | 0.939 | 0.6385 | 0.9667 | 0.9821 | 0.9603 | 0.9946 | 0.9926 | 0.9167 | 0.9092 | 0.8284 |
| Eosinophils -IL-10 +LPS | 0.0184 | 0.1375 | 0.9828 | 0.9792 | 0.9362 | 0.6237 | 0.908 | 0.993 | 0.9655 | 0.9945 | 0.9926 | 0.9046 | 0.9201 | 0.8331 |
| Granulocytes | 0.0107 | 0.1382 | 0.9844 | 0.9858 | 0.7771 | 0.2141 | 0.3024 | 0.9887 | 0.8642 | 0.9432 | 0.9936 | 0.9957 | 0.9843 | 0.9923 |
| Macrophages -IL10 -LPS | 0.0067 | 0.0224 | 0.2291 | 0.8544 | 0.9237 | 0.0665 | 0.3224 | 0.7749 | 0.24 | 0.8973 | 0.8434 | 0.9949 | 0.9416 | 0.902 |
| Macrophages -IL10 +LPS | 0.028 | 0.0419 | 0.131 | 0.8445 | 0.9133 | 0.3841 | 0.2001 | 0.6617 | 0.2286 | 0.8183 | 0.7382 | 0.9567 | 0.8833 | 0.883 |
| Macrophages +IL10 -LPS | 0.0093 | 0.0244 | 0.2322 | 0.8359 | 0.9286 | 0.0795 | 0.3077 | 0.7318 | 0.2331 | 0.8676 | 0.8506 | 0.9842 | 0.9207 | 0.9284 |
| Macrophages +IL10 +LPS | 0.0287 | 0.047 | 0.1629 | 0.7822 | 0.9181 | 0.3857 | 0.2325 | 0.6898 | 0.2892 | 0.8595 | 0.7724 | 0.9821 | 0.9057 | 0.9007 |
| Medullary thymic epithelial | 0.0178 | 0.7918 | 0.9623 | 0.5835 | 0.8407 | 0.2202 | 0.1817 | 0.4584 | 0.1772 | 0.1431 | 0.6623 | 0.7122 | 0.2876 | 0.969 |
| Megakaryoblast | 0.2724 | 0.3476 | 0.9838 | 0.7333 | 0.5973 | 0.3403 | 0.9842 | 0.6131 | 0.6521 | 0.7566 | 0.8819 | 0.3689 | 0.9876 | 0.6482 |
| Microglia | 0.4379 | 0.0056 | 0.9047 | 0.212 | 0.861 | 0.0643 | 0.2456 | 0.9849 | 0.258 | 0.8064 | 0.9609 | 0.9946 | 0.9781 | 0.7628 |
| Mast cell -IL10 -LPS | 0.0446 | 0.6095 | 0.9313 | 0.9856 | 0.8267 | 0.5098 | 0.9842 | 0.9787 | 0.9546 | 0.7583 | 0.9963 | 0.9264 | 0.9383 | 0.8371 |
| Mast cell -IL10 +LPS | 0.1429 | 0.6289 | 0.9778 | 0.992 | 0.691 | 0.5452 | 0.9643 | 0.9786 | 0.9569 | 0.6009 | 0.9962 | 0.8787 | 0.9276 | 0.7837 |
| Mast cell +IL10 -LPS | 0.0353 | 0.6243 | 0.906 | 0.9635 | 0.8523 | 0.4892 | 0.9689 | 0.9784 | 0.9527 | 0.7297 | 0.9961 | 0.911 | 0.9239 | 0.8589 |
| Mast cell +IL10 +LPS | 0.1128 | 0.6312 | 0.949 | 0.9919 | 0.7463 | 0.4886 | 0.9545 | 0.9782 | 0.9679 | 0.64 | 0.9927 | 0.8361 | 0.9324 | 0.78 |
| Myelocytes | 0.0415 | 0.1863 | 0.9877 | 0.9632 | 0.6951 | 0.3867 | 0.5722 | 0.978 | 0.5825 | 0.9623 | 0.9959 | 0.9938 | 0.9818 | 0.9827 |
| Myeloid cells | 0.0128 | 0.0337 | 0.0927 | 0.5886 | 0.8916 | 0.148 | 0.157 | 0.4042 | 0.2054 | 0.642 | 0.8692 | 0.8927 | 0.9339 | 0.9803 |
| Neutrophil -IL10 -LPS | 0.0398 | 0.1472 | 0.9877 | 0.9742 | 0.7012 | 0.2096 | 0.9058 | 0.9722 | 0.5072 | 0.9923 | 0.9963 | 0.9834 | 0.7708 | 0.9462 |
| Neutrophil -IL10 +LPS | 0.0434 | 0.2167 | 0.9877 | 0.9664 | 0.5216 | 0.1929 | 0.9317 | 0.9501 | 0.4364 | 0.9922 | 0.9962 | 0.9703 | 0.7618 | 0.9306 |
| Neutrophil +IL10 -LPS | 0.0345 | 0.1872 | 0.9877 | 0.9664 | 0.6359 | 0.2421 | 0.9184 | 0.9795 | 0.4876 | 0.992 | 0.9961 | 0.9834 | 0.7386 | 0.9432 |
| Neutrophil +IL10 +LPS | 0.0294 | 0.2403 | 0.9566 | 0.9856 | 0.5282 | 0.2566 | 0.935 | 0.9484 | 0.5059 | 0.9919 | 0.996 | 0.9811 | 0.7535 | 0.9253 |
| Orthochromatic erythroblast | 0.5937 | 0.8143 | 0.8923 | 0.8524 | 0.7465 | 0.7486 | 0.9301 | 0.9546 | 0.7938 | 0.9621 | 0.9368 | 0.9936 | 0.9848 | 0.9828 |
| Placenta | 0.3216 | 0.0511 | 0.1289 | 0.558 | 0.9907 | 0.3838 | 0.368 | 0.546 | 0.6226 | 0.554 | 0.6727 | 0.6568 | 0.8215 | 0.8569 |
| splenic DC -IL10 -LPS | 0.0116 | 0.1138 | 0.9826 | 0.9269 | 0.6674 | 0.2278 | 0.8172 | 0.6576 | 0.1319 | 0.9861 | 0.9908 | 0.924 | 0.0231 | 0.9104 |
| splenic DC +IL10 -LPS | 0.0121 | 0.1088 | 0.9826 | 0.9369 | 0.64 | 0.2669 | 0.8194 | 0.739 | 0.1507 | 0.992 | 0.9893 | 0.9198 | 0.0266 | 0.9072 |
| Sperm | 0.0424 | 0.1226 | 0.1332 | 0.786 | 0.971 | 0.1745 | 0.4537 | 0.8861 | 0.3329 | 0.8201 | 0.5147 | 0.7075 | 0.886 | 0.9726 |
| Spermatogonia | 0.2783 | 0.0989 | 0.5676 | 0.1769 | 0.6484 | 0.308 | 0.8472 | 0.8953 | 0.797 | 0.9905 | 0.9763 | 0.9929 | 0.6934 | 0.8103 |
| Polyploid trophoblast giant cells (E9.5) | 0.1698 | 0.057 | 0.6486 | 0.4949 | 0.9898 | 0.9457 | 0.972 | 0.9077 | 0.59 | 0.6514 | 0.926 | 0.9867 | 0.2927 | 0.7157 |
| Trophoblast stem cells | 0.9827 | 0.4327 | 0.369 | 0.8327 | 0.9855 | 0.6865 | 0.9607 | 0.4439 | 0.9819 | 0.8432 | 0.9828 | 0.7461 | 0.6752 | 0.3085 |
| CD4+ iTreg cells | 0.9799 | 0.9824 | 0.9686 | 0.8498 | 0.9676 | 0.9972 | 0.8965 | 0.6172 | 0.3365 | 0.9312 | 0.7734 | 0.9687 | 0.0005 | 0.7548 |
| CD4+ T cells -IL-21 | 0.8974 | 0.9999 | 0.8997 | 0.9739 | 0.9671 | 0.9924 | 0.5279 | 0.8909 | 0.6226 | 0.7753 | 0.9486 | 0.795 | 0.0004 | 0.8651 |
| Naive CD4+ T cells, anti-CD3/28 treated | 0.9869 | 0.9997 | 0.8531 | 0.9673 | 0.9921 | 0.997 | 0.8965 | 0.8879 | 0.7371 | 0.9297 | 0.945 | 0.8014 | 0.001 | 0.7743 |
| CD4+ T cells +IL-21 | 0.9001 | 0.9999 | 0.9378 | 0.9558 | 0.9687 | 0.9848 | 0.4592 | 0.8847 | 0.3795 | 0.8029 | 0.9409 | 0.6612 | 0.0014 | 0.7666 |
| CD4+ Th17 cells | 0.952 | 0.9902 | 0.9718 | 0.7615 | 0.9956 | 0.9966 | 0.2679 | 0.3753 | 0.5268 | 0.7406 | 0.8909 | 0.4448 | 0.0029 | 0.8493 |
| CD4+ Th1 cells | 0.7495 | 0.9905 | 0.9841 | 0.6837 | 0.9952 | 0.9966 | 0.3931 | 0.8813 | 0.1932 | 0.6853 | 0.8248 | 0.235 | 0.0118 | 0.1143 |
| CD4+ Th2 cells | 0.7397 | 0.9943 | 0.9575 | 0.7701 | 0.9887 | 0.9965 | 0.3135 | 0.7948 | 0.7903 | 0.4296 | 0.9144 | 0.2466 | 0.0302 | 0.4706 |
| CD4+ Th9 cells | 0.998 | 0.9998 | 0.8436 | 0.9254 | 0.9944 | 0.9965 | 0.6894 | 0.7461 | 0.1996 | 0.9086 | 0.8958 | 0.1525 | 0.0174 | 0.6448 |
| CD4+ Treg cells | 0.9918 | 0.9982 | 0.8647 | 0.7583 | 0.9939 | 0.9935 | 0.7743 | 0.8056 | 0.2339 | 0.7791 | 0.8651 | 0.4529 | 0.02 | 0.4853 |
| CD4+ T cells (anti-CD3/CD28 treated) | 0.9413 | 0.9895 | 0.7962 | 0.6527 | 0.7574 | 0.9268 | 0.6894 | 0.8805 | 0.2476 | 0.8255 | 0.8651 | 0.8418 | 0.0317 | 0.8352 |
| CD4+ T cells | 0.9514 | 0.9906 | 0.988 | 0.8696 | 0.784 | 0.9262 | 0.8965 | 0.8796 | 0.9231 | 0.8895 | 0.8651 | 0.5645 | 0.0094 | 0.8179 |
| CD8+ T cells | 0.9205 | 0.8621 | 0.9391 | 0.5894 | 0.5647 | 0.9189 | 0.5965 | 0.8786 | 0.3807 | 0.8839 | 0.8651 | 0.3309 | 0.0816 | 0.7958 |
| CD8+ T cells +IL-2 | 0.8682 | 0.9913 | 0.6872 | 0.9214 | 0.5094 | 0.988 | 0.8965 | 0.8777 | 0.2935 | 0.8777 | 0.8651 | 0.4151 | 0.3317 | 0.7666 |
| Double negative 1 (DN1) thymic cells | 0.9347 | 0.8778 | 0.6574 | 0.9051 | 0.1753 | 0.7843 | 0.8061 | 0.5856 | 0.8658 | 0.6032 | 0.9596 | 0.0142 | 0.0707 | 0.6831 |
| Double negative 2a (DN2a) thymic cells | 0.9064 | 0.9921 | 0.8493 | 0.934 | 0.4092 | 0.9766 | 0.8647 | 0.6884 | 0.921 | 0.6635 | 0.9654 | 0.0229 | 0.0528 | 0.8417 |
| Double negative 2b (DN2b) thymic cells | 0.9574 | 0.9951 | 0.5757 | 0.9246 | 0.7146 | 0.9098 | 0.786 | 0.6525 | 0.9755 | 0.4576 | 0.924 | 0.003 | 0.0343 | 0.7205 |
| Double negative 3 (DN3) thymic cells | 0.965 | 0.9991 | 0.9118 | 0.939 | 0.765 | 0.9938 | 0.8817 | 0.899 | 0.9311 | 0.8783 | 0.9787 | 0.0022 | 0.0527 | 0.9152 |
| Double positive thymic cells | 0.9586 | 0.9987 | 0.9753 | 0.9096 | 0.9888 | 0.9792 | 0.9076 | 0.899 | 0.9428 | 0.7886 | 0.9916 | 0.0635 | 0.018 | 0.9048 |
| Embryonic stem cells (E14) | 0.8694 | 0.9605 | 0.2238 | 0.579 | 0.9356 | 0.9429 | 0.5735 | 0.4301 | 0.9885 | 0.9242 | 0.6503 | 0.1546 | 0.2313 | 0.0568 |
| Innate lymphoid cell (Type 2) | 0.0786 | 0.9776 | 0.924 | 0.9687 | 0.9888 | 0.9919 | 0.9758 | 0.9729 | 0.9106 | 0.9658 | 0.9677 | 0.9364 | 0.0878 | 0.8122 |
| Spermatocytes (leptotene) | 0.9587 | 0.7586 | 0.4221 | 0.7955 | 0.9575 | 0.867 | 0.1332 | 0.473 | 0.9789 | 0.9857 | 0.8023 | 0.6566 | 0.3209 | 0.9361 |
| Naive CD4+ CD25- T cells | 0.7892 | 0.6772 | 0.9877 | 0.9782 | 0.8245 | 0.8092 | 0.8026 | 0.9782 | 0.7642 | 0.9644 | 0.9816 | 0.9791 | 0.0033 | 0.9904 |
| Primordial germ cells | 0.9919 | 0.9927 | 0.966 | 0.9581 | 0.9038 | 0.9929 | 0.7884 | 0.3329 | 0.6751 | 0.7011 | 0.9515 | 0.9715 | 0.2268 | 0.8197 |
| Spermatogonia (primitive type A) | 0.9831 | 0.9634 | 0.2532 | 0.8173 | 0.9005 | 0.6685 | 0.1239 | 0.1235 | 0.8181 | 0.9656 | 0.5277 | 0.9111 | 0.0578 | 0.9042 |
| splenic DC -IL10 +LPS | 0.0197 | 0.546 | 0.9732 | 0.9329 | 0.7054 | 0.6497 | 0.7326 | 0.6467 | 0.1038 | 0.9858 | 0.9832 | 0.9155 | 0.0225 | 0.8906 |
| splenic DC +IL10 +LPS | 0.0206 | 0.3065 | 0.9689 | 0.9453 | 0.6311 | 0.5959 | 0.6734 | 0.7374 | 0.1478 | 0.9885 | 0.9854 | 0.9277 | 0.0292 | 0.9173 |
| Spleen | 0.4444 | 0.0773 | 0.4306 | 0.8379 | 0.1254 | 0.6606 | 0.0801 | 0.8715 | 0.1002 | 0.8874 | 0.6621 | 0.5107 | 0.0592 | 0.9623 |
| Thymus | 0.6431 | 0.4583 | 0.6885 | 0.9242 | 0.7648 | 0.6144 | 0.3436 | 0.6017 | 0.4039 | 0.7531 | 0.775 | 0.2832 | 0.0023 | 0.9785 |
| Spermatogonia (type A) | 0.9755 | 0.971 | 0.3909 | 0.8396 | 0.9063 | 0.7845 | 0.1311 | 0.1746 | 0.9224 | 0.956 | 0.5413 | 0.8922 | 0.111 | 0.9145 |
| Spermatogonia (type B) | 0.9753 | 0.7989 | 0.2386 | 0.8642 | 0.9285 | 0.7761 | 0.1511 | 0.2087 | 0.9432 | 0.9546 | 0.5542 | 0.7923 | 0.1412 | 0.8761 |
| Brain | 0.9973 | 0.956 | 0.675 | 0.0009 | 0.3336 | 0.9683 | 0.0014 | 0.1619 | 0.9927 | 0.0269 | 0.0071 | 0.0319 | 0.5326 | 0.0575 |
| Cerebellum | 0.9987 | 0.9799 | 0.5268 | 0.0009 | 0.2113 | 0.9748 | 0.0027 | 0.1612 | 0.9981 | 0.3078 | 0.0019 | 0.088 | 0.9958 | 0.2257 |
| Cerebrum | 0.9955 | 0.8549 | 0.4423 | 0.0011 | 0.0623 | 0.8964 | 0.0003 | 0.0723 | 0.9856 | 0.005 | 0.0034 | 0.0901 | 0.9847 | 0.0736 |
| Cortex | 0.9971 | 0.8606 | 0.5986 | 0.0006 | 0.0717 | 0.9636 | 0.0063 | 0.0956 | 0.9937 | 0.0081 | 0.0114 | 0.3057 | 0.9947 | 0.0989 |
| Dentate gyrus | 0.9739 | 0.8212 | 0.7402 | 0.0029 | 0.3653 | 0.6805 | 0.0394 | 0.1921 | 0.9839 | 0.5127 | 0.0207 | 0.3516 | 0.9894 | 0.113 |
| Dorsal rootganglia | 0.6793 | 0.9964 | 0.0324 | 0.0019 | 0.8939 | 0.7871 | 0.4823 | 0.3081 | 0.998 | 0.2676 | 0.217 | 0.1169 | 0.9931 | 0.4526 |
| Spermatids (elogated) | 0.9943 | 0.8107 | 0.8383 | 0.1409 | 0.7978 | 0.8192 | 0.8032 | 0.8383 | 0.9965 | 0.9927 | 0.9727 | 0.9968 | 0.7832 | 0.2411 |
| Eye | 0.9778 | 0.5248 | 0.0995 | 0.0307 | 0.9663 | 0.9146 | 0.005 | 0.8648 | 0.9954 | 0.2148 | 0.3878 | 0.5755 | 0.9895 | 0.9772 |
| Frontal cortex | 0.9658 | 0.3323 | 0.6608 | 0.0254 | 0.2818 | 0.8862 | 0.0181 | 0.8073 | 0.9894 | 0.159 | 0.2293 | 0.7676 | 0.9707 | 0.4118 |
| Hippocampus | 0.9481 | 0.1903 | 0.3019 | 0.0143 | 0.2789 | 0.7783 | 0.0093 | 0.7004 | 0.9827 | 0.3171 | 0.1258 | 0.851 | 0.9568 | 0.7548 |
| Motor neurons | 0.8977 | 0.7994 | 0.9586 | 0.0061 | 0.8394 | 0.9576 | 0.1414 | 0.7846 | 0.9929 | 0.1425 | 0.1988 | 0.6889 | 0.942 | 0.6983 |
| Spermatocytes (pachytene) | 0.9559 | 0.9881 | 0.8576 | 0.7677 | 0.8951 | 0.9727 | 0.1485 | 0.6378 | 0.9925 | 0.9938 | 0.9001 | 0.9662 | 0.4837 | 0.9513 |
| Pineal gland | 0.9372 | 0.3513 | 0.0829 | 0.0715 | 0.904 | 0.8979 | 0.0014 | 0.7043 | 0.5235 | 0.9904 | 0.1194 | 0.4829 | 0.7612 | 0.5264 |
| Retina | 0.91 | 0.9069 | 0.8232 | 0.0151 | 0.8419 | 0.9903 | 0.1792 | 0.8535 | 0.9867 | 0.1387 | 0.5681 | 0.898 | 0.9811 | 0.8902 |
| Spermatids (round) | 0.9902 | 0.7725 | 0.7668 | 0.1047 | 0.7187 | 0.9639 | 0.9318 | 0.6878 | 0.9925 | 0.9924 | 0.9744 | 0.9826 | 0.4444 | 0.2608 |
| Spermatids | 0.9906 | 0.9398 | 0.9689 | 0.0964 | 0.6619 | 0.9927 | 0.9842 | 0.8334 | 0.9965 | 0.9909 | 0.9748 | 0.9628 | 0.759 | 0.2431 |
| Spinal cord | 0.9779 | 0.3728 | 0.1714 | 0.0116 | 0.5941 | 0.8871 | 0.0082 | 0.5858 | 0.9823 | 0.1879 | 0.3466 | 0.3379 | 0.9699 | 0.5216 |
| Striatum | 0.9467 | 0.2572 | 0.8693 | 0.0182 | 0.3524 | 0.8363 | 0.0407 | 0.2767 | 0.9169 | 0.032 | 0.6559 | 0.5587 | 0.6275 | 0.9285 |
| Substantia nigra | 0.8378 | 0.614 | 0.9718 | 0.0248 | 0.7013 | 0.9935 | 0.0355 | 0.915 | 0.9924 | 0.9356 | 0.8402 | 0.8046 | 0.9575 | 0.3482 |
| Testicular cells | 0.891 | 0.9131 | 0.6271 | 0.1446 | 0.7141 | 0.9817 | 0.4591 | 0.8843 | 0.9789 | 0.9873 | 0.959 | 0.9437 | 0.6884 | 0.3888 |
| Ventral tagmental | 0.475 | 0.6773 | 0.9205 | 0.0401 | 0.3916 | 0.9919 | 0.0689 | 0.3268 | 0.9953 | 0.9855 | 0.7558 | 0.7902 | 0.8397 | 0.1922 |
| White-matter glia | 0.9778 | 0.1431 | 0.4057 | 0.0345 | 0.5736 | 0.9375 | 0.0077 | 0.1382 | 0.7558 | 0.2068 | 0.1588 | 0.1391 | 0.7124 | 0.5948 |
| Caecum (E13.5) | 0.998 | 0.9939 | 0.3931 | 0.024 | 0.432 | 0.9386 | 0.108 | 0.0061 | 0.991 | 0.0023 | 0.0054 | 0.0012 | 0.026 | 0.0078 |
| Cardiac precursors | 0.9987 | 0.9999 | 0.2565 | 0.0932 | 0.9933 | 0.9972 | 0.899 | 0.0099 | 0.998 | 0.0066 | 0.1862 | 0.001 | 0.0098 | 0.0049 |
| Central neural epithelium (E10.5) | 0.8971 | 0.9851 | 0.8105 | 0.0335 | 0.1808 | 0.9936 | 0.3263 | 0.0023 | 0.9971 | 0.0237 | 0.2693 | 0.0144 | 0.913 | 0.0114 |
| Dorsal control neural epithelium (E10.5) | 0.8988 | 0.999 | 0.6369 | 0.0267 | 0.1732 | 0.9935 | 0.4888 | 0.0064 | 0.9971 | 0.0375 | 0.2345 | 0.0158 | 0.8182 | 0.0077 |
| Forelimb (E10.5) | 0.9731 | 0.9761 | 0.5373 | 0.2873 | 0.4865 | 0.948 | 0.1382 | 0.004 | 0.9946 | 0.0165 | 0.6255 | 0.0181 | 0.703 | 0.0343 |
| Hindlimb (E10.5) | 0.9713 | 0.9762 | 0.5103 | 0.2653 | 0.6453 | 0.9527 | 0.2406 | 0.0034 | 0.9934 | 0.0283 | 0.756 | 0.0341 | 0.556 | 0.0549 |
| Lateral prominence neural epithelium (E10.5) | 0.9512 | 0.944 | 0.7511 | 0.0215 | 0.1883 | 0.9931 | 0.5073 | 0.0026 | 0.9984 | 0.0155 | 0.5365 | 0.0686 | 0.9584 | 0.0342 |
| Mandibular arch (E10.5) | 0.9739 | 0.8952 | 0.1711 | 0.1364 | 0.1363 | 0.9929 | 0.0486 | 0.0076 | 0.9984 | 0.0308 | 0.2444 | 0.1542 | 0.8335 | 0.018 |
| Maxillary arch (E10.5) | 0.9969 | 0.9069 | 0.2036 | 0.1587 | 0.126 | 0.9927 | 0.066 | 0.0046 | 0.9984 | 0.0287 | 0.2677 | 0.1257 | 0.7132 | 0.0246 |
| Medial nasal prominence (E10.5) | 0.9484 | 0.5977 | 0.3118 | 0.1731 | 0.3413 | 0.9924 | 0.0138 | 0.0158 | 0.9828 | 0.0775 | 0.3824 | 0.1179 | 0.9939 | 0.0405 |
| Dermis (E16.5) | 0.934 | 0.7178 | 0.0767 | 0.2724 | 0.3013 | 0.1416 | 0.043 | 0.0903 | 0.9932 | 0.444 | 0.1241 | 0.063 | 0.6233 | 0.3651 |
| Caudal brain neural epithelium (E8.5) | 0.83 | 0.9804 | 0.6753 | 0.2186 | 0.9907 | 0.9936 | 0.1317 | 0.0115 | 0.9983 | 0.1887 | 0.9827 | 0.2041 | 0.4092 | 0.1995 |
| Non-floor plate neural epithelium (E8.5) | 0.8793 | 0.9967 | 0.8879 | 0.2358 | 0.9972 | 0.9935 | 0.9395 | 0.0077 | 0.9983 | 0.149 | 0.9827 | 0.1885 | 0.6074 | 0.2928 |
| Cranial mesenchyme (E9.5) | 0.9715 | 0.8801 | 0.4741 | 0.3995 | 0.9789 | 0.9879 | 0.2839 | 0.0267 | 0.9906 | 0.5915 | 0.7687 | 0.4116 | 0.5812 | 0.0911 |
| Epidermal ectoderm (E9.5) | 0.9196 | 0.9513 | 0.7124 | 0.3662 | 0.6592 | 0.9876 | 0.3924 | 0.0069 | 0.9969 | 0.0652 | 0.492 | 0.1351 | 0.3406 | 0.1985 |
| Mandibular arch (E9.5) | 0.9186 | 0.9964 | 0.3806 | 0.3462 | 0.89 | 0.9931 | 0.5092 | 0.0228 | 0.9983 | 0.0826 | 0.6948 | 0.2398 | 0.4115 | 0.075 |
| Maxillary arch (E9.5) | 0.8331 | 0.9139 | 0.4341 | 0.3454 | 0.5821 | 0.9929 | 0.2283 | 0.0258 | 0.9942 | 0.0437 | 0.6275 | 0.2309 | 0.7008 | 0.0966 |
| EpiSC | 0.8868 | 0.9891 | 0.3297 | 0.3486 | 0.807 | 0.9552 | 0.043 | 0.2215 | 0.9904 | 0.5585 | 0.7978 | 0.1326 | 0.2411 | 0.2848 |
| Embryonic stem cells (V6.5) | 0.8085 | 0.9944 | 0.1248 | 0.3348 | 0.9289 | 0.9351 | 0.0792 | 0.1847 | 0.9912 | 0.7796 | 0.6656 | 0.0592 | 0.2214 | 0.1446 |
| Ileum | 0.9396 | 0.814 | 0.2291 | 0.2754 | 0.4854 | 0.6663 | 0.5581 | 0.0553 | 0.9153 | 0.0944 | 0.1538 | 0.1487 | 0.7259 | 0.3348 |
| Mesodermal cells | 0.9182 | 0.9952 | 0.6499 | 0.5905 | 0.9162 | 0.9771 | 0.7588 | 0.0542 | 0.9962 | 0.3766 | 0.9561 | 0.0821 | 0.2273 | 0.2932 |
| Neocortex ventricular zone | 0.8121 | 0.9143 | 0.941 | 0.0943 | 0.1764 | 0.9765 | 0.8122 | 0.0136 | 0.9841 | 0.02 | 0.0398 | 0.0997 | 0.8723 | 0.267 |
| Neuron cell NCAM+ | 0.956 | 0.9464 | 0.4108 | 0.2199 | 0.7139 | 0.9862 | 0.1736 | 0.3727 | 0.9964 | 0.1333 | 0.51 | 0.2462 | 0.8441 | 0.1426 |
| Nucleus accumbens | 0.9791 | 0.3418 | 0.7881 | 0.019 | 0.2353 | 0.8866 | 0.0397 | 0.3691 | 0.9673 | 0.0269 | 0.5606 | 0.4252 | 0.8602 | 0.8997 |
| Olfactory bulb | 0.966 | 0.4547 | 0.2529 | 0.012 | 0.1159 | 0.921 | 0.0581 | 0.6388 | 0.9785 | 0.005 | 0.453 | 0.5784 | 0.8731 | 0.6384 |
| Subventricular zone | 0.9438 | 0.3297 | 0.2377 | 0.0282 | 0.2995 | 0.8225 | 0.0534 | 0.399 | 0.9191 | 0.0251 | 0.3563 | 0.6287 | 0.7645 | 0.511 |
| Upper molar | 0.9141 | 0.8358 | 0.172 | 0.155 | 0.1115 | 0.8862 | 0.1317 | 0.0108 | 0.9835 | 0.5467 | 0.0483 | 0.0914 | 0.1616 | 0.2845 |
| Blastocyst (Early) TE | 0.9943 | 0.9022 | 0.9018 | 0.8438 | 0.9878 | 0.464 | 0.9673 | 0.9879 | 0.9982 | 0.9632 | 0.8433 | 0.9972 | 0.7987 | 0.0692 |
| Blastocyst (Early) ICM | 0.9488 | 0.7394 | 0.6324 | 0.5414 | 0.9967 | 0.456 | 0.9667 | 0.8792 | 0.9982 | 0.8485 | 0.9117 | 0.9854 | 0.763 | 0.0256 |
| 2C Embyro (early) | 0.9943 | 0.8859 | 0.9846 | 0.9529 | 0.9895 | 0.9919 | 0.1569 | 0.0238 | 0.7822 | 0.0333 | 0.8119 | 0.9965 | 0.1563 | 0.0021 |
| 2C Embyro (late) | 0.8168 | 0.8271 | 0.9016 | 0.9533 | 0.9945 | 0.6613 | 0.51 | 0.0366 | 0.996 | 0.2557 | 0.8244 | 0.9522 | 0.3732 | 0.0224 |
| 2C Embyro (middle) | 0.9929 | 0.8552 | 0.9835 | 0.8213 | 0.9174 | 0.6397 | 0.2449 | 0.0427 | 0.8634 | 0.2102 | 0.8688 | 0.9793 | 0.2329 | 0.0396 |
| 2C Embryo | 0.9557 | 0.9313 | 0.9231 | 0.9496 | 0.9839 | 0.7087 | 0.8779 | 0.1991 | 0.9583 | 0.2739 | 0.8364 | 0.8855 | 0.3509 | 0.0598 |
| Epiblast | 0.6556 | 0.8592 | 0.9821 | 0.9927 | 0.8816 | 0.9919 | 0.9654 | 0.9929 | 0.998 | 0.4354 | 0.5638 | 0.9314 | 0.4204 | 0.4497 |
| Naive ESC (Hex-) | 0.9827 | 0.9221 | 0.9039 | 0.7825 | 0.9966 | 0.9822 | 0.9654 | 0.6919 | 0.9913 | 0.9069 | 0.449 | 0.4439 | 0.2223 | 0.0269 |
| Naive ESC (Hex+) | 0.9689 | 0.9473 | 0.9548 | 0.784 | 0.9966 | 0.9849 | 0.9639 | 0.7457 | 0.9829 | 0.9013 | 0.4763 | 0.4023 | 0.2446 | 0.0234 |
| Embryonic stem cells (B6J) | 0.9344 | 0.9946 | 0.5449 | 0.6979 | 0.9839 | 0.9666 | 0.8488 | 0.6051 | 0.9944 | 0.9272 | 0.618 | 0.4521 | 0.4154 | 0.0667 |
| Embryonic stem cells (ZHBTc4) | 0.8405 | 0.904 | 0.9133 | 0.861 | 0.9937 | 0.8161 | 0.6391 | 0.7958 | 0.9655 | 0.9492 | 0.7265 | 0.4817 | 0.4243 | 0.0344 |
| Inner cell mass (E3.75) | 0.2627 | 0.961 | 0.4064 | 0.7201 | 0.9662 | 0.3065 | 0.976 | 0.916 | 0.9974 | 0.8022 | 0.9828 | 0.9952 | 0.689 | 0.0548 |
| Blastocyst (Late) PrE | 0.6565 | 0.6449 | 0.6884 | 0.7484 | 0.9966 | 0.211 | 0.975 | 0.9885 | 0.9972 | 0.761 | 0.7987 | 0.9645 | 0.8915 | 0.0583 |
| Blastocyst (Mid) TE | 0.984 | 0.7181 | 0.9228 | 0.7934 | 0.9367 | 0.4021 | 0.9842 | 0.9834 | 0.9974 | 0.9039 | 0.847 | 0.9233 | 0.9072 | 0.0994 |
| Blastocyst (Mid) ICM | 0.7235 | 0.8537 | 0.5083 | 0.6242 | 0.9958 | 0.768 | 0.9842 | 0.8725 | 0.9932 | 0.8412 | 0.9173 | 0.939 | 0.9169 | 0.044 |
| Oocytes | 0.2708 | 0.8499 | 0.9877 | 0.9121 | 0.9934 | 0.9779 | 0.2851 | 0.0785 | 0.5146 | 0.0414 | 0.8955 | 0.9936 | 0.1066 | 0.0079 |
| Pronuclei | 0.4958 | 0.8378 | 0.9877 | 0.992 | 0.9948 | 0.9935 | 0.3055 | 0.0863 | 0.5537 | 0.0422 | 0.9318 | 0.9934 | 0.072 | 0.0129 |
| Zygote | 0.9843 | 0.6582 | 0.9884 | 0.8591 | 0.9886 | 0.9919 | 0.35 | 0.0234 | 0.551 | 0.027 | 0.8907 | 0.9663 | 0.1107 | 0.0043 |
| Cardiomyocytes | 0.9994 | 0.9995 | 0.0445 | 0.0477 | 0.9022 | 0.996 | 0.8305 | 0.0265 | 0.99 | 0.0186 | 0.0536 | 0.0079 | 0.025 | 0.0099 |
| Dermal fibroblast | 0.7009 | 0.8585 | 0.0142 | 0.1282 | 0.9473 | 0.4276 | 0.5536 | 0.0196 | 0.9438 | 0.0547 | 0.1034 | 0.0617 | 0.9709 | 0.0949 |
| Intestinal subepithelial myofibroblasts (adult) | 0.3188 | 0.6463 | 0.0387 | 0.5434 | 0.9795 | 0.8131 | 0.9095 | 0.2248 | 0.9814 | 0.2839 | 0.293 | 0.9249 | 0.9308 | 0.355 |
| Intestinal subepithelial myofibroblasts (neonatal) | 0.9487 | 0.9804 | 0.0134 | 0.2607 | 0.9966 | 0.9728 | 0.8073 | 0.3077 | 0.9972 | 0.295 | 0.2642 | 0.4359 | 0.8803 | 0.3354 |
| Lung fibroblasts | 0.7766 | 0.9528 | 0.0076 | 0.3909 | 0.9862 | 0.8443 | 0.0257 | 0.2302 | 0.9967 | 0.5562 | 0.2074 | 0.1187 | 0.7368 | 0.4066 |
| Mature sperm | 0.9838 | 0.5384 | 0.1239 | 0.5472 | 0.5195 | 0.8391 | 0.8687 | 0.9816 | 0.9949 | 0.9699 | 0.7173 | 0.8539 | 0.993 | 0.6307 |
| MEF | 0.6251 | 0.9222 | 0.0187 | 0.3157 | 0.8614 | 0.8816 | 0.695 | 0.0599 | 0.9973 | 0.3279 | 0.2213 | 0.27 | 0.9461 | 0.3084 |
| Myoblast | 0.153 | 0.2109 | 0.0218 | 0.3637 | 0.9748 | 0.6901 | 0.9199 | 0.0969 | 0.9315 | 0.4697 | 0.4271 | 0.5953 | 0.8752 | 0.4374 |
| Neonatal tail fibroblast | 0.3019 | 0.9396 | 0.0222 | 0.2845 | 0.9781 | 0.8285 | 0.8864 | 0.1094 | 0.9969 | 0.6255 | 0.8623 | 0.7591 | 0.8989 | 0.6488 |
| Oviduct | 0.0269 | 0.7752 | 0.0136 | 0.8996 | 0.9368 | 0.4335 | 0.0532 | 0.8449 | 0.9074 | 0.1424 | 0.1533 | 0.3474 | 0.9044 | 0.9026 |
| pre-Adipocytes | 0.795 | 0.861 | 0.015 | 0.2576 | 0.979 | 0.0774 | 0.5033 | 0.1045 | 0.9748 | 0.4247 | 0.2411 | 0.1918 | 0.9772 | 0.6581 |
| pre-Adipocytes (brown) | 0.8685 | 0.8557 | 0.0171 | 0.4027 | 0.9786 | 0.1133 | 0.3142 | 0.1794 | 0.9782 | 0.5673 | 0.1756 | 0.3516 | 0.9907 | 0.4684 |
| Skeletal muscle | 0.9745 | 0.5971 | 0.0511 | 0.1923 | 0.9908 | 0.3942 | 0.1862 | 0.7103 | 0.8993 | 0.9097 | 0.1253 | 0.3221 | 0.9674 | 0.9488 |
| Striatal neurons | 0.9636 | 0.9842 | 0.0428 | 0.6438 | 0.9946 | 0.873 | 0.856 | 0.0535 | 0.9968 | 0.1469 | 0.1496 | 0.5014 | 0.1821 | 0.7415 |
| tail fibroblast | 0.4932 | 0.7051 | 0.0051 | 0.2783 | 0.9401 | 0.4652 | 0.6327 | 0.1137 | 0.9879 | 0.6158 | 0.5622 | 0.5926 | 0.9419 | 0.6411 |
| Uterus | 0.9183 | 0.6413 | 0.0329 | 0.6535 | 0.9739 | 0.4988 | 0.2166 | 0.5249 | 0.8721 | 0.1213 | 0.2441 | 0.6994 | 0.5356 | 0.5914 |
| Adipocytes | 0.9591 | 0.997 | 0.0342 | 0.0989 | 0.9963 | 0.0925 | 0.0049 | 0.084 | 0.9924 | 0.058 | 0.0073 | 0.0055 | 0.7039 | 0.1975 |
| Adipocytes (cultured, brown) | 0.967 | 0.9986 | 0.0289 | 0.0455 | 0.9963 | 0.1257 | 0.0022 | 0.0461 | 0.9915 | 0.0516 | 0.0054 | 0.0035 | 0.7039 | 0.1636 |
| Adipose | 0.9988 | 0.9649 | 0.2815 | 0.0329 | 0.9904 | 0.0998 | 0.0031 | 0.3958 | 0.9675 | 0.0859 | 0.0056 | 0.0084 | 0.7039 | 0.8603 |
| Blastocyst | 0.9996 | 0.9991 | 0.9801 | 0.6309 | 0.9963 | 0.3565 | 0.899 | 0.8879 | 0.9986 | 0.2635 | 0.945 | 0.8785 | 0.4133 | 0.0098 |
| Epidermis (E16.5) | 0.8545 | 0.484 | 0.6327 | 0.7617 | 0.5962 | 0.0628 | 0.5265 | 0.4237 | 0.9786 | 0.6515 | 0.2962 | 0.0709 | 0.9292 | 0.7966 |
| Skin (E16.5) | 0.9813 | 0.4608 | 0.0492 | 0.1896 | 0.5491 | 0.1472 | 0.039 | 0.2329 | 0.9931 | 0.25 | 0.0946 | 0.1047 | 0.7698 | 0.1749 |
| 4C Embryo (single-cell) | 0.9685 | 0.7847 | 0.9272 | 0.9386 | 0.9966 | 0.2313 | 0.9698 | 0.2765 | 0.9437 | 0.6898 | 0.891 | 0.9223 | 0.6756 | 0.1978 |
| 8C Embryo (single-cell) | 0.9906 | 0.665 | 0.2316 | 0.8709 | 0.9921 | 0.2548 | 0.5585 | 0.6419 | 0.9687 | 0.879 | 0.7588 | 0.9743 | 0.8613 | 0.3169 |
| Suprabasal epidermis (E14.5) | 0.9929 | 0.9961 | 0.882 | 0.8868 | 0.1753 | 0.0714 | 0.9622 | 0.8813 | 0.998 | 0.2838 | 0.0869 | 0.1501 | 0.8756 | 0.4408 |
| Epididymis | 0.9854 | 0.6191 | 0.05 | 0.7121 | 0.9691 | 0.6082 | 0.0102 | 0.846 | 0.9613 | 0.7362 | 0.1781 | 0.3099 | 0.7631 | 0.9698 |
| Genital fatpad | 0.9843 | 0.6502 | 0.3217 | 0.9695 | 0.407 | 0.0503 | 0.4345 | 0.8812 | 0.9606 | 0.8624 | 0.0423 | 0.5751 | 0.4551 | 0.9926 |
| Inner cell mass (E3.5) | 0.8769 | 0.692 | 0.9805 | 0.8474 | 0.9926 | 0.6237 | 0.9342 | 0.9393 | 0.9895 | 0.9045 | 0.7854 | 0.9881 | 0.5546 | 0.3857 |
| Kidney | 0.976 | 0.2359 | 0.124 | 0.7819 | 0.9871 | 0.4498 | 0.0129 | 0.9517 | 0.8863 | 0.5967 | 0.2204 | 0.6376 | 0.9888 | 0.9356 |
| Blastocyst (Late) TE | 0.7006 | 0.9093 | 0.9869 | 0.7731 | 0.9966 | 0.1198 | 0.9748 | 0.902 | 0.9972 | 0.9946 | 0.8045 | 0.9946 | 0.9938 | 0.2635 |
| Liver | 0.9691 | 0.2801 | 0.7575 | 0.8543 | 0.9675 | 0.0387 | 0.0379 | 0.954 | 0.8704 | 0.8207 | 0.1626 | 0.8711 | 0.8662 | 0.9689 |
| Mammary tissue | 0.9128 | 0.2201 | 0.0901 | 0.702 | 0.9575 | 0.0389 | 0.019 | 0.7263 | 0.8989 | 0.7389 | 0.1492 | 0.353 | 0.9564 | 0.9757 |
| Morula (single-cell) | 0.6579 | 0.6435 | 0.5157 | 0.848 | 0.9833 | 0.4463 | 0.9834 | 0.5333 | 0.9829 | 0.8483 | 0.9424 | 0.9502 | 0.68 | 0.396 |
| Morula | 0.9382 | 0.926 | 0.7553 | 0.7099 | 0.9957 | 0.5698 | 0.9348 | 0.8759 | 0.9973 | 0.7451 | 0.9328 | 0.9942 | 0.7909 | 0.6831 |
| Adipocytes (brown) | 0.9906 | 0.9424 | 0.6359 | 0.5176 | 0.9852 | 0.0303 | 0.0447 | 0.7925 | 0.9584 | 0.7415 | 0.1666 | 0.1334 | 0.9772 | 0.8808 |
| Adipocytes (white) | 0.7428 | 0.5212 | 0.0787 | 0.5233 | 0.9313 | 0.0384 | 0.0293 | 0.404 | 0.7479 | 0.6093 | 0.1365 | 0.0292 | 0.9638 | 0.9108 |
| Vas deferens | 0.981 | 0.4554 | 0.0568 | 0.8442 | 0.9563 | 0.6732 | 0.0373 | 0.646 | 0.8785 | 0.2788 | 0.3672 | 0.3703 | 0.9518 | 0.9698 |
| 4C Embryo | 0.6303 | 0.9949 | 0.9886 | 0.9938 | 0.9945 | 0.1395 | 0.9868 | 0.5671 | 0.9945 | 0.6888 | 0.8507 | 0.9421 | 0.7396 | 0.4658 |
| 8C Embryo | 0.9849 | 0.9949 | 0.9044 | 0.9937 | 0.9944 | 0.1512 | 0.9866 | 0.7705 | 0.8235 | 0.8642 | 0.8222 | 0.915 | 0.5873 | 0.5988 |
| B cell (germinal center) | 0.9909 | 0.9954 | 0.989 | 0.8295 | 0.2153 | 0.9796 | 0.5559 | 0.8879 | 0.2067 | 0.8808 | 0.9056 | 0.948 | 0.0456 | 0.8603 |
| B cell (resting) | 0.9783 | 0.9934 | 0.978 | 0.9211 | 0.1517 | 0.9836 | 0.6778 | 0.8879 | 0.2068 | 0.9297 | 0.8967 | 0.894 | 0.0764 | 0.814 |
| B cell (active) | 0.9849 | 0.9999 | 0.8067 | 0.8402 | 0.2058 | 0.9972 | 0.8636 | 0.8879 | 0.2151 | 0.87 | 0.9344 | 0.721 | 0.1972 | 0.257 |
| Basophilic erythroblast | 0.9728 | 0.9916 | 0.989 | 0.548 | 0.6802 | 0.8737 | 0.899 | 0.7951 | 0.8725 | 0.6114 | 0.945 | 0.948 | 0.7039 | 0.8603 |
| B cells CD43- | 0.9859 | 0.971 | 0.9044 | 0.5531 | 0.0723 | 0.9714 | 0.4029 | 0.8155 | 0.361 | 0.4667 | 0.2902 | 0.8014 | 0.3273 | 0.8603 |
| B cells CD19+ | 0.9946 | 0.8587 | 0.9801 | 0.73 | 0.1009 | 0.9668 | 0.3392 | 0.8879 | 0.1979 | 0.84 | 0.7694 | 0.948 | 0.009 | 0.8603 |
| Bone marrow | 0.8406 | 0.9652 | 0.915 | 0.6379 | 0.3549 | 0.7881 | 0.7342 | 0.5257 | 0.5113 | 0.4102 | 0.118 | 0.02 | 0.3273 | 0.1244 |
| B cell (naive) | 0.9917 | 0.997 | 0.976 | 0.8837 | 0.1053 | 0.9972 | 0.8636 | 0.8679 | 0.2064 | 0.892 | 0.8967 | 0.948 | 0.4427 | 0.8603 |
| Colon | 0.9914 | 0.9162 | 0.5133 | 0.3254 | 0.6624 | 0.5028 | 0.0787 | 0.532 | 0.5729 | 0.0509 | 0.0158 | 0.2198 | 0.9206 | 0.5298 |
| DC CD8+ | 0.1795 | 0.802 | 0.9869 | 0.9739 | 0.1647 | 0.4829 | 0.8695 | 0.4946 | 0.0364 | 0.9305 | 0.9884 | 0.181 | 0.5726 | 0.216 |
| Erythroblast | 0.8037 | 0.6557 | 0.961 | 0.7929 | 0.2809 | 0.7484 | 0.9484 | 0.8725 | 0.8949 | 0.9701 | 0.8882 | 0.7937 | 0.9808 | 0.9128 |
| Erythroid progenitor | 0.9076 | 0.467 | 0.9102 | 0.9277 | 0.2039 | 0.4581 | 0.9667 | 0.9161 | 0.7365 | 0.9473 | 0.9133 | 0.9052 | 0.921 | 0.9271 |
| Hematopoietic stem cells | 0.5724 | 0.7854 | 0.707 | 0.9177 | 0.2315 | 0.6009 | 0.8411 | 0.8699 | 0.7662 | 0.9483 | 0.9627 | 0.92 | 0.8211 | 0.8041 |
| Intestine | 0.959 | 0.3882 | 0.7494 | 0.8028 | 0.6765 | 0.3451 | 0.5 | 0.9509 | 0.2031 | 0.9398 | 0.3137 | 0.9294 | 0.8272 | 0.9771 |
| Liver (E14) | 0.832 | 0.2599 | 0.4922 | 0.7197 | 0.2393 | 0.4323 | 0.4342 | 0.9094 | 0.7984 | 0.91 | 0.6131 | 0.9109 | 0.8904 | 0.8424 |
| Liver (E14.5) | 0.8409 | 0.2107 | 0.3899 | 0.4748 | 0.2426 | 0.2266 | 0.5401 | 0.8177 | 0.7844 | 0.8362 | 0.4904 | 0.9 | 0.834 | 0.8254 |
| Megakaryocyte erythroid progenitor | 0.7275 | 0.472 | 0.9667 | 0.9347 | 0.1873 | 0.1013 | 0.9842 | 0.843 | 0.1629 | 0.9018 | 0.9754 | 0.923 | 0.7014 | 0.9156 |
| Polychromatic erythroblast | 0.5454 | 0.7086 | 0.9528 | 0.9248 | 0.6705 | 0.6393 | 0.9842 | 0.9787 | 0.6364 | 0.9709 | 0.9963 | 0.9934 | 0.9702 | 0.9904 |
| pre-B cells | 0.9365 | 0.8222 | 0.9693 | 0.9557 | 0.4717 | 0.8923 | 0.9835 | 0.9805 | 0.0676 | 0.9705 | 0.9965 | 0.9657 | 0.6877 | 0.9902 |
| pro-B cells | 0.5468 | 0.3258 | 0.9793 | 0.9457 | 0.1559 | 0.6308 | 0.978 | 0.9518 | 0.0428 | 0.9582 | 0.9859 | 0.8585 | 0.4192 | 0.9896 |
| pro-B (fraction B) | 0.6559 | 0.7721 | 0.9748 | 0.9434 | 0.1302 | 0.8692 | 0.9843 | 0.9382 | 0.1198 | 0.9627 | 0.9811 | 0.9028 | 0.6092 | 0.985 |
| pro-B (fraction CC') | 0.7021 | 0.7819 | 0.9585 | 0.921 | 0.1838 | 0.8759 | 0.9842 | 0.9798 | 0.159 | 0.9922 | 0.9908 | 0.9439 | 0.6451 | 0.9894 |
| Proerythroblast | 0.6272 | 0.8487 | 0.967 | 0.7868 | 0.3248 | 0.8438 | 0.9842 | 0.9795 | 0.7904 | 0.9688 | 0.9965 | 0.9935 | 0.9784 | 0.9893 |
| Promyelocytes | 0.845 | 0.1128 | 0.9693 | 0.9689 | 0.4533 | 0.1045 | 0.9605 | 0.8895 | 0.1085 | 0.9919 | 0.9935 | 0.9698 | 0.9122 | 0.9173 |
| Spermatocytes | 0.9295 | 0.9952 | 0.9368 | 0.6923 | 0.6803 | 0.9839 | 0.8045 | 0.9488 | 0.8519 | 0.9905 | 0.8977 | 0.9829 | 0.7748 | 0.7398 |
| splenic B cells | 0.7753 | 0.9895 | 0.5938 | 0.9575 | 0.1689 | 0.9938 | 0.9843 | 0.9608 | 0.1571 | 0.9759 | 0.9948 | 0.99 | 0.8809 | 0.9725 |
| Erythroid cells (TER119+) | 0.6298 | 0.5923 | 0.9287 | 0.7393 | 0.3571 | 0.9217 | 0.9156 | 0.9787 | 0.7939 | 0.9125 | 0.9935 | 0.9223 | 0.9743 | 0.988 |
| Cerebellar granular neurons | 0.9412 | 0.975 | 0.5216 | 0.0009 | 0.4234 | 0.8811 | 0.0406 | 0.1556 | 0.9924 | 0.6441 | 0.0001 | 0.0903 | 0.9902 | 0.0034 |
| Cortex neuron | 0.9754 | 0.9805 | 0.9862 | 0.0087 | 0.0695 | 0.9679 | 0.0383 | 0.0602 | 0.9841 | 0.0026 | 0.0096 | 0.433 | 0.9775 | 0.0525 |
| Cortical thymic epithelial | 0.9625 | 0.9994 | 0.0586 | 0.8295 | 0.2113 | 0.3653 | 0.0835 | 0.0074 | 0.3325 | 0.0079 | 0.0092 | 0.2502 | 0.8682 | 0.0298 |
| Cortical neurons (E16.5) | 0.9637 | 0.9922 | 0.9662 | 0.0447 | 0.1583 | 0.9581 | 0.4387 | 0.2909 | 0.997 | 0.0308 | 0.042 | 0.6793 | 0.9883 | 0.0157 |
| Basal epidermis (E14.5) | 0.9771 | 0.9961 | 0.493 | 0.8178 | 0.1227 | 0.2444 | 0.9134 | 0.5786 | 0.9971 | 0.1572 | 0.0216 | 0.5481 | 0.5677 | 0.418 |
| Fatpad | 0.8752 | 0.5769 | 0.2471 | 0.9643 | 0.441 | 0.0461 | 0.2956 | 0.9521 | 0.9451 | 0.7228 | 0.0283 | 0.3792 | 0.9818 | 0.9932 |
| Hair follicle | 0.9783 | 0.9544 | 0.0663 | 0.8394 | 0.2443 | 0.1778 | 0.1106 | 0.1649 | 0.9978 | 0.8836 | 0.0101 | 0.669 | 0.7464 | 0.8472 |
| Heart | 0.9658 | 0.1736 | 0.1621 | 0.1032 | 0.9513 | 0.8092 | 0.1286 | 0.7884 | 0.899 | 0.5816 | 0.0711 | 0.405 | 0.988 | 0.8478 |
| Hippocampus neuron | 0.4975 | 0.4751 | 0.9051 | 0.0666 | 0.1488 | 0.893 | 0.2481 | 0.7109 | 0.8773 | 0.2318 | 0.0695 | 0.9633 | 0.9079 | 0.773 |
| Hippocampus tissue | 0.9778 | 0.3104 | 0.9057 | 0.0639 | 0.1205 | 0.9506 | 0.1319 | 0.5801 | 0.92 | 0.1733 | 0.0253 | 0.8658 | 0.975 | 0.6926 |
| Keratinocyte | 0.1544 | 0.8106 | 0.5887 | 0.9674 | 0.9966 | 0.4404 | 0.9745 | 0.6832 | 0.9973 | 0.5782 | 0.2482 | 0.8137 | 0.9595 | 0.9109 |
| Large intestine | 0.9702 | 0.9139 | 0.8912 | 0.7741 | 0.855 | 0.3661 | 0.7011 | 0.9023 | 0.5011 | 0.9119 | 0.2405 | 0.9897 | 0.9079 | 0.9841 |
| Lower molar | 0.7923 | 0.8071 | 0.1122 | 0.2295 | 0.1122 | 0.9737 | 0.1381 | 0.0136 | 0.9682 | 0.6573 | 0.044 | 0.1096 | 0.2474 | 0.3513 |
| Lung | 0.4173 | 0.1611 | 0.1036 | 0.9148 | 0.9605 | 0.1399 | 0.0769 | 0.8065 | 0.5609 | 0.6345 | 0.0803 | 0.1836 | 0.619 | 0.5118 |
| Neocortex cortical plate | 0.7756 | 0.976 | 0.9741 | 0.0442 | 0.0304 | 0.9929 | 0.6936 | 0.4412 | 0.9946 | 0.059 | 0.0072 | 0.7289 | 0.9573 | 0.0562 |
| Neocortex subventricular zone | 0.7865 | 0.8604 | 0.9856 | 0.0819 | 0.0473 | 0.9848 | 0.6338 | 0.2869 | 0.9827 | 0.0259 | 0.0103 | 0.7903 | 0.9042 | 0.0686 |
| Neural progenitor cell | 0.8504 | 0.996 | 0.6736 | 0.0163 | 0.8997 | 0.7548 | 0.8745 | 0.2483 | 0.9905 | 0.4178 | 0.0352 | 0.206 | 0.7648 | 0.9515 |
| Embryonic kidney fibroblast | 0.9858 | 0.6464 | 0.3649 | 0.3197 | 0.8664 | 0.714 | 0.931 | 0.1541 | 0.8076 | 0.355 | 0.1295 | 0.9014 | 0.7226 | 0.6786 |
| Prostate basal cells | 0.97 | 0.723 | 0.4817 | 0.6453 | 0.8869 | 0.7693 | 0.7291 | 0.6261 | 0.6277 | 0.875 | 0.1197 | 0.9021 | 0.4351 | 0.9719 |
| Skin epithelial | 0.6476 | 0.971 | 0.6898 | 0.9249 | 0.104 | 0.0857 | 0.9406 | 0.0821 | 0.9968 | 0.7547 | 0.0479 | 0.2154 | 0.5569 | 0.9519 |
| Striated muscle | 0.9762 | 0.4775 | 0.0927 | 0.1537 | 0.9819 | 0.6903 | 0.6269 | 0.4413 | 0.957 | 0.928 | 0.1089 | 0.266 | 0.8932 | 0.9125 |
| Telencephalon | 0.7002 | 0.9863 | 0.9737 | 0.051 | 0.051 | 0.9931 | 0.8365 | 0.2586 | 0.9914 | 0.1932 | 0.0859 | 0.4546 | 0.9506 | 0.0253 |
| Whole brain (E14.5) | 0.7737 | 0.6867 | 0.5526 | 0.03 | 0.0489 | 0.9681 | 0.4125 | 0.0791 | 0.986 | 0.0553 | 0.0249 | 0.2522 | 0.8185 | 0.0457 |
| Whole brain (E18.5) | 0.9386 | 0.6959 | 0.3734 | 0.021 | 0.0598 | 0.9786 | 0.2692 | 0.3952 | 0.9901 | 0.0639 | 0.0366 | 0.4959 | 0.9023 | 0.0378 |
